# Supplementary material for: Assessment of climate change impact on the malaria vector Anopheles hyrcanus, West Nile disease, and incidence of melanoma in the Vojvodina Province (Serbia) using data from a regional climate model
Source: PLoS One. 2020 Jan 15;15(1):e0227679. doi: 10.1371/journal.pone.0227679 (PMC6961917; doi:10.1371/journal.pone.0227679)
Supplement: S3 Table — (DOCX) [file pone.0227679.s003.docx]

| No. | Town | Trap position* | | Year | | | | | | Total years |
| --- | --- | --- | --- | --- | --- | --- | --- | --- | --- | --- |
|  |  | LAT | LON | 2010 | 2011 | 2012 | 2013 | 2014 | 2015 |  |
| 1 | Batrovci | 45.046 | 19.108 | 0(3) | 0(3) | 0(5) | 1(6) | 0(7) | 0(7) | 1 |
| 2 | Bukovac | 45.217 | 19.883 | 0(2) | 0(3) | 0(4) | 0(6) | 1(7) | 0(7) | 1 |
| 3 | Hrtkovci | 44.874 | 19.762 | 0(3) | 0(3) | 0(5) | 1(5) | 0(7) | 0(7) | 1 |
| 4 | Perlez | 45.215 | 20.377 | 0(3) | 0(3) | 0(5) | 0(6) | 1(6) | 0(7) | 1 |
| 5 | Karavukovo | 45.500 | 19.183 | 0(3) | 0(3) | 0(5) | 0(6) | 1(7) | 1(7) | 2 |
| 6 | Lovćenac | 45.667 | 19.667 | 0(3) | 0(3) | 0(5) | 0(6) | 1(7) | 1(7) | 2 |
| 7 | Mali Iđoš | 45.700 | 19.650 | 0(3) | 0(3) | 0(5) | 1(6) | 1(7) | 0(7) | 2 |
| 8 | Novi Bečej | 45.599 | 20.143 | 0(3) | 0(3) | 0(5) | 1(5) | 1(7) | 0(7) | 2 |
| 9 | Novi Sad | 45.250 | 19.800 | 0(3) | 0(3) | 0(5) | 1(6) | 0(7) | 1(6) | 2 |
| 10 | Novi Sad | 45.263 | 19.810 | 1(2) | 1(3) | 0(5) | 1(6) | 0(7) | 0(7) | 3 |
| 11 | Petrovaradin | 45.233 | 19.867 | 1(3) | 1(3) | 1(4) | 1(6) | 0(6) | 1(7) | 5 |

S3 Table Frequency of sampling of WNV infected mosquitoes (1 – 5 times) in the Vojvodina Province, Serbia, during the period 2010-2016

0, no WNV positive pool detected at specific position during particular season; 1, one or more WNV positive pools detected at specific position during particular season; (x), number in brackets indicates number of samplings during the season (May-September).

* dry ice baited NS2 traps without light placed at precisely the same position during 5 years
