# Supplementary material for: Assessment of climate change impact on the malaria vector Anopheles hyrcanus, West Nile disease, and incidence of melanoma in the Vojvodina Province (Serbia) using data from a regional climate model
Source: PLoS One. 2020 Jan 15;15(1):e0227679. doi: 10.1371/journal.pone.0227679 (PMC6961917; doi:10.1371/journal.pone.0227679)
Supplement: Attachment — Submitted filename: PONE-D-19-16900_reviewer.pdf [file pone.0227679.s005.pdf]

PLOS ONE

Assessment of climate change impact on malaria vectors, West Nile disease, and incidence of melanoma in the Vojvodina Province (Serbia) using data from a regional climate model

--Manuscript Draft--

**Manuscript Number:**	PONE-D-19-16900
**Article Type:**	Research Article
**Full Title:**	Assessment of climate change impact on malaria vectors, West Nile disease, and incidence of melanoma in the Vojvodina Province (Serbia) using data from a regional climate model
**Short Title:**	Impact of climate change on malaria vectors, West Nile disease, and incidence of melanoma
**Corresponding Author:**	Mina Petric, MSc<br>Universiteit Gent<br>Antwerpen, Antwerpen BELGIUM
**Keywords:**	Climate Change; West Nile Virus; Culex pipiens; Anopheles hyrcanus; UV radiation; Regional Climate Model; Melanoma incidence
**Abstract:**	Motivated by the One Health paradigm, we found the expected changes in temperature and UV radiation (UVR) to be a common trigger for enhancing the risk that viruses, vectors and diseases pose to human and animal health. We compare data from the mosquito field collections and medical studies with regional climate model projections to examine the impact of climate change on the circulation of West Nile virus (WNV), the spreading of the malaria vector and the incidence of melanoma. We analysed data obtained from ten selected years of standardized mosquito vector sampling with 219 unique location-year combinations, and 10-years of melanoma incidence. Trends in the observed data were compared to the climatic variables obtained by the coupled regional Eta Belgrade University and Princeton Ocean Model for the period 1961-2015 using the A1B scenario, and the expected changes up to 2030 were presented. The frequency of WNV detections in Culex pipiens was significantly correlated to overwintering temperature averages and seasonal relative humidity at the sampling sites. Regression model projects a twofold increase in the incidence of WNV positive Culex. pipiens for a rise of 0.5°C in overwintering TOctober-April temperatures. Spreading and relative abundance of Anopheles hyrcanus was positively correlated with the trend of the mean annual temperature. We anticipated a nearly twofold increase in the number of invaded sites up to 2030. The projected increase of 56% in the number of days with Tmax ≥ 30ºC (HD) and UVR doses (up to 1.2%) corresponds to an increasing trend in melanoma incidence. Simulations of the Pannonian countries climate anticipate warmer and drier conditions with possible dominance of temperature and number of HD over other ecological factors. These signal the importance of monitoring the changes to the preparedness of mitigating the risk.
**Order of Authors:**	Dragutin T. Mihailović
	Dušan Petrić
	Tamaš Petrović
	Ivana Hrnjaković Cvjetković
	Vladimir Đurđević
	Emilija Nikolić-Đorić
	Ilija Arsenić
	Mina Petrić, MSc
	Gordan Mimić
**Additional Information:**	
**Question**	**Response**

Powered by Editorial Manager® and ProduXion Manager® from Aries Systems Corporation

**Financial Disclosure**<br><br>Enter a financial disclosure statement that describes the sources of funding for the work included in this submission. Review the submission guidelines for detailed requirements. View published research articles from *PLOS ONE* for specific examples.<br><br>This statement is required for submission and **will appear in the published article** if the submission is accepted. Please make sure it is accurate.<br><br>**Unfunded studies**<br>Enter: *The author(s) received no specific funding for this work.*<br><br>**Funded studies**<br>Enter a statement with the following details:<br>• Initials of the authors who received each award<br>• Grant numbers awarded to each author<br>• The full name of each funder<br>• URL of each funder website<br>• Did the sponsors or funders play any role in the study design, data collection and analysis, decision to publish, or preparation of the manuscript?<br>• **NO** - Include this sentence at the end of your statement: *The funders had no role in study design, data collection and analysis, decision to publish, or preparation of the manuscript.*<br>• **YES** - Specify the role(s) played.<br><br>* typeset	This paper was realized as a part of the project "Studying climate change and its influence on the environment: impacts, adaptation and mitigation" (43007) financed by the Ministry of Education and Science of the Republic of Serbia, within the framework of integrated and interdisciplinary research for the period 2011-2017. Historical data for mosquito vectors are the outputs of the projects supported by the Veterinary Directorate, Ministry of Agriculture and Environment Protection, Republic of Serbia, Provincial Secretariat for Science and Technological Development, AP Vojvodina; TR31084 MESRS. The works of Dušan Petrić and Mina Petrić were done under the frame of EurNegVec COST Action TD1303. The funders had no role in study design, data collection and analysis, decision to publish, or preparation of the manuscript.
**Competing Interests**<br><br>Use the instructions below to enter a competing interest statement for this submission. On behalf of all authors, disclose any competing interests that could be perceived to bias this work—acknowledging all financial support and any other relevant financial or non-financial competing interests.<br><br>This statement **will appear in the published article** if the submission is accepted. Please make sure it is	The authors have declared that no competing interests exist.

Powered by Editorial Manager® and ProduXion Manager® from Aries Systems Corporation

accurate. View published research articles from *PLOS ONE* for specific examples.<br><br>**NO authors have competing interests**<br><br>Enter: *The authors have declared that no competing interests exist.*<br><br>**Authors with competing interests**<br><br>Enter competing interest details beginning with this statement:<br><br>*I have read the journal's policy and the authors of this manuscript have the following competing interests: [insert competing interests here]*<br><br>* typeset	
**Ethics Statement**<br><br>Enter an ethics statement for this submission. This statement is required if the study involved:<br><br>• Human participants<br>• Human specimens or tissue<br>• Vertebrate animals or cephalopods<br>• Vertebrate embryos or tissues<br>• Field research<br><br>Write "N/A" if the submission does not require an ethics statement.<br><br>General guidance is provided below. Consult the submission guidelines for detailed instructions. **Make sure that all information entered here is included in the Methods section of the manuscript.**	N/A

**Format for specific study types**<br><br>**Human Subject Research (involving human participants and/or tissue)**<br>• Give the name of the institutional review board or ethics committee that approved the study<br>• Include the approval number and/or a statement indicating approval of this research<br>• Indicate the form of consent obtained (written/oral) or the reason that consent was not obtained (e.g. the data were analyzed anonymously)<br><br>**Animal Research (involving vertebrate animals, embryos or tissues)**<br>• Provide the name of the Institutional Animal Care and Use Committee (IACUC) or other relevant ethics board that reviewed the study protocol, and indicate whether they approved this research or granted a formal waiver of ethical approval<br>• Include an approval number if one was obtained<br>• If the study involved *non-human primates*, add *additional details* about animal welfare and steps taken to ameliorate suffering<br>• If anesthesia, euthanasia, or any kind of animal sacrifice is part of the study, include briefly which substances and/or methods were applied<br><br>**Field Research**<br><br>Include the following details if this study involves the collection of plant, animal, or other materials from a natural setting:<br>• Field permit number<br>• Name of the institution or relevant body that granted permission	
**Data Availability**<br><br>Authors are required to make all data underlying the findings described fully available, without restriction, and from the time of publication. PLOS allows rare exceptions to address legal and ethical concerns. See the PLOS Data Policy and FAQ for detailed information.	Yes - all data are fully available without restriction

Powered by Editorial Manager® and ProduXion Manager® from Aries Systems Corporation

A Data Availability Statement describing where the data can be found is required at submission. Your answers to this question constitute the Data Availability Statement and **will be published in the article**, if accepted.<br><br>**Important:** Stating 'data available on request from the author' is not sufficient. If your data are only available upon request, select 'No' for the first question and explain your exceptional situation in the text box.<br><br>Do the authors confirm that all data underlying the findings described in their manuscript are fully available without restriction?	
**Describe where the data may be found in full sentences. If you are copying our sample text, replace any instances of XXX with the appropriate details.**<br><br>• If the data are **held or will be held in a public repository**, include URLs, accession numbers or DOIs. If this information will only be available after acceptance, indicate this by ticking the box below. For example: *All XXX files are available from the XXX database (accession number(s) XXX, XXX.).*<br>• If the data are all contained **within the manuscript and/or Supporting Information files**, enter the following: *All relevant data are within the manuscript and its Supporting Information files.*<br>• If neither of these applies but you are able to provide **details of access elsewhere**, with or without limitations, please do so. For example:<br><br>*Data cannot be shared publicly because of [XXX]. Data are available from the XXX Institutional Data Access / Ethics Committee (contact via XXX) for researchers who meet the criteria for access to confidential data.*<br><br>*The data underlying the results presented in the study are available from (include the name of the third party*	All relevant data are within the manuscript and its Supporting Information files.

and contact information or URL).<br>• This text is appropriate if the data are owned by a third party and authors do not have permission to share the data.<br><br>* typeset	
Additional data availability information:	

Manuscript Click here to download Manuscript PLOS_MIHAILOVIC_ET_AL_2019.docx

Assessment of climate change impact on malaria vectors, West Nile disease, and incidence of melanoma in the Vojvodina Province (Serbia) using data from a regional climate model

Dragutin T. Mihailović[1], Dušan Petrić[2], Tamaš Petrović[3], Ivana Hrnjaković Cvjetković[4,5], Vladimir Đurđević[6], Emilija Nikolić-Đorić[7], Ilija Arsenić[1], Mina Petrić[8,9,10], Gordan Mimić[11]

[1] Department of Field and Vegetable Crops, Faculty of Agriculture, University of Novi Sad, Novi Sad, Serbia

[2] Department of Plant and Environment Protection, Faculty of Agriculture, University of Novi Sad, Novi Sad, Serbia

[3] Department for virology, Scientific Veterinary Institute "Novi Sad", Novi Sad, Serbia

[4] Institute of Public Health of Vojvodina, Novi Sad, Serbia

[5] Faculty of Medicine, University of Novi Sad, Novi Sad, Serbia

[6] Institute of Meteorology, Faculty of Physics, University of Belgrade, Belgrade, Serbia

[7] Department of Agricultural Economics, Faculty of Agriculture, University of Novi Sad, Novi Sad, Serbia

[8] Avia-GIS NV, Zoersel, Belgium

[9] Department of Physics, Faculty of Sciences, University of Novi Sad, Novi Sad, Serbia

[10] Department of Physics and Astronomy, Faculty of Sciences, University of Gent, Gent, Belgium

[11] BioSense Institute, University of Novi Sad, Novi Sad, Serbia

E-mail: guto@polj.uns.ac.rs

Received xxxxxx

Accepted for publication xxxxxx

Published xxxxxx

Abstract

Motivated by the One Health paradigm, we found the expected changes in temperature and UV radiation (UVR) to be a common trigger for enhancing the risk that viruses, vectors and diseases pose to human and animal health. We compare data from the mosquito field collections and medical studies with regional climate model projections to examine the impact of climate change on the circulation of West Nile virus (WNV), the spreading of the malaria vector and the incidence of melanoma. We analysed data obtained from ten selected years of standardized mosquito vector sampling with 219 unique location-year combinations, and 10-years of melanoma incidence. Trends in the observed data were compared to the climatic variables obtained by the coupled regional Eta Belgrade University and Princeton Ocean Model for the period 1961-2015 using the A1B scenario, and the expected changes up to 2030 were presented. The frequency of WNV detections in *Culex pipiens* was significantly correlated to overwintering temperature averages and seasonal relative humidity at the sampling sites. Regression model projects a twofold increase in the incidence of WNV positive *Culex. pipiens* for a rise of 0.5°C in overwintering $T_{October\text{-}April}$ temperatures. Spreading and relative abundance of *Anopheles hyrcanus* was positively correlated with the trend of the mean annual temperature. We anticipated a nearly twofold increase in the number of invaded sites up to 2030. The projected increase of 56% in the number of days with $T_{max} \geq 30°$ C (HD) and UVR doses (up to 1.2%) corresponds to an increasing trend in melanoma incidence. Simulations of the Pannonian countries climate anticipate warmer and drier conditions with possible dominance of temperature and number of HD over other ecological factors. These signal the importance of monitoring the changes to the preparedness of mitigating the risk.

Introduction

Climate change is referred to as “the biggest global health threat of the 21st century” [1]. The analysis of outputs from all general circulation models (GCM) suggest that the countries of the Pannonian Plain, including Serbia, are facing significant impacts of climate change, affecting all aspects of human life [2].

The authors (meteorology, entomology, veterinary medicine and public health experts), have been working together since 2003, promoting the idea of multisectoral collaboration before the One Health Concept was officially inaugurated in the USA in 2007 [3], and endorsed by the EU [4] as well as prominent organizations such as the World Health Organization, Food and Agriculture Organization and the World Organization for Animal Health (OIE) in 2018 [5].

In this paper, the authors collected and analysed observed data collected over a period of 31 years and related a subset to outputs from a Regional Climate Model (RCM). Vector-borne diseases and melanoma significant climate-driven threats for which risk sources can be clearly defined [6]. Moreover, both present progressively growing environmental threats to animal as well as human health in the countries of the Pannonian Plane.

The biology and distribution of mosquito vectors and their capacity to transmit mosquito-borne diseases are dependent on many factors such as global trade and travel, urbanization, habitat destruction, pesticide application, host density and climate. *Culex pipiens* and *Anopheles hyrcanus* are mosquito species that are vectors of West Nile virus (WNV) disease and malaria, respectively, the two most detrimental vector-borne diseases worldwide [7]. In 2018, Serbia was the second European country (after Italy) most affected by WNV disease (415 reported cases with 35 fatal outcomes [8]). Malaria was eradicated from Serbia and other Balkan states during the last century. However, the spreading of its vectors (*Anopheles* mosquitoes) and the re-emergence of the disease in Greece [9] pose a threat to South East and Central Europe once again. Current evidence suggests that inter-annual and inter-decadal climate variability have a direct influence on the epidemiology of vector-borne diseases, with temperature and relative humidity as the principal abiotic factors influencing the life-cycles of the mosquito vector, the pathogen, the host and the interactions between them [10,11].

Melanoma is a malignant disease that has experienced a significant increase in incidence during the last few decades all over the world [12]. The climate change impact on melanoma should be considered as a synergy of changes in UV radiation (UVR) due to stratospheric ozone depletion and the long-term increase of air temperature leading to more prolonged exposure of individuals to UVR doses and consequently to a

higher risk of melanoma [13]. The melanoma mortality in the Vojvodina Province (northern Serbia) (VPS) in the period 1985-2004 shows an evident increase, placing it amongst the most vulnerable regions in the world. Thus, Jovanović et al. [14] estimated and made the list of mortality rates from malignant melanoma for males (age-standardized rate/100,000) in Europe (39 countries) for the year 2000, using ENCR data. This list shows that the VPS is among the top eleven states (six of them having parts in the Pannonian Plane) listed as the most endangered.

In this study, devoted to revealing the potential impact of climate change on animal and human health, we compare a considerable amount of previously unpublished ecological data obtained from the field and clinical surveys with climate change projections for the VPS, which is representative of the Central European low-altitude areas with a human-dominated landscape (Fig 1). We examined the "microclimate" differentiation between sites with a specific frequency of WNV occurrence in *Cx. pipiens* and effects of temperature on the spread and relative abundance of the malaria vector *An. hyrcanus*. We also evaluated the impact of climate change on melanoma incidence as a synergy of changes in UVR doses and the long-term increase in the number of hot days (HD), with daily maximum temperature > 30°C using the Eta Belgrade University and Princeton Ocean Model (EBU-POM) regional model data.

Fig 1. (a) Location of the Vojvodina Province (Serbia) in Europe and (b) altitude map.

Materials and Methods

For the assessment of the climate change and the impact of UVR doses, we used the climatic variables obtained by the coupled regional EBU-POM model for the historical period 1961-2000 and the period 2001-2030 using the SRES-A1B scenario.

Study area and climate

The VPS is situated in the northern part of Serbia and the southern part of the Pannonian lowland (18°51′–

21°33′E, 44°37′–46°11′N and 75–641 m.a.s.l. (with the Fruška Gora Mountain in the south) as it is seen in Fig 1a and Fig 1b). This region is the essential food production area in Serbia with a total surface area of 21,500 km^2 and a population of about 2 million. This region has a continental climate, with elements of a sub-humid and warm climate (Cfwbx" according to Köppen classification).

Models and formula used

The global and regional climate model

For climate simulations in this study, we used results of the EBU-POM model runs for the SRES-A1B scenario integrated over the period 2001–2030 [15]. The EBU-POM is a two-way, coupled RCM. The atmospheric part is the Eta/National Centres for Environmental Prediction (NCEP) limited area model (resolution 0.25° × 0.25° on 32 vertical levels; centred at 41.5° N, 15° E, with boundaries at ±19.9° W–E and ±13.0° S–N), while the oceanic part is the POM (resolution 0.20° × 0.20° on 21 vertical levels). The driving global circulation model (GCM) was the ECHAM5 model [16] coupled with the Max Planck Institute Ocean Model (MPI-OM) [17]. More details about model integrations and performed bias correction for VPS can be found in the paper by Mihailović et al. [2]. The POM model was set over the Mediterranean Sea without the Black Sea; for other open seas, the sea surface temperature from the GCM was used as a bottom boundary condition.

Empirical formulae

For calculating the daily doses of UVR, i.e. UVRD in the study area sites we have used the following empirical formula $UVRD = 0.002507\ \times\ G_d - 5.985$ (kJ/m2) derived by Malinović-Milićević et al. [18], where G_d is the daily sum of the global solar radiation.

Environmental sampling

Mosquito vectors

We used standardized protocols to measure mosquito presence/absence, density and infestation by WNV.

Data are extracted from dry ice-baited trap samples, collected over 31 years at 166 different sites (745 sampled locations, S1 Table) in the VPS, to infer on the trends of local vector status and virus circulation in mosquitoes. In all years mosquitoes were sampled from May to September, with different spatial intensity and time-frequency governed by the scale and scope of different research projects. For comparison with climate variables, we extracted data obtained in 10 years (1985 – 6, 2004 – 2005 and 2010 – 2015) for which a standardized surveillance protocol was in place. These periods have the highest number of particular location-year combinations (S1-S3 Tables).

Samples were collected by two different types of dry-ice baited suction traps. During 1985 and 1986 [19,20] by the miniature CDC light trap (CDC) and for 2004 and 2015 by the NS2 trap (our own design of dry ice-baited suction trap without light). Both traps were operating without a light source (incandescent light proved not to be attractive/repellent for most mosquito species inhabiting the VPS [20]. The CDC trap has a 3 - 5 times stronger suction power (operated by a 9 V battery) than NS2 (operated by 3 x 1.2 V batteries), meaning that the increase in density of species observed after 1986 could not be attributed to the change of the type of trap. Traps were operated from the afternoon until the morning of the next day (one trap night), with different periodicity. The specific location of the trap at each site was chosen by experienced entomologists to stabilize variation of the collected data.

We used three parameters to indicate *An. hyrcanus* spread and population growth in the period 1985-2015: i) the ratio of positive to total mosquito samplings per year; ii) the number of sites invaded (positive places where it was looked for, but was not found in the preceding sampling period, and the number of sites where was observed in both periods, i.e. established); and iii) the average number of specimens sampled in one trap during single sampling period from the afternoon of the starting day to the morning of the next day (Fig 2a and Fig 2c). Here, we used data from 1,073 mosquito samples (1985-6, 2004-5 and 2014-5), obtained at 54 location over 6 years (142 unique location-year combinations) (S2 Table).

Fig 2. (a) The CRCM projection of the mean annual air temperature (Ta) for the period 1985 - 2030 and: i) number of specimens sampled in one trap during single sampling period (light blue

columns); ii) the number of sites invaded by *An. hyrcanus* (red columns); and iii) relative number of positive samplings per year (green columns), (b) projected increase in the number of sites invaded by *An. hyrcanus* (the period 2001-2030 ±S.E.), and (c) projected increase in the number of the specimens sampled in one trap during single sampling period (2001 - 2030 ±S.E.).

For *Cx. pipiens*, the period starting with the first detection of WNV in mosquitoes in Serbia, in 2010, (Petrić et al. [21]) to 2015 was considered. For detection of WNV, specimens were sampled, anaesthetized by dry ice, identified to species level [22] on dry ice cooled paper, pooled according to date, location, sex and species, transported on dry ice to the laboratory and stored at -70°C before virus detection. Pool size did not exceed 50 mosquito specimens per pool. Virus detection was performed according to procedures described in Petrovć et al. [23]. We analyzed the yearly occurrence of the WNV positive *Cx. pipiens* mosquitoes sampled by dry ice-baited traps in the years 2010-2015 across 77 unique location-year combinations (S3 Table). Only traps positioned exactly at the same spot over the entire six-year period are considered for analysis. Numbers allocated to different places (Fig 3) indicate the number of years in the period 2010-2015 in which WNV was detected in sampled *Cx. pipiens* mosquitoes; e.g. 5 indicates that WNV positive *Cx. pipiens* were detected in five out of the six years in the samples collected from the same spot.

Fig 3. (a) Dependence of frequencies (λ) of WNV positive *Culex pipiens* detections at the same site on overwintering temperatures (Toa); (b) Frequency of sampling of WNV infected mosquitoes (1 – 5 times) during six years (bars and numbers) in NUTS3 (Nomenclature of Territorial Units for Statistics) units of the Vojvodina Province, Serbia.

Melanoma incidence and UVR

Indicators for a ten-year period 1995 - 2004 of melanoma incidence in women and men based on the data

obtained from the Cancer Registry of Vojvodina following the methodology of Jovanović et al. [14] were used for the analysis.

Statistics

We considered the papers [2,24] in which Kolmogorov complexity measures and sample entropy [25] were used to quantify the regularity and complexity of air temperature and precipitation time series, obtained by the EBU-POM model, representing both deterministic chaos and stochastic processes. Then, the obtained results were compared with the same information measures using data taken from daily meteorological reports of the Republic Hydrometeorological Service of Serbia. For *An. hyrcanus*, the temperature trend was evaluated by the Mann-Kendall test using the R statistical package [26]. Field observed values on species distribution and density for the period 1985-2015 and forecasts of the numbers of sites invaded and specimens sampled for the period 2016 - 2030 based on linear trend were obtained by the Eviews 9.5 software [27]. For *Cx. pipiens*, the relationship between yearly frequency of WNV detection in mosquitoes, air temperature and relative humidity (derived from the climate model) was estimated using Spearman's Rank-Order Correlation and a Poisson regression model (Statistica 13 [28]).

Results

Mosquito vectors. Figure 2a shows an evident linear trend of the mean annual temperature T_a for the period 1985 - 2030 ($r = 0.467$; $p = 0.001$; $\tau = 0.328$) calculated from the EBU-POM regional model outputs for 29 representative sites in the VPS. All parameters that were chosen for the evaluation of the spread and population increase of *An. hyrcanus* were positively, but to a different extent, correlated to the time argument (periods in which sampling was performed since the beginning of monitoring in 1985) indicating a monotonic trend. The increase of parameters follows the trend of T_a (Fig 2a). The strongest correlation was found for the increase in the ratio of positive samplings ($r = 0.986$; $p < 0.001$; $\tau = 0.828$), followed by the number of mosquitoes per trap night ($r = 0.919$; $p < 0.05$; $\tau = 0.733$) , and the number of sites invaded ($r = 0.889$; $p < 0.05$; $\tau = 0.6$). By 2030 we anticipate a further increase in numbers of invaded sites and

adult females sampled, by 1.71 and 1.27 fold, respectively (Fig 2b and Fig 2c).

To investigate the impact of microclimate on the complex interaction between *Cx. pipiens* and WNV, we used the following climatic parameters from the EBU-POM model outputs (covering the period 2006-2015) for 11 sites (GPS coordinates – S3 Table) in the VPS with different histories of WNV circulation: (i) mean annual temperature (T_a); (ii) overwintering temperature (T_{oa}) for the period October – April; and (iii) seasonal temperature (T_{ms}) and relative humidity (R_{ms}) for the period May – September. For these sites, we examined the correlation between the frequency of WNV detections in *Cx. pipiens* at each site (from 2010, when WNV was detected for the first time in the mosquito vector *Cx. pipiens* in Serbia, to 2015) and the corresponding period averages of climate time series for the same site. Spearman rank order correlation of the mean values was the highest for T_{oa} ($r = 0.755$; $p < 0.05$), then for T_a ($r = 0.616$; $p < 0.05$), R_{ms} ($r = 0.499$; $p < 0.05$) and T_{ms} ($r = 0.477$; $p < 0.05$). Figure 3a depicts the Poisson regression model for the dependence of a number of detections per site (frequency - λ) on T_{oa}, which is highly significant ($p < 0.05$). The output of the model ($ln\lambda = -7.923 + 1.533 \times T_{OA}$) indicates that for an increase of 0.5°C in T_{oa} (presuming that all other factors needed for the circulation of WNV are kept constant), a twofold increase in the incidence of WNV positive *Cx. pipiens* could be projected. Figure 3b depicts that most of the sites with the high frequency of WNV occurrence (≥ 2) were distributed along the northwest-southeast axis of the VPS.

Melanoma incidence and UVR doses. We have used the model simulation to study the expected impact of climate change on UVR exposure of human skin for nine sites in VPS [PA (Palić), SO (Sombor), KI (Kikinda), NS (Novi Sad), BC (Bečej, ZR (Zrenjanin), SM (Sremska Mitrovica), BK (Bantaski Karlovac) and BG (Beograd)]. Firstly, we calculated daily UVR doses (UVRD) from global radiation model outputs using the empirical formula for the seven aforementioned counties for the period April-September, and then we found the relative change R(UVRD) of those doses as $R(UVRD) = (UVRD-UVRD_k)/ UVRD_k$ where $UVRD_k$ is the dose for the 1961–1990 reference period, while the UVRD is calculated for the period 2001-2030. Figure 4b shows the positive relative change of UVRD, remarkably covering an eastern, southern,

western and partly central area of VPS. Specifically, the projected increase is twofold going from the west and northwest (0.60%) towards the east and southeast where it reaches values of about 1.20%. The EBU-POM model (for nine sites) shows a significant expected increase of 56% in the number of HD days in the VPS (Fig 4a), compared to the period 1961 – 1990. Additionally, we observed a decrease of 1.1% in the number of days with maximum air temperature higher than 25°C (warm days - WD). This prolongs the exposure of outdoor working adults to UVR and thus leads to the increase in melanoma risk. This risk becomes even more significant because of the increase in cumulative values of UVR doses (Fig 4c). Figure 4d depicts the cumulative incidence of melanoma for the period 1985 - 2004 with an increasing monotonic trend ($r = 0.970$, $p < 0.001$).

Fig 4. Relative change of hot days (HD) (a) and UVR radiation doses [R (UVRD)] (b) for the period 2001-2030 compared to the period 1961-1990, (c) cumulative values of mean UVR doses for the period 1985-2030 (averaged for seven sites: PA, SO, BC, KI, NS, ZR, SM and BK) under the SRES-A1B scenario [for WD and HD days] and (d) cumulative incidence of melanoma for the period 1995 – 2004 (ja bih izabrao ovakav zapis) in the Vojvodina Province, Serbia.

Discussion

Here we presented an intriguing comparison of the impact of climate change on complex systems including mosquito vectors, pathogens and humans, which are all indicators of the risk imposed on human health. Our objectives were to use historical, previously unpublished sets of entomological and clinical data and examine the importance of temperature in contributing to the spreading of the malaria vector *An. hyrcanus*; to differentiate between sites with a specific frequency of WNV occurrence in *Cx. pipiens* and to assess the impact of increasing UVR and HD on melanoma incidence using the EBU-POM regional model data. A similar approach was recently used in observing the dramatic decline in total flying insect biomass in protected areas in Germany [29].

Mosquito vectors. Until the end of the 20th century, northern Serbia was considered the northern limit for

the distribution of *An. hyrcanus* in Europe. The first detection in Serbia dates from 1979 [30] from the north part of VPS. We found it in the central part of the Province in 1985 and since then have been noticing its continued spread. The several records north from Vojvodina, in Slovakia in 2004 [31], the Czech Republic in 2005 [32], and Austria in 2012 [33] confirm our observation. Due to its exophilic and exophagic behaviour, *An. hyrcanus* has never been considered as the primary vector of malaria in Europe. Its spread to higher latitudes, combined with the changes in human behaviour (increased outdoor leisure activities, the mobility of humans, number of seasonal workers in the field, number of migrants in Europe), might elevate its vector capacity. The similar northern spread of population distribution range that was registered for *Anopheles maculipennis s.s.* in Russia [34], and *Culiseta longiareolata* in southern (in 2012; [35]) and northern (in 2013 [36]) Austria might well represent the tendency described with our model.

The latest illustration of similar changes is the finding of *Uranotaenia unguiculata*, a thermophilic mosquito species frequently occurring in the Mediterranean basin, in northern Germany, some 300-km north of the previous northern limit [37].

During the period 2001-2030 in which the spread and population growth of *An. hyrcanus* is expected, the intensity of UVR is likely to increase in the VPS (Fig 4a). Let us note, that the positive trends which are already present in observations might indicate that the findings supporting the negative influence of UVR and blue-light radiation (this radiation has a wavelength between approximately 380 nm and 500 nm; it has a very short wavelength, and so produces a higher amount of energy) on adult mosquitoes under laboratory conditions [38,39]. This experimental evidence does not mean unavoidably that the blue light radiation has significant influence on adult mosquitoes in field conditions, since they are able to actively escape over-exposure to radiation.

A positive association between WNV disease and temperature was already reported in Europe [11,40] where climate and landscape were critical predictors of WNV disease outbreaks [41]. Our focus was not on the number of human WNV cases, but the suitability of sites/microhabitats with different air temperatures for WNV circulation in mosquitoes, which may well correspond to a higher risk of transmission. We found that sites with higher T_{oa} and T_a were characterized with higher WNV mosquito incidence rate. Clustering

of cases with an incidence higher than one in six years coincided with an area of a significant grouping of mosquito, bird, horse and human cases in 2014 and 2015 (Petrić et al. [42]– Fig 5). This is in concurrence with Tran et al. [43] and Marcantonio et al. [41], who found that average summer temperatures are positively correlated with WNV human incidence. It seems that temperature in semi-urban areas dominates the other environmental factors influencing WNV circulation in nature (e.g. landscape suitability for reservoir host and mosquito vector, host availability, precipitation), as it is the primary factor affecting both mosquito vector abundance and virus replication. Prediction of a two-fold increase in virus incidence for each 0.5°C increase in T_{oa} indicates but does not necessarily mean, that the number of human cases could increase too. Therefore, our findings support the statement that climate change is likely to intensify the re-emergence of WNV in Europe [44].

Fig 5. Frequency of sampling of WNV infected mosquitoes (1 – 5 times, coloured numbers) during the period 2010-2016, superimposed over a cluster of mosquito, bird, horse and human WNV cases in (a) 2014 and (b) 2015 (modified after Petrić et al. [40]).

Melanoma incidence and UVR. According to World Health Organization (WHO) (1992) and many other authors [45,46], exposure to UVR radiation is considered to be a major etiological factor for all three forms of melanoma (i) basal cell carcinoma (BCC), (ii) squamous cell carcinoma (SCC) and (iii) malignant melanoma (MM). We found the correlation between MM and climate changes impact on UVR and also the number of HD. We see the impact as a modification of ambient UVR through influences on other variables such as clouds and aerosols. However, that impact might be more pronounced through the impact of changes in outdoor ambient temperature which will influence people's behaviour and increase the time they spend outdoors, i.e. exposure to both higher UVR and higher temperatures [13]. Experiments with animals clearly show that increased temperatures enhance UVR-induced melanoma compared to the room temperature. In an intriguing study, van der Leun [47] speculated that long-term elevation of temperature by 2°C, as a consequence of climate change, would increase the carcinogenic effects of UVR by 10%. Our results for

the UVR in the VPS are generally similar to the ones obtained by Malinović-Milićević et al. [48] and Malinović-Milićević and Radovanović [49], who reported the following changes: (1) the reduction of yearly averages for the total ozone of 3.44% and 3.21% and (2) increase in erythemal UVR dose of 6.9% and 9.7% for the periods 1990-1999 and 2000-2009, respectively.

According to Jovanović et al. [14], the incidence rate of MM cancer in VPS for the period 1985-2004 is higher than in central Serbia and is comparable with the majority of the central European countries as the highest melanoma incidence rate in the world [50]. However, most studies do not deal more quantitatively with the relationship between UVR doses and exposure during HD days and as it has been stated above, the cumulative exposure to sunlight is probably the most critical risk factor for MM and SCC cancers, while BCC is more associated with intensive short-term exposure [51]. Thus, the increasing trend in the number of melanoma incidence in the VPS for the period 1985-2004 (Fig 4d) can be ascribed to (1) the increase in the number of HD days for about 55% and (2) the increase in cumulative values of UVR doses for the period 1985-2030.

From a statistical point of view, the linear regression model for modeling the cumulative incidence of melanoma versus the difference of the cumulative UVR doses for hot and warm days (Fig 4d) is apparently acceptable. Parameters are statistically highly significant ($r = 0.971$ and $p < 0.001$) while analysis of residual distribution shows a good agreement with the normal distribution (*p* - *p* plot) as it is seen in Figure 6.

Fig 6. Residual distribution versus normal distribution (p-p plot) for regression in Fig 4d.

We hope that our results will indicate the importance of long-term monitoring/surveillance programs for providing crucial data to evidence the ongoing biological alteration triggered by climate change. Nonetheless, it is difficult to say how broadly our data represent the trends elsewhere. We believe that the specificity of the observations offers a unique window into the state of some of the planet's pressing threats to human health. Also, in the case where the humans are exposed to UVR, due to the nature of their work (the VPS is an exclusively agricultural area), it is necessary to (i) establish a broader network for UVR

measurements and warning centres and (ii) increase the awareness of the melanoma as a result of increased amount of UVR.

Acknowledgements

This paper was realized as a part of the project "Studying climate change and its influence on the environment: impacts, adaptation and mitigation" (43007) financed by the Ministry of Education and Science of the Republic of Serbia, within the framework of integrated and interdisciplinary research for the period 2011-2017. Historical data for mosquito vectors are the outputs of the projects supported by the Veterinary Directorate, Ministry of Agriculture and Environment Protection, Republic of Serbia, Provincial Secretariat for Science and Technological Development, AP Vojvodina; TR31084 MESRS. The works of Dušan Petrić and Mina Petrić were done under the frame of EurNegVec COST Action TD1303.

References

1. Costello A, Abbas M, Allen A, Ball S, Bell S, Bellamy R, et al. Managing the health effects of climate change: lancet and University College London Institute for Global Health Commission. The Lancet. 2009;373: 1693–1733.

2. Mihailović DT, Lalić B, Drešković N, Mimić G, Djurdjević V, Jančić M. Climate change effects on crop yields in Serbia and related shifts of Köppen climate zones under the SRES-A1B and SRES-A2. Int J Climatol. 2015;35: 3320–3334. doi:10.1002/joc.4209

3. King LJ, Anderson LR, Blackmore CG, Blackwell MJ, Lautner EA, Marcus LC, et al. Executive summary of the AVMA One Health Initiative Task Force report. J Am Vet Med Assoc. 2008;233: 259–261. doi:10.2460/javma.233.2.259

4. Health - European External Action Service [Internet]. [cited 29 Mar 2019]. Available: https://eeas.europa.eu/headquarters/headquarters-homepage/2348/health_ku

5. One World, One Health: OIE - World Organisation for Animal Health [Internet]. [cited 27 Mar 2019]. Available: http://www.oie.int/en/for-the-media/editorials/detail/article/one-world-one-health/

6. Kjellstrom T, McMichael AJ. Climate change threats to population health and well-being: the imperative of protective solutions that will last. Glob Health Action. 2013;6. doi:10.3402/gha.v6i0.20816

7. Zeller H, Lenglet A, Bortel WV. West Nile virus: the need to strengthen preparedness in Europe. Eurosurveillance. 2010;15: 19647. doi:10.2807/ese.15.34.19647-en

8. Weekly updates: 2018 West Nile fever transmission season [Internet]. [cited 27 Mar 2019]. Available: https://ecdc.europa.eu/en/west-nile-fever/surveillance-and-disease-data/disease-data-ecdc

9. Vakali A, Patsoula E, Spanakos G, Danis K, Vassalou E, Tegos N, et al. Malaria in Greece, 1975 to 2010. Euro Surveill Bull Eur Sur Mal Transm Eur Commun Dis Bull. 2012;17.

10. Wilson N, Lush D, Baker MG. Meteorological and climate change themes at the 2010 International Conference on Emerging Infectious Diseases. Euro Surveill Bull Eur Sur Mal Transm Eur Commun Dis Bull. 2010;15.

11. Calzolari M, Pautasso A, Montarsi F, Albieri A, Bellini R, Bonilauri P, et al. West Nile virus surveillance in 2013 via mosquito screening in northern Italy and the influence of weather on virus circulation. PLoS One. 2015;10: e0140915.

12. Apalla Z, Lallas A, Sotiriou E, Lazaridou E, Ioannides D. Epidemiological trends in skin cancer. Dermatol Pract Concept. 2017;7: 1.

13. Kerr JB, Seckmeyer G. Surface ultraviolet radiation: past and future Scientific Assessment of Ozone Depletion 2002. Geneva; 2003. Report No.: 47.

14. Jovanović M, M Bogdanović G, Mijatović Jovanović V, Jeremić P, Ac Nikolić E. Analysis of cutaneous melanoma in the province of Vojvodina. J BUON Off J Balk Union Oncol. 2009;14: 441–6.

15. Đurđević V, Rajković B. Development of the EBU-POM coupled regional climate model and results from climate change experiments. In: Mihailovic DT, Lalic B, editors. Advances in environmental modeling and measurements. New York, USA: Nova Science Publishers Inc; 2012. pp. 23–32.

16. Roeckner E, Bäuml G, Bonaventura L. The atmospheric general circulation model ECHAM5. Part I: Model description Report. Max Planck Institute for Meteorology; 2003 p. 127. Report No.: 349.

17. Jungclaus JH, Keenlyside N, Botzet M, Haak H, Luo J-J, Latif M, et al. Ocean Circulation and Tropical Variability in the Coupled Model ECHAM5/MPI-OM. J Clim. 2006;19: 3952–3972. doi:10.1175/JCLI3827.1

18. Malinovic-Milicevic S, Mihailovic DT, Lalic B, Dreskovic N. Thermal environment and UV-B radiation indices in the Vojvodina region, Serbia. Clim Res. 2013;57: 111–121. doi:10.3354/cr01163

19. Srdić Ž, Zgomba M, Petrić D. Les moustiques (Dip.Culicidae) et la demoustication en Vojvodina, Yugoslavia. IV Congress sur la protection de la santehumaineet des cultures on mileu tropical. Marseille, France; 1986. pp. 489–495.

20. Petrić D. Seasonal and daily activity of adult mosquitoes (Diptera, Culicidae) in Vojvodina Province. University of Novi Sad. 1989.

21. Petrić D, Zgomba M, Bellini R, Becker N. Surveillance of Mosquito Populations: A Key Element to Understanding the Spread of Invasive Vector Species and Vector-Borne Diseases in Europe. In: Mihailovic DT, editor. Essays of Fundamental and Applied Environmental Topics. New York, USA: Nova Science Publishers; 2012. pp. 193–224.

22. Becker N, Petrić D, Boase C, Lane J, Zgomba M, Dahl C, et al. Mosquitoes and their control. Springer; 2010.

23. Petrović T, Šekler M, Petrić D, Lazić S, Debeljak Z, Vidanović D, et al. Methodology and results of integrated WNV surveillance programmes in Serbia. PLOS ONE. 2018;13: e0195439. doi:10.1371/journal.pone.0195439

24. Mihailović DT, Mimić G, Arsenić I. Climate Predictions: The Chaos and Complexity in Climate Models. In: Advances in Meteorology [Internet]. 2014 [cited 27 Mar 2019]. doi:10.1155/2014/878249

25. Richman JS, Moorman JR. Physiological time-series analysis using approximate entropy and sample entropy. Am J Physiol Heart Circ Physiol. 2000;278: H2039-2049. doi:10.1152/ajpheart.2000.278.6.H2039

26. R Core Team. R: A Language and Environment for Statistical Computing [Internet]. Vienna: R Foundation for Statistical Computing; 2016. Available: http://www.R-project.org/

27. HS Global Inc. Eviews [Internet]. CA, USA: HS Global Inc.; 2016. Available: http://www.eviews.com

28. TIBCO Software Inc. Statistica [Internet]. CA, USA: TIBCO Software Inc.; Available: https://docs.tibco.com/products/tibco-statistica-13-3-0

29. Hallmann CA, Sorg M, Jongejans E, Siepel H, Hofland N, Schwan H, et al. More than 75 percent decline over 27 years in total flying insect biomass in protected areas. PloS One. 2017;12: e0185809.

30. Adamović Ž. Distribution and abundance of anopheline mosquitoes (Diptera, Culicidae) in Vojvodina, Serbia. Acta Entomol Jugosl. 1979;15: 82.

31. Halgoš J, Benková I. First record of Anopheles hyrcanus (Diptera: Culicidae) from Slovakia. Biológia. 2004;59: 68.

32. Votýpka J, Šeblová V, Rádrová J. Spread of the West Nile virus vector Culex modestus and the potential malaria vector Anopheles hyrcanus in central Europe. J Vector Ecol. 2008;33: 269–277. doi:10.3376/1081-1710-33.2.269

33. Lebl K, Brugger K, Rubel F. Predicting Culex pipiens/restuans population dynamics by interval lagged weather data. Parasit Vectors. 2013;6: 129. doi:10.1186/1756-3305-6-129

34. Novikov YM, Vaulin OV. Expansion of Anopheles maculipennis s.s. (Diptera: Culicidae) to northeastern Europe and northwestern Asia: Causes and Consequences. Parasit Vectors. 2014;7: 389. doi:10.1186/1756-3305-7-389

35. Seidel B, Nowotny N, Duh D, Indra A, Hufnagl P, Allerberger F. First records of the thermophilic mosquito Culiseta longiareolata (Macquart, 1838) in Austria, 2012, and in Slovenia, 2013. J Eur Mosq Control Assoc. 2013;31: 17–20.

36. Zittra C, Waringer J, Werblow A, Melaun C, Fuehrer H-P. Reconfirmation of Culiseta (Allotheobaldia) longiareolata (Macquart 1838) (Diptera: Culicidae) in Austria. The first sequence-

confirmed findings in northeastern Austria. Acta ZooBot Austria. 2014;150: 17–24.

37. Tippelt L, Walther D, Kampen H. The thermophilic mosquito species Uranotaenia unguiculata Edwards, 1913 (Diptera: Culicidae) moves north in Germany. Parasitol Res. 2017;116: 3437–3440. doi:10.1007/s00436-017-5652-2

38. Riordan DF. Effects of ultraviolet radiation on adults of Aedes aegypti (L.) (Diptera : Culicidae). Mosq News. 1969;29. Available: https://www.cabdirect.org/cabdirect/abstract/19711000053

39. Hori M, Shibuya K, Sato M, Saito Y. Lethal effects of short-wavelength visible light on insects. Sci Rep. 2014;4: 7383. doi:10.1038/srep07383

40. Paz S, Malkinson D, Green MS, Tsioni G, Papa A, Danis K, et al. Permissive Summer Temperatures of the 2010 European West Nile Fever Upsurge. PLOS ONE. 2013;8: e56398. doi:10.1371/journal.pone.0056398

41. Marcantonio M, Rizzoli A, Metz M, Rosà R, Marini G, Chadwick E, et al. Identifying the Environmental Conditions Favouring West Nile Virus Outbreaks in Europe. Gourbiere S, editor. PLOS ONE. 2015;10: e0121158. doi:10.1371/journal.pone.0121158

42. Petrić D, Petrović T, Hrnjaković Cvjetković I, Zgomba M, Milošević V, Lazić G, et al. West Nile virus “circulation” in Vojvodina, Serbia: Mosquito, bird, horse and human surveillance. Mol Cell Probes. 2017;31: 28–36. doi:10.1016/j.mcp.2016.10.011

43. Tran A, Sudre B, Paz S, Rossi M, Desbrosse A, Chevalier V, et al. Environmental predictors of West Nile fever risk in Europe. Int J Health Geogr. 2014;13: 26. doi:10.1186/1476-072X-13-26

44. Paz S. Climate change impacts on West Nile virus transmission in a global context. Philos Trans R Soc B Biol Sci. 2015;370: 20130561–20130561. doi:10.1098/rstb.2013.0561

45. Narayanan DL, Saladi RN, Fox JL. Ultraviolet radiation and skin cancer. Int J Dermatol. 2010;49: 978–986. doi:10.1111/j.1365-4632.2010.04474.x

46. Lacy K, Alwan W. Skin cancer. Medicine (Baltimore). 2013;41: 402–405. doi:10.1016/j.mpmed.2013.04.008

47. Leun JC van der. Uv-Carcinogenesis. Photochem Photobiol. 1984;39: 861–868. doi:10.1111/j.1751-1097.1984.tb08872.x

48. Malinovic-Milicevic S, Mihailovic DT, Radovanovic MM. Reconstruction of the erythemal UV radiation data in Novi Sad (Serbia) using the NEOPLANTA parametric model. Theor Appl Climatol. 2015;121: 131–138. doi:10.1007/s00704-014-1223-y

49. Malinović-Milićević S and Radovanović M 2016 UV radiation and heat wives in Vojvodina (Book 87) (Belgrade: Serbian Academy of Sciences and Arts, Institute of Geography „Jovan Cvijić”) p ISBN.

50. de Vries E, Tyczynski J, Parkin MD. Cutaneous malignant melanoma in Europe. Eur Netw Cancer Regist Int Agency Res Cancer. 2003;ENCR Cancer Fact Sheets no.4 1-4.

51. Demers AA, Nugent Z, Mihalcioiu C, Wiseman MC, Kliewer EV. Trends of nonmelanoma skin

cancer from 1960 through 2000 in a Canadian population. J Am Acad Dermatol. 2005;53: 320–328.

Supporting information

S1 Table. Overview of dry-ice trap samples sizes. For each year, the number of locations sampled, the number of location re-sampled, and total number of samples are presented. Exposure time at the trap locations was similar (14 ± 2h).

S2 Table. Number of the total trap nights, positive trap nights and *Anopheles hyrcanus* specimens sampled at 54 selected sites in the Vojvodina Province, Serbia during the years 1985-86, 2004-5 and 2014-15.

S3 Table. Frequency of sampling of WNV infected mosquitoes (1 – 5 times) in the Vojvodina Province, Serbia, during the period 2010-2016.

Figure 1

Click here to download Figure Fig1.tiff

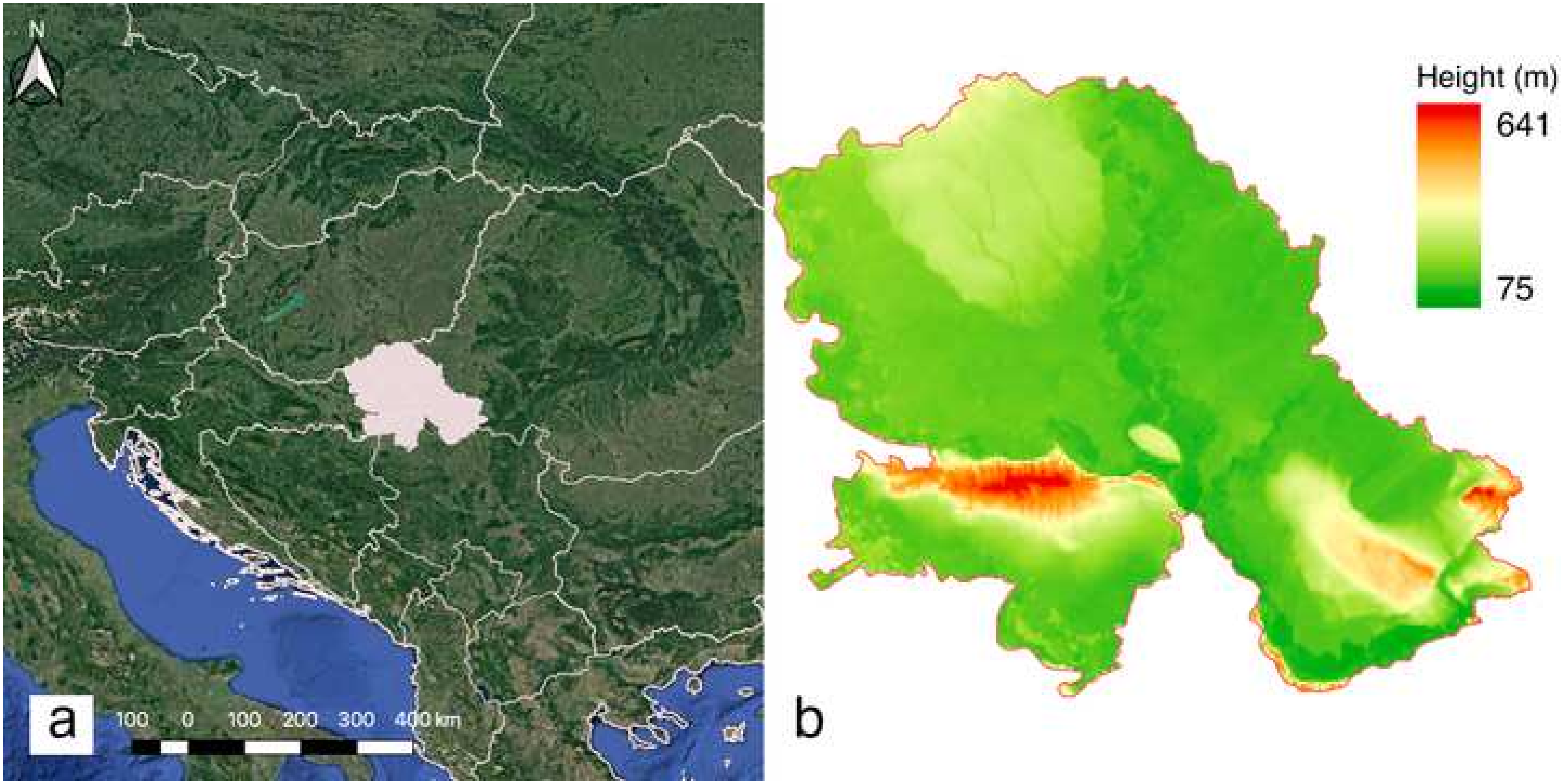

Figure 2

Click here to download Figure fig2.tiff

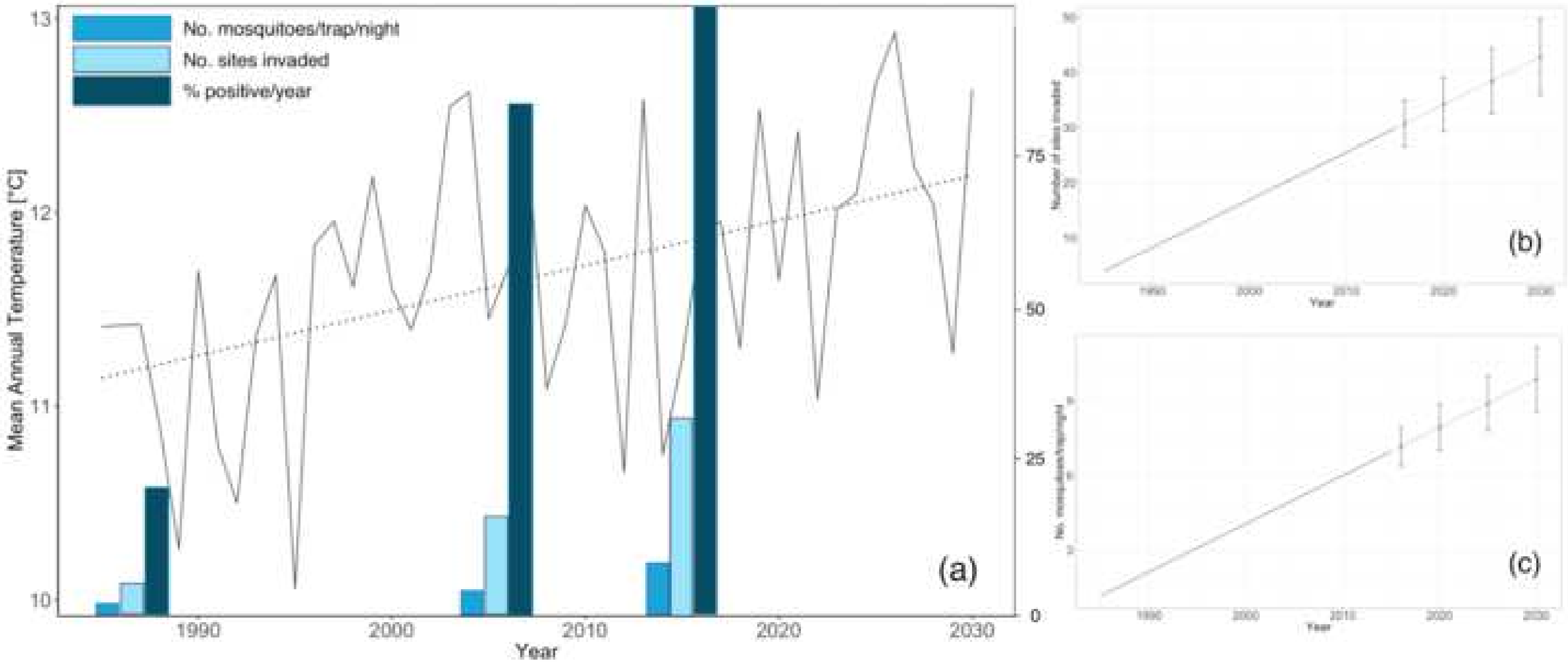

Figure 3

Click here to download Figure Fig3.tiff

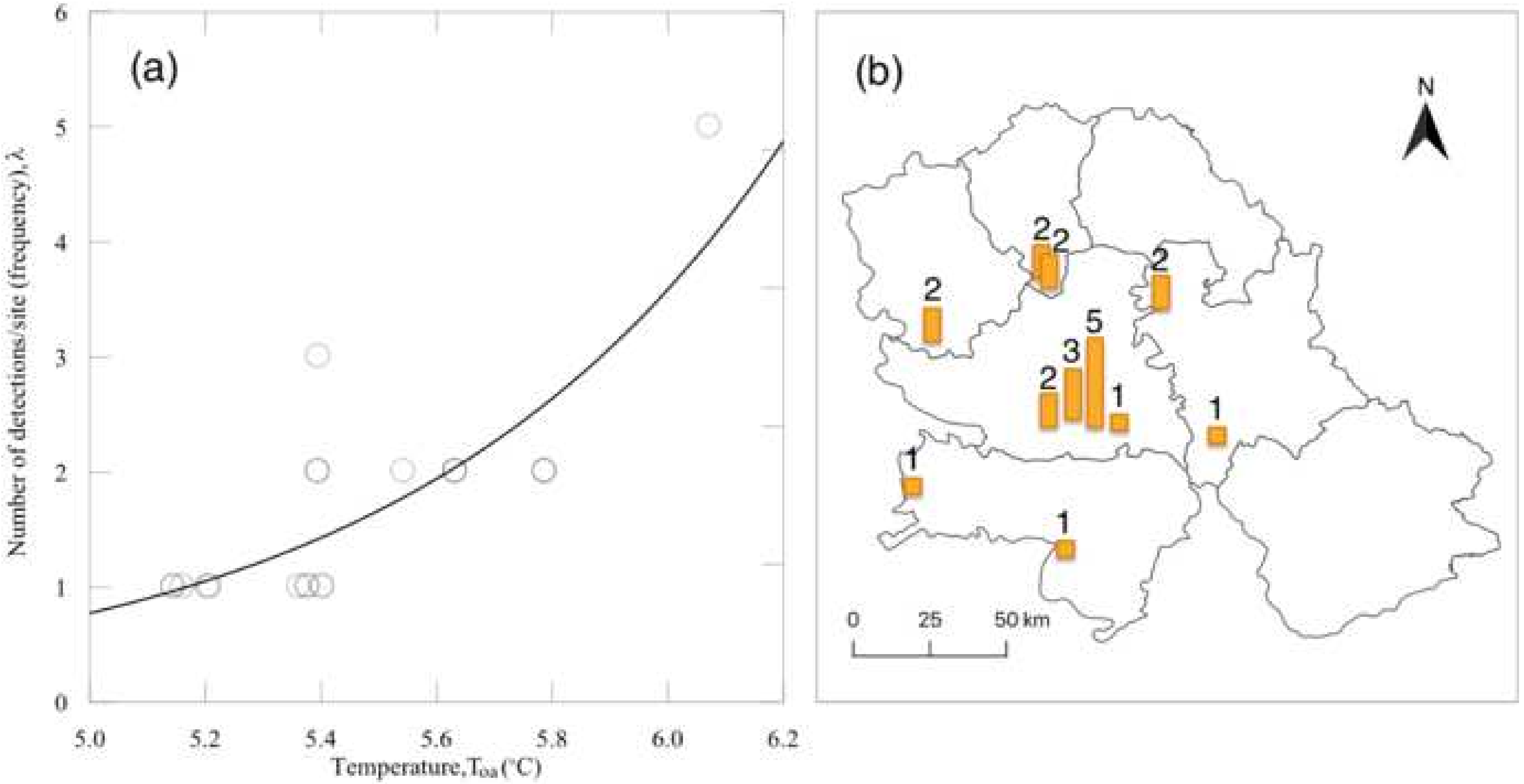

Figure 4

Click here to download Figure Fig4.tiff

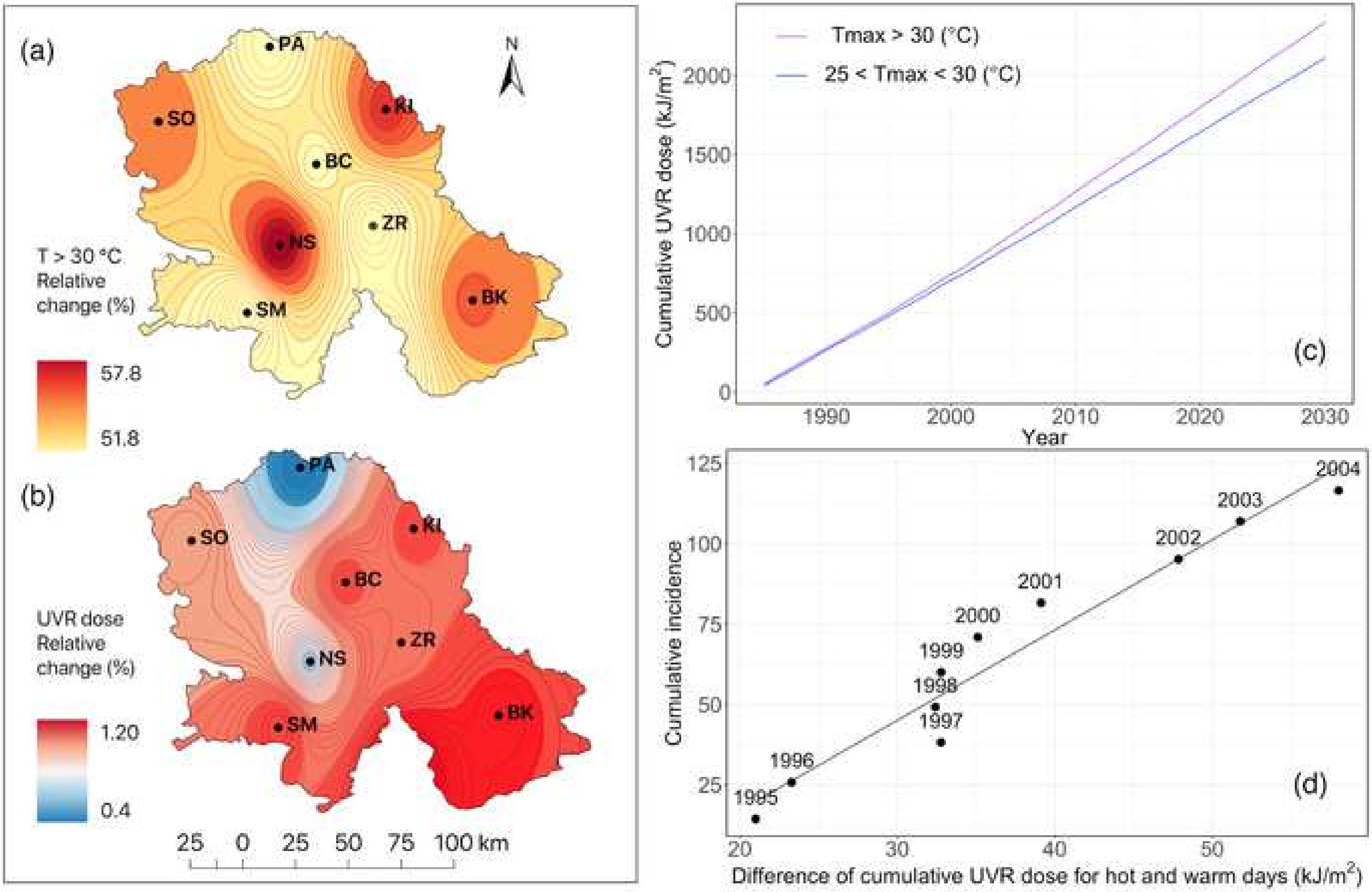

Figure 5

Click here to download Figure Fig5.tiff

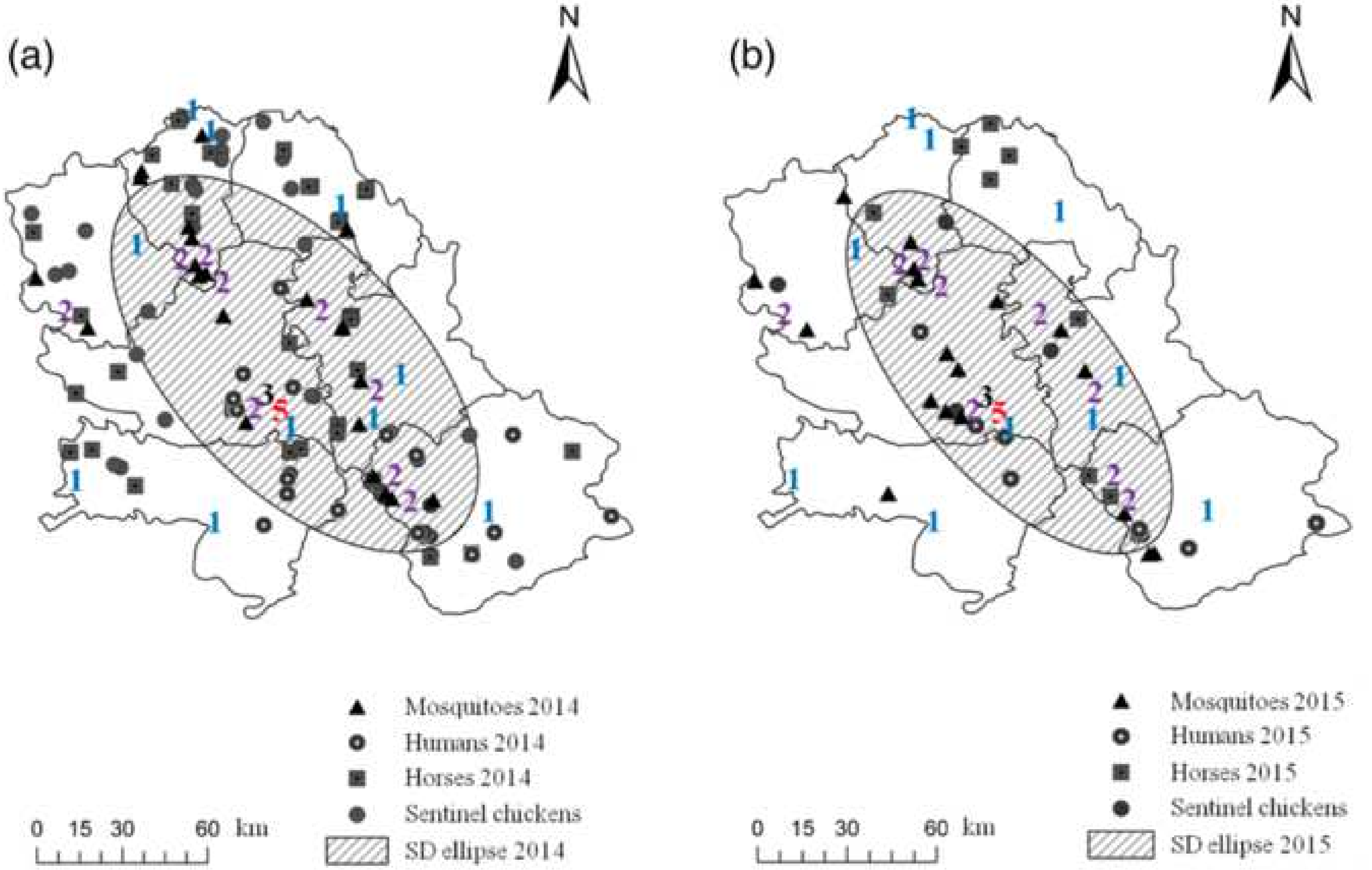

Figure 6

Click here to download Figure Fig6.tiff

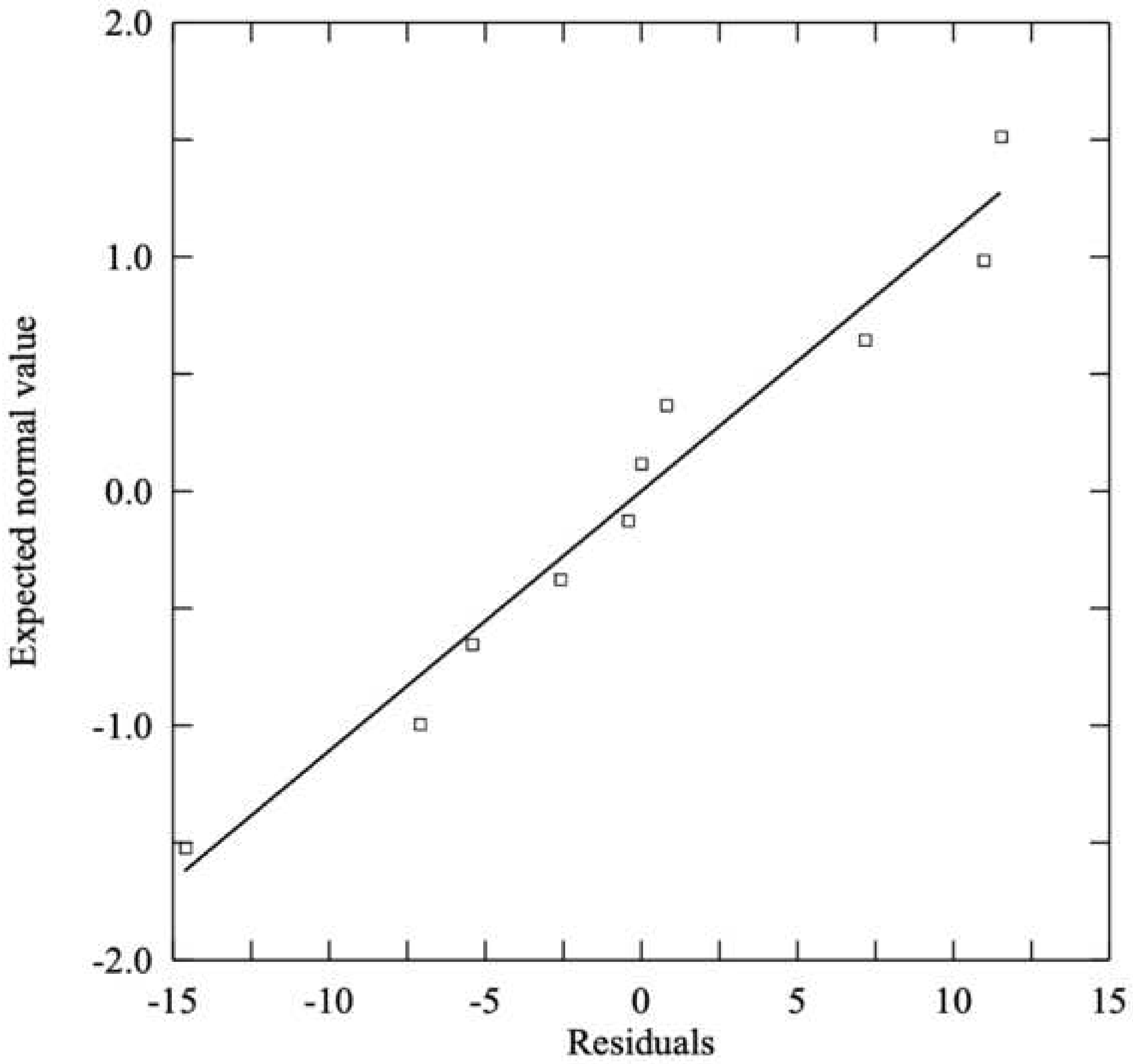

Supporting Information 1

Click here to access/download

Supporting Information

S1_Table.xlsx

Supporting Information 2

Click here to access/download
Supporting Information
S2_Table.xlsx

Click here to access/download
Supporting Information
S3_Table.docx
