# Supplementary material for: Assessment of climate change impact on the malaria vector Anopheles hyrcanus, West Nile disease, and incidence of melanoma in the Vojvodina Province (Serbia) using data from a regional climate model
Source: PLoS One. 2020 Jan 15;15(1):e0227679. doi: 10.1371/journal.pone.0227679 (PMC6961917; doi:10.1371/journal.pone.0227679)
Supplement: Attachment — Submitted filename: Mihailovic2019_Response_to_Reviewers.pdf [file pone.0227679.s006.pdf]

Journal: PLOS ONE

Collection: Urban Ecosystems

Manuscript#: PONE-D-19-16900

Title of paper: Assessment of climate change impact on the malaria vector *Anopheles hyrcanus*, West Nile disease, and incidence of melanoma in the Vojvodina Province (Serbia) using data from a regional climate model

Dragutin T. Mihailović[1], Dušan Petrić[2*], Tamaš Petrović[3], Ivana Hrnjaković-Cvjetković[4,5], Vladimir Djurdjevic[6], Emilija Nikolić-Đorić[7], Ilija Arsenić[1], Mina Petrić[8,9,10*], Gordan Mimić[11], Aleksandra Ignjatović-Ćupina[2]

Dear Dr Samy,

We are pleased to submit the revised version of "Assessment of climate change impact on the malaria vector *Anopheles hyrcanus*, West Nile disease, and incidence of melanoma in the Vojvodina Province (Serbia) using data from a regional climate model" (#PONE-D-19-16900). We appreciate the time and efforts by the editor and advisors in reviewing the manuscript. Please find below detailed responses to the reviewers, whom we thank for their careful consideration of the manuscript. We also reviewed the manuscript for any additional errors and made small changes that are tracked in the attached document ("Revised Manuscript with Track Changes").

Line numbers for the corrected text are given in red according to the enumeration in the file "Revised Manuscript with Track Changes".

Reviewer #1: Comments on the manuscript PONE-D-19-16900

Title: Assessment of climate change impact on malaria vectors, West Nile disease, and incidence of melanoma in the Vojvodina Province (Serbia) using data from a regional climate model. Authors: Dragutin T. Mihailović, Dušan Petrić, Tamaš Petrović, Ivana Hrnjaković Cvjetković, Vladimir Đurđević, Emilija Nikolić Đorić, Ilija Arsenić, Mina Petrić, Gordan Mimić

The work presented in paper Mihailović et al. is interesting. The objective of the authors was to compare data from the mosquito field collections and medical studies with regional 29 climate model projections to examine the impact of climate change on the circulation of West Nile virus (WNV), the spreading of the malaria vector Anopheles hyrcanus and the incidence of melanoma. The comparison was done with the coupled regional Eta Belgrade University and Princeton Ocean Model for the period 1961-2015 using the A1B scenario, and the expected changes up to 2030. Overall, significant correlation was found between the frequency of WNV in Culex pipiens and the overwintering temperature averages and seasonal relative humidity at the sampling sites. Correlation was also found between the spreading and relative abundance of Anopheles hyrcanus and the trend of the mean annual temperature. There was also an increase in melanoma incidence.

Minor comments to authors

Title

Authors wrote “malaria vectors” but the only presented data on only one vector Anopheles hyrcanus

Corrected according to the suggestion.

Abstract

L32: ……………and 10-years. Delete the hyphen

L36: *Corrected according to the suggestion.*

L37: ……………Culex. pipiens. Delete the dot

L44: *We abbreviated the genus name to Cx. because name was spelled in the previous sentence.*

Introduction

L49-53: Are the authors referring to themselves when they stated, “The authors (……….), have been working together…….”?

Yes, we tried to make it clear with correction below.

L56: **Corrected to:** “The authors of the manuscript (……….), have been working together…….”

L72: Authors should write, “Climate change……..” instead of “The climate change……..”

L86: *Corrected according to the suggestion.*

L75: Author should write, "Melanoma mortality…………..within the period 1985-2004

L89-91: *Corrected according to the suggestion.*

L78: Authors should define the acronym ENCR, as this is used here for the first time.

L83: … using ENCR data

L93-94: **Changed to:**

… using European Network of Cancer Registries (ENCR) data

L81-82: Authors should use past tense in the sentence "……we compared considerable of previously……"

L97: *Corrected according to the suggestion.*

L54-55: This sentence is not clear for me. I suggest this: "In this paper, we analysed observed data collected over a period of 31 years……"

L61: *Corrected according to the suggestion, "collected" erased because of tautology.*

Materials and Methods
L96: Authors should define SRES-A1B scenario for the first time.

The SRES-A1B scenario is defined in the text, and central differences to RCP explained. Due to this, authors think that selection of scenario, to some extent, is irrelevant for the presented results.

L96**:** … and the period 2001-2030 using the SRES-A1B scenario.

L113-122 **Corrected to:**

… and the period 2001-2030 according to the A1B scenario defined in Special Report on Emissions Scenarios (SRES) (Nakićenović and Swart, 2000), from now on SRES-A1B. SRES scenarios, which defined future global greenhouse gases emissions, were extensively used in the Intergovernmental Panel on Climate Change (IPCC) Third, and Fourth Assessment Reports. The main storyline behind the A1B scenario is rapid economic growth, followed by a significant increase in greenhouse gases concentrations in the future. In the Fifth Assessment Report (AR5), the Representative Concentration Pathway (RCP) is introduced, which are possible future

concentration pathways without any storyline behind them. Comparing SRES-A1B and RCPs in terms of the greenhouse gases concentrations, at the end of this century SRES-A1B is the closest to RCP6.0, but for the time horizon used in this study, up to 2030, the difference between any SRES or RCPs are relatively small.

New references included:

Summary for Policy makers. In: Nakićenović N, Swart R, editors. Special Report on Emissions Scenarios. Cambridge: Cambridge University Press - Published for the Intergovernmental Panel on Climate Change. 2000. pp. 1-21

L115: Authors used only one formula, the subtitle should be in singular

L141: *Corrected according to the suggestion.*

Results

- Authors should specify the exact p-values instead of writing $p<0.05$ or $p>0.05$

L260-281: *Corrected according to the suggestion. The section was updated to include the exact p-values.*

L207: "The Poisson regression model for the dependence of a number of detections per site (frequency-λ)…………………………is highly significant". Authors stated it was highly significant, but from my perspective, $p<0.05$ is not a specific indication of high significance. Could the give the exact value of p?

L282-283: *Corrected according to the suggestion. Exact p value is not below 0,01 (it is 0.01393) which is considered as high significance by many authors, so we erased the word "highly".*

- Fig2b and 2c are fuzzy

Thank you for your comment. The figures were reformatted to higher resolution according to PACE.

- Fig4. Colors of fig4c are confusing

Figures 4c has been adapted to have more contrasting colors.

It will be more interesting if the authors used only vector-borne diseases data in this paper.

N.B: Other comments are incorporated in the manuscript

Authors appreciate very much the effort invested in the improvement of the manuscript quality. All suggestions are incorporated into the revised version except one concerning the spelling of NUTS. Nomenclature of territorial units for statistics is originally abbreviated NUTS from the French version (Nomenclature des Unités territoriales statistiques).

Reviewer #2:

Authors are presenting an interesting paper regarding the effects of climate change in Northern Serbia considering three independent measures: The spread of Anopheles hyrcanus, the presence of West Nile Virus in Culex pipiens, and the incidence of melanoma cases. The paper is interesting, however, discussion should be improved specially on the uncertainty of future predictions since they are using just one climatic model. Further, their results should be stated more carefully since their model rely on assumptions (e.g., manually selected variables) which are also not clearly stated.

Discussion is corrected according to the reviewer's suggestion:

L345-352: New text added:

The temperature trend over the period of observations used in this study and for the future time horizon following A1B scenario obtained with the EBU-POM regional climate model is within the multi-model ensemble (MME) spread of regional climate models with similar configuration used in the ENSEMBLES project (van der Linden and Mitchell, 2009). For the period 2001-2030 the temperature change for the region of interest in the EBU-POM integration is 0.75 °C concerning the period 1961-1990 and for the same period ENSEMBLES MME spread range is 0.5-1.5 °C (MEP, 2017). Following this finding, other results presented in this paper that relay on temperature change, can be seen as an estimate that will be within uncertainty related to the future temperature projection.

New references included:

van der Linden P and Mitchell JFB, editors. ENSEMBLES Climate Change and its Impacts. Summary of research and results from the ENSEMBLES project. Exeter: Met Office Hadley Centre;. 2009.:

Ministry of Environmental Protection of the Republic of Serbia. Second National Communication of the Republic of Serbia under the United Nations Framework Convention on Climate Change [Internet]. Belgrade: The Ministry; 2017 Aug. 162p [cited 2019 Sep 10]. Available from: http://www.klimatskepromene.rs/wp-content/uploads/2017/09/SNC_eng.pdf

Major comments:

The paper is showing results in the order: malaria vector, WNV, and melanoma. I suggest following the same order in the abstract.

Changed as suggested.

Authors are using one of the SRES future climatic scenarios; currently the standard for future climate studies are the RCP scenarios. Authors should describe the nature of the SRES-A1B scenario, which is not mentioned in any part of the study. Further, authors should explicitly discuss uncertainty on their predictions since they are not using other scenarios or other climatic models.

Authors addressed this comment in the text corrected. Please check response to the L113-122 and L 345-352 above.

Lines 176-180. There is no discussion or results regarding these sentences. Was the comparison between EBU-POM model and the Republic Hydrometeorological Service of Serbia perfect? What is the implication of this approach on the overall paper?

This is a valuable comment since the information measure(s) is(are) a good indicator of the reliability of model outputs and thus on the overall paper. The increasing complexity of climate models is a growing concern in the modelling community. However, because we invested a serious effort to make our models more "realistic", we included more parameters and processes. With increasing model complexity, we are less able to manage and understand the model behaviour. Thus, from a user's perspective, the following question is entirely natural: "How complex model (EBU-POM model in our case) do I need to use to study this problem (assessment of climate change impact on malaria vectors, West Nile disease, and incidence of melanoma in the VPS) with this data set (temperature and/or precipitation)?". In the revised version, we inserted the additional text.

L229-249: New text added:

We considered the papers by Mihailović et al. [2,24] in which Kolmogorov complexity measures (Kolmogorov complexity (KC), Kolmogorov complexity spectrum KC spectrum) and the highest value of the KC spectrum (KCM)) and sample entropy (SE) [25] were used to quantify the regularity and complexity of air temperature and precipitation time series, obtained by the EBU-POM model, representing both deterministic chaos and stochastic processes. We considered the complexity of the EBU-POM model using the observed and modelled time series of temperature and precipitation. We computed the KC spectrum, KC, KCM and SE values for temperature and precipitation. The calculations were performed for the entire time interval

1961–1990: (1) on a daily basis with a size of N =10958 samples for temperature and (2) on a monthly basis with a size N =360 for the precipitation. The simulated time series of temperature and precipitation were obtained by the EBU-POM model for the given period. The observed time series of temperature and precipitations for two stations: Sombor (SO) (88 m a.s.l.) and Novi Sad (NS) (84 m a.s.l.) in the considered area, were taken from daily meteorological reports of the Republic Hydrometeorological Service of Serbia. For both sites, the modelled complexity is lower than the observed one, but with the reliability which is in the interval values allowed by the information measures (KC, KCM, and SE) (Krzic et al. 2011, Dell' Aquila et al. 2016, Cavicchia et al. 2016). These findings mean that the models with a KC (and KCM) complexity lower than the measured time series complexity cannot always reconstruct some of the structures contained in the observed data. However, it does not mean that outputs from EBU-POM model do not correctly simulate climate elements since both sites values indicate the absence of stochastic influences, providing reliable projections of the climate elements.

New references included:

Krzic A, Tosic I, Djurdjevic V, Veljovic K, Rajkovic B. Changes in some indices over Serbia according to the SRES A1B and A2 scenarios. Climate Research. 2011;49: 73-86.

Cavicchia L, Scoccimarro E, Gualdi S, Marson P, Ahrens B, Berthou S, et al. Mediterranean extreme precipitation: a multi-model assessment. Clim. Dyn. 2016. doi: 10.1007/s00382-016-3245-x

Dell' Aquila A, Mariotti A, Bastin S, Calmanti S, Cavicchia L, Deque M, et al. Evaluation of simulated decadal variations over the Euro-Mediterranean region from ENSEMBLES to Med-CORDEX. Clim. Dyn. 2016. doi:10.1007/s00382-016-3143-2

Line 277-280: There is no evidence in this paper supporting this affirmation since the variables analyzed corresponded to three temperature related variables and just one considering humidity. Moreover, results were never compared statistically; modify accordingly.

Corrections made as suggested. The sentence "It seems that temperature in semi-urban areas dominates the other environmental factors influencing WNV circulation in nature (e.g. landscape suitability for reservoir host and mosquito vector, host availability, precipitation), as it is the primary factor affecting both mosquito vector abundance and virus replication." *now reads as:*

L400-404: Corrected to: It seems that temperature in semi-urban areas is an essential environmental factor influencing WNV circulation (landscape suitability for reservoir host and mosquito vector, host availability, and precipitation/water availability are somewhat similar in

investigated semi-urban areas of VPS), as it affects both mosquito vector abundance and virus replication.

Figure 2: Expand the acronym CRCM. Also, double check the legend, which is describing red and green colors but the figure is only showing different shades of blue.

Corrections made as suggested.

L173-179: Fig 2. (a) The regional climate model EBU-POM projection of the mean annual air temperature (T_a) for the period 1985 - 2030 and: i) number of specimens sampled in one trap during single sampling period (blue columns); ii) the number of sites invaded by *An. hyrcanus* (light blue columns); and iii) relative number of positive samplings per year (dark blue columns), (b) projected increase in the number of sites invaded by *An. hyrcanus* (the period 2001-2030 ±S.E.), and (c) projected increase in the number of the specimens sampled in one trap during single sampling period (2001 - 2030 ±S.E.).

Figure 4: Add WD and HD to the corresponding legend of the graphic. Is there a Croatian sentence in the legend? Please describe how the melanoma incidence was calculated, is the y axis showing incidence or number of cases? Cumulative incidence is known to over-represent trends (see reference: Vandenbroucke & Pearce, 2012, doi: 10.1093/ije/dys142), try to use incidence rate instead.

This is a keystone issue in this field of epidemiology. However, it is still under a broad umbrella of discussion. In particularly mentioned reference (Vandenbroucke & Pearce, 2012) the Authors comprehensively considered the place of incidence rates in dynamic populations as well as the cumulative incidence (risk or portion) from an epistemological point of view and also giving very illustrative (educational) examples. Many authors were arguing with some ideas explicated in this paper, also considering some examples. We agree with V&P ideas, but we did not find the place where they explicitly say that it would always be using the incidence rate instead of cumulative (the question of overestimation). To our understanding, they left the space for a situation when the use of cumulative incidence gives acceptable results. For example, it is partly seen in the paper by Wu et al. (2014). There is another moment why we used cumulative incidence. It is well-known that there is a high correlation between sun exposure (and received cumulated doses of the UV radiation) and melanoma. If that doses (or any climate element) on a daily basis are used from regional climate models, they cannot be directly correlated with daily or monthly measured or calculated biological quantities. The reason for that is the fact that from regional climate models, we can estimate just the trend of the considered physical quantity

(in our case -UV doses through their cumulative values). Correspondingly it is correlated with cumulative incidence. Having said that, after the end of the statement in Line 336, we inserted the following text.

The legend in Fig. 4(c) and y-axis in Fig 4 (d) are changed as suggested.

The M&M - Melanoma incidence and UVR was amended by the following text:

L216-221: New text added:

In the analysis we have used two indicators: (i) melanoma incidence rate that is a measure of the number of new cases ("incidence") per unit of time ("rate") and (ii) cumulative incidence (“incidence proportion” that measures the number of new cases per person in the population over a defined period of time – often called risk or proportion). Melanoma incidence rate (per 100,000 people) for ten years 1995 - 2004 was based on the data obtained from the paper by Jovanović et al. [7]. From these data, we calculated the cumulative incidence.

The discussion was also amended by the following text:

L437-441: New text added:

In a cohort study, Wu et al. (2014) considered the impact of long-term UV radiation flux on skin cancer risk. Comparing with participants in the lowest quintile of cumulative UV radiation flux in adulthood, they found that participants in the highest quintile had multivariable-adjusted risks (cumulative incidence). According to Vandenbroucke and Pearce (2012), some studies where cumulative incidence is used can over-represent the trends.

New references included:

Wu S, Han J, Laden F, Qureshi AA. Long-term ultraviolet flux, other potential risk factors, and skin cancer risk: a cohort study. Cancer epidemiology, biomarkers and prevention: a publication of the American Association for Cancer Research, cosponsored by the American Society of Preventive Oncology. 2014;*23*(6):1080–1089.

Vandenbroucke JP, Pearce N. Incidence rates in dynamic populations. Int J Epidemiol. 2012;41(5):1472–1479.

In table S3 consider adding the number of mosquito samples per site.

Number of samples added in the table.

Figure 6 can be replaced with the statistics of such graphic for readers' interpretation.

L442-446: *Corrections made as suggested. Figure 6 deleted, the paragraph now reads as:*

From a statistical point of view, the linear regression model for modelling the cumulative incidence of melanoma versus the difference of the cumulative UVR doses for hot and warm days (Fig 4d) is acceptable. Parameters are statistically highly significant (r = 0.9712 and p = 0.000003) while analysis of residual distribution shows a good agreement with the normal distribution (Shapiro-Wilk test, W = 0.9608, p = 0.7952).

Authors are justifying the paper under the 'One Health' concept, however they are not discussing the idea further. I would like discussing explicitly the benefits of putting together a set of multidisciplinary specialists to the development of the manuscript and how this contribution is part of the one health concept.

The discussion was amended by the following text:

L322-344: New text added:

Despite globalisation trends, researchers have become "closed" in their ever-smaller communication circles which are not limited by state or national borders but by the professional language and way of thinking. Thus, by the end of the 20th century, the scientific community has been faced with problems in communication within its confines. One of the principal reasons why vector-borne diseases (VBD) are so difficult to predict, is the complex interaction of multiple factors (vector, host, pathogen, environment including short-term weather patterns and long-term climate change) in space and time (Moore 2008, Zimmerman 2014). Only groups from multiple sectors that communicate and work together on specific aspects of VBD systems will be able to answer the most exciting and pressing problems in the field (Moore 2008). Authors of this paper started collaboration in 2003 comparing the climates of the foci of WNV circulation in USA (California Central Valley) and Europe (Bucharest area) with VPS. As compared climates showed quite similar patterns, colleagues from public health and veterinary joined the initial group of meteorologists and medical entomologists. With the idea to better draw upon the resources and insights of the various sectors we designed and implemented research and programmes to achieve better outcomes in the control of zoonoses (diseases that can spread between animals and humans, e.g., WNV disease). This led us to the following achievements: (i) the first detection of WNV in horses in Serbia in 2009 (Lupulović 2011); (ii) the first detection of WNV in mosquitoes in Serbia in 2010 (26); (iii) the first detection of WNV in wild birds in Serbia in 2012 (Petrović 2013); (iv) development and

implementation of the national programme of WNV surveillance in mosquito, bird and horse population [8], combined with human surveillance in VPS from 2014; (v) increased visibility to ECDC, EFSA and WHO; (vi) the first detection of imported dengue human case in Serbia in 2016 (Petrović 2016); and (vii) development and implementation of "One Health" programme in VPS from 2018. We are quite sure that much less would have been achieved without multidisciplinary communication and collaboration initiated in 2003, and this paper would not have been compiled.

New references included:

Moore CG. Interdisciplinary research in the ecology of vector-borne diseases: Opportunities and needs. Journal of Vector Ecology 2008;33(2):218–224.

Zimmerman B. Engaging with Complexity: Thrive! A Plan for a Healthier Nova Scotia. 2014; [e-print] Available from: https://thrive.novascotia.ca/sites/default/files/Thrive-Summit-2014-Brenda-Zimmerman-En.pdf.

Petrić D, Hrnjaković-Cvjetković I, Radovanov J, Cvjetković D, Jerant-Patić V, Milošević V, et al. West Nile virus surveillance in humans and mosquitoes and detection of cell fusing agent virus in Vojvodina Province (Serbia). HealthMED 2012;6(2):462–68.

Lupulović D, Martin-Acebes MA, Lazić S, Alonso-Padilla J, Blazquez AB, Escribano-Romero E, et al. First serological evidence of West Nile virus activity in horses in Serbia. Vector Borne Zoonotic Dis. 2011;11(9):1303–5.

Petrović T, Blazquez AB, Lupulović D, Lazić G, Escribano-Romero E, Fabijan D, et al. Monitoring West Nile virus (WNV) infection in wild birds in Serbia during 2012: First isolation and characterisation of WNV strains from Serbia. Eurosurveillance. 2013;18(44):1–8.

Petrović V, Turkulov V, Ilić S, Milošević V, Petrović M, Petrić D, et al. First report of imported case of dengue fever in Republic of Serbia. Vol. 14, Travel Medicine and Infectious Disease. 2016. p. 60–1

Minor comments:

Please use Oxford comma across the manuscript: e.g., Line 30: 'the malaria vector, and the incidence of melanoma'.

Corrections made as suggested.

Line 28: Authors never discuss problems related with animal health, thus, I suggest avoiding this kind of affirmations (e.g., line 81).

The reviewer is right, we did not, but we think it is vital to mention animals because WNV is the important zoonotic diseases. Therefore, we would like to include new paragraphs in Introduction and Discussion.

The introduction was amended by the following text:

L76-80: New text added:

In Europe, the total number of reported autochthonous WNV infections in 2018 (n=2,083) exceeded, by far, the total number from the previous seven years (n=1,832). During the same transmission season, outbreaks of West Nile fever among equids increased by 30% compared to the number of outbreaks in 2017. In total, 285 outbreaks among equids were reported by the EU Member States in 2018.

New references included:

Epidemiological update: West Nile virus transmission season in Europe, 2018 [Internet]. [cited 2019 Sep 09]. Available from: https://ecdc.europa.eu/en/news-events/epidemiological-update-west-nile-virus-transmission-season-europe-2018

Also, the discussion was amended by following text:

L377-390: New text added:

The WNV transmission cycle involves mosquito vectors and birds, but equines and humans are also susceptible to infection (Kramer et al. 2007, Blitvich 2008). Although WNV infections have been described in a wide variety of vertebrates, birds are the main natural reservoir. Hundreds of wild and domestic avian species have been described as susceptible to WNV infection, but many of these showed only subclinical infection (Komar, 2003). In horses, WNV infection is also frequently clinically unapparent, but around 10% of cases develop neurological disorders with up to 50% mortality rates (Blitvich 2008, Calistri et al. 2010). An increasing number of severe outbreaks in horses have been reported in Europe in the past decade, including a large one that took place in northeast Italy in 2008 involving 251 stables with 794 cases and five deaths (Calistri et al. 2010). From the first detection of WNV in 8 out of 81 found dead wild birds in Serbia (Petrovic et al. 2013), each year WNV nucleic acid was detected in found dead or captured wild birds during summertime (Petrovic et al. 2018). Serological testing of horses sampled during 2009-2010 showed that 12% of 349 horses from the northern part of the Serbia had neutralizing WNV antibodies (Lupulovic et al. 2012). After that, each year horse WNV cases were detected by the positive serological response (IgG and IgM antibody seroconverted horses) (Petrovic et al., 2018) or as a clinical manifestation of

West Nile neuroinvasive disease (Medić et al., 2019).

New references included:

Kramer L, Li J, Shi PY. West Nile virus. Lancet Neurol. 2007;6:171–181.

Blitvich BJ. Transmission dynamics and changing epidemiology of West Nile virus. Anim Health Res Rev. 2008;9:71–86.

Komar N. West Nile virus: epidemiology and ecology in North America. Adv Vir Res. 2003;6:185–234.

Calistri P, Giovannini A, Hubalek Z, Ionescu A, Monaco F, Savini G et al. Epidemiology of West Nile in Europe and in the Mediterranean basin. Open Virol J. 2010;4(1):29–37.

Medić S, Lazić S, Petrović T, Petrić D, Samojlović M, Lazić G et al. Evidence of the first clinical case of equine neuroinvasive West Nile Disease in Serbia, 2018. Acta veterinaria 2019;69(1):123–130.

Line 28: Methods on the paper should be written in past tense: e.g., COMPARED. Review this in the rest of the manuscript, e.g., line 82.

Corrections made as suggested.

Line 30: 'the spreading of ONE malaria vector'

L33-34: *Corrections made as suggested.*

Line 37: 'Culex.' should be corrected, only Cx.?

L44: *Corrections made as suggested.*

Line 40: This is the first time you are mentioning HD, please expand the acronym, review this in the rest of the manuscript, for example EU in line 50, or ENCR in line 78.

Changed as suggested.

L40: of days with Tmax ≥ 30 °C (HD)

L47: Changed to: of days with Tmax ≥ 30 °C (Hot Days - HD)

L50: … endorsed by the EU

L58-59: Changed to: … endorsed by the European Union (EU)

L79: … using ENCR data

L93-94: Changed to: … using European Network of Cancer Registries (ENCR) data

Line 44: Specify the risk that you are addressing with this research.

Changed as suggested.

L51: New text added: … of vector-borne diseases and melanoma.

Line 54: extra 'Collected' after 'observed data', please erase.

Changed as suggested, the beginning of the sentence now reads as:

L61: In this paper, the authors collected and analysed observed data over a period

Line 55: Add 'are' after the word 'melanoma' at the end of the sentence.

L63: *Changed as suggested.*

Line 58: Here you need a reference for the environmental threat represented for the animal and humans at the Pannonian plane.

L65: *References included as suggested.*

Line 63: You need a reference for the affirmation of malaria as worldwide detrimental vector-borne disease.

L72: *New reference added as suggested.*

World Health Organization, World Malaria Report 2018. Geneva: The Organization; 2018.

Line 70: Consider adding a reference of how temperature and relative humidity are principal abiotic factors for WNV and An. hyrcanus.

Not sure how to respond to this comment. However, references concerning the vector-borne disease and mosquito vector mentioned in the sentence are already given in the text - [10,11].

Line 76: Be consistent across the whole manuscript, use either - or – without spaces to separate year timeframes, 1976–2004 is preferred.

Changed as suggested.

Line 102: Add corresponding reference for the Köpen classification.

L128: *New reference added as suggested.*

Kottek M, Grieser J, Beck C, Rudolf B, Rubel F. World Map of the Köppen-Geiger climate classification updated. Meteorologische Zeitschrift. 2006;15(3):259–263.

Line 122: "Data were..."

L148: *Changed as suggested.*

Line 126: 1985–1986.

L152-153: *Changed as suggested.*

Line 158: Briefly describe the method of WNV detection, i.e., RT-PCR or the corresponding one before referencing Petrovic et al 2018.

L193-199: New text added:

Mosquito pools were tested for WNV RNA presence by TaqMan-based one-step reverse transcription real-time PCR (RT-qPCR) that amplified both lineage 1 and 2 strains. Viral RNA was extracted using the commercial *ISOLATE II RNA Mini Kit* (Bioline, The Netherlands) according to the manufacturer's instruction. One-step RT-qPCR was conducted using the commercial kit *RNA UltraSense™ One-Step qRT-PCR System* (Life Technologies Corporation) with the primers and probe that targeted the nucleocapsid protein C gene regions of WNV, as described by Petrović et al. (2018).

Line 172: Describe the indicators briefly before referencing Jovanovic et al 2009.

According to the suggestion, the text placed between 172-174 lines is replaced by the following one.

L216-221: New text added:

In the analysis we have used two indicators: (i) melanoma incidence rate that is a measure of the number of new cases ("incidence") per unit of time ("rate") and (ii) cumulative incidence ("incidence proportion" that measures the number of new cases per person in the population over a defined period of time – often called risk or proportion). Melanoma incidence rate (per 100,000 people) for ten years 1995 - 2004 was based on the data obtained from the paper by Jovanović et al. [14]. From these data, we calculated the cumulative incidence.

Line 227: Is the formula correct: warm days - WD?

L301-302: Changed to:

air temperature Tmax ≥ 25 °C (Warm Days - WD)

Line 263: Consider changing 'indicate that the findings supporting' by 'support'

L370-374: *The sentence was quietly confusing; we rewrote it to read like this:*

Positive trends which are present in our observations might indicate that the findings on the negative influence of UVR and blue-light radiation (this radiation has a wavelength between approximately 380 nm and 500 nm; it has a very short wavelength, and so produces a higher amount of energy) on adult mosquitoes under laboratory conditions [38,39] could not be simply translated to the field.

Line 273: Authors are not showing incidence rates, just presence of WNV in mosquitoes.

L396: *Changed as suggested. End of sentence now reads as:*

… with a higher frequency of WNV presence in mosquitoes.
