# Supplementary material for: Assessment of climate change impact on the malaria vector Anopheles hyrcanus, West Nile disease, and incidence of melanoma in the Vojvodina Province (Serbia) using data from a regional climate model
Source: PLoS One. 2020 Jan 15;15(1):e0227679. doi: 10.1371/journal.pone.0227679 (PMC6961917; doi:10.1371/journal.pone.0227679)
Supplement: Attachment — Submitted filename: PONE-D-19-16900_R1_reviewer.pdf [file pone.0227679.s007.pdf]

PLOS ONE

Assessment of climate change impact on the malaria vector Anopheles hyrcanus, West Nile disease, and incidence of melanoma in the Vojvodina Province (Serbia) using data from a regional climate model

--Manuscript Draft--

Manuscript Number:	PONE-D-19-16900R1
Article Type:	Research Article
Full Title:	Assessment of climate change impact on the malaria vector Anopheles hyrcanus, West Nile disease, and incidence of melanoma in the Vojvodina Province (Serbia) using data from a regional climate model
Short Title:	Impact of climate change on malaria vector, West Nile disease, and incidence of melanoma
Corresponding Author:	Mina Petrić<br>Faculty of Sciences, University of Novi Sad; Faculty of Sciences, University of Gent; Avia-GIS NV<br>Gent, BELGIUM
Keywords:	Climate change; West Nile virus; Culex pipiens; Anopheles hyrcanus; UV radiation; Regional Climate Model; Melanoma incidence
Abstract:	Motivated by the One Health paradigm, we found the expected changes in temperature and UV radiation (UVR) to be a common trigger for enhancing the risk that viruses, vectors, and diseases pose to human and animal health. We compared data from the mosquito field collections and medical studies with regional climate model projections to examine the impact of climate change on the spreading of one malaria vector, the circulation of West Nile virus (WNV), and the incidence of melanoma. We analysed data obtained from ten selected years of standardised mosquito vector sampling with 219 unique location-year combinations, and 10 years of melanoma incidence. Trends in the observed data were compared to the climatic variables obtained by the coupled regional Eta Belgrade University and Princeton Ocean Model for the period 1961–2015 using the A1B scenario, and the expected changes up to 2030 were presented. Spreading and relative abundance of Anopheles hyrcanus was positively correlated with the trend of the mean annual temperature. We anticipated a nearly twofold increase in the number of invaded sites up to 2030. The frequency of WNV detections in Culex pipiens was significantly correlated to overwintering temperature averages and seasonal relative humidity at the sampling sites. Regression model projects a twofold increase in the incidence of WNV positive Cx. pipiens for a rise of 0.5 °C in overwintering $T_{October–April}$ temperatures. The projected increase of 56% in the number of days with $T_{max} \geq 30$ ºC (Hot Days - HD) and UVR doses (up to 1.2%) corresponds to an increasing trend in melanoma incidence. Simulations of the Pannonian countries climate anticipate warmer and drier conditions with possible dominance of temperature and number of HD over other ecological factors. These signal the importance of monitoring the changes to the preparedness of mitigating the risk of vector-borne diseases and melanoma.
Order of Authors:	Dragutin T. Mihailović
	Dušan Petrić
	Tamaš Petrović
	Ivana Hrnjaković-Cvjetković
	Vladimir Djurdjevic
	Emilija Nikolić-Đorić
	Ilija Arsenić
	Mina Petrić, MSc
	Gordan Mimić

Powered by Editorial Manager® and ProduXion Manager® from Aries Systems Corporation

	Aleksandra Ignjatović-Ćupina
**Response to Reviewers:**	Dear Dr Samy,<br><br>We are pleased to submit the revised version of "Assessment of climate change impact on the malaria vector Anopheles hyrcanus, West Nile disease, and incidence of melanoma in the Vojvodina Province (Serbia) using data from a regional climate model" (#PONE-D-19-16900). We are thankful to the academic editor for time and efforts he invested in providing detailed instructions for changes needed before resubmission.<br><br>We corrected the style in order to meet PLOS ONE's style requirements, including those for file naming.<br><br>We amended our Competing Interests Statement, and it now reads as follows:<br><br>Competing Interests Statement:<br>All authors except MP have declared that no competing interests exist. Commercial affiliation of MP is to Avia-GIS NV (commercial funder) in which she is under an employment contract since September 2016. Avia-GIS NV provided support in the form of salaries for author (MP) but did not have any additional role in the study design, data collection and analysis, decision to publish, or preparation of the manuscript. This does not alter our adherence to PLOS ONE policies on sharing data and materials.<br><br>We amended our Funding statement and Role of Funders, and it now reads as follows:<br><br>Funding Statement:<br>This paper was realised as a part of the projects "Studying climate change and its influence on the environment: impacts, adaptation and mitigation" (III43007 - DTM, DP, IHC, VDj, ENĐ, IA, GM, AIĆ) and TR31084 (DP, TP, AIĆ) financed by the Ministry of Education and Science, Republic of Serbia (http://www.mpn.gov.rs/). Historical data for mosquito vectors are the outputs of projects supported by the Veterinary Directorate, Ministry of Agriculture and Environment Protection, Republic of Serbia (http://www.minpolj.gov.rs/), and Provincial Secretariat for Science and Technological Development, the Autonomous Province of Vojvodina, project no. 114-451-2142/2011 (DP, TP, IHC, AIĆ - http://apv-visokoobrazovanje.vojvodina.gov.rs/). The funders had no role in study design, data collection and analysis, decision to publish, or preparation of the manuscript. MP is affiliated to Avia-GIS NV, which did not play a role in the study design, data collection and analysis, decision to publish, or preparation of the manuscript.<br><br>Role of Funders:<br>Avia-GIS NV provided support in the form of salaries for author (MP) but did not have any additional role in the study design, data collection and analysis, decision to publish, or preparation of the manuscript. The specific roles of the author (MP) are articulated in the 'author contributions' section.<br><br>Fig 1 copyright:<br>In compliance with the CC BY 4.0 license, we have changed Fig 1 to include a NaturalEarth baseline map (http://www.naturalearthdata.com/). We have updated the figure caption to include source information.<br><br>Authorship changes:<br>Aleksandra Ignjatović Ćupina (AIC) contributed to the acquisition of the Anopheles hyrcanus data and has been added to the list of authors. All authors are informed and express agreement regarding this change.<br><br>Corresponding authorship changes:<br>Dušan Petrić (DP) is added as the second corresponding author as he was involved in the design and sampling of Anopheles hyrcanus and Culex pipiens.<br><br>We appreciate the time and efforts by the editor and advisors in reviewing the manuscript. Please find below detailed responses to the reviewers, whom we thank for their careful consideration of the manuscript. We also reviewed the manuscript for any additional errors and made small changes that are tracked in the attached document

("Revised Manuscript with Track Changes").

Line numbers for the corrected text are given in red according to the enumeration in the file "Revised Manuscript with Track Changes".

Reviewer #1: Comments on the manuscript PONE-D-19-16900

Title: Assessment of climate change impact on malaria vectors, West Nile disease, and incidence of melanoma in the Vojvodina Province (Serbia) using data from a regional climate model. Authors: Dragutin T. Mihailović, Dušan Petrić, Tamaš Petrović, Ivana Hrnjaković Cvjetković, Vladimir Đurđević, Emilija Nikolić Đorić, Ilija Arsenić, Mina Petrić, Gordan Mimić
The work presented in paper Mihailović et al. is interesting. The objective of the authors was to compare data from the mosquito field collections and medical studies with regional 29 climate model projections to examine the impact of climate change on the circulation of West Nile virus (WNV), the spreading of the malaria vector Anopheles hyrcanus and the incidence of melanoma. The comparison was done with the coupled regional Eta Belgrade University and Princeton Ocean Model for the period 1961-2015 using the A1B scenario, and the expected changes up to 2030. Overall, significant correlation was found between the frequency of WNV in Culex pipiens and the overwintering temperature averages and seasonal relative humidity at the sampling sites. Correlation was also found between the spreading and relative abundance of Anopheles hyrcanus and the trend of the mean annual temperature. There was also an increase in melanoma incidence.
Minor comments to authors
Title
Authors wrote "malaria vectors" but the only presented data on only one vector Anopheles hyrcanus
Corrected according to the suggestion.

Abstract
L32: ……………and 10-years. Delete the hyphen
L36: Corrected according to the suggestion.
L37: ……………Culex. pipiens. Delete the dot
L44: We abbreviated the genus name to Cx. because name was spelled in the previous sentence.

Introduction

L49-53: Are the authors referring to themselves when they stated, "The authors (……….), have been working together……."?
Yes, we tried to make it clear with correction below.
L56: Corrected to: "The authors of the manuscript (……….), have been working together……."

L72: Authors should write, "Climate change…….." instead of "The climate change…….."
L86: Corrected according to the suggestion.

L75: Author should write, "Melanoma mortality…………..within the period 1985-2004
L89-91: Corrected according to the suggestion.

L78: Authors should define the acronym ENCR, as this is used here for the first time.
L83: … using ENCR data
L93-94: Changed to:
… using European Network of Cancer Registries (ENCR) data

L81-82: Authors should use past tense in the sentence "……we compared considerable of previously……"
L97: Corrected according to the suggestion.

L54-55: This sentence is not clear for me. I suggest this: "In this paper, we analysed observed data collected over a period of 31 years……"
L61: Corrected according to the suggestion, "collected" erased because of tautology.

Materials and Methods
L96: Authors should define SRES-A1B scenario for the first time.
The SRES-A1B scenario is defined in the text, and central differences to RCP explained. Due to this, authors think that selection of scenario, to some extent, is irrelevant for the presented results.
L96: … and the period 2001-2030 using the SRES-A1B scenario.
L113-122 Corrected to:
… and the period 2001-2030 according to the A1B scenario defined in Special Report on Emissions Scenarios (SRES) (Nakićenović and Swart, 2000), from now on SRES-A1B. SRES scenarios, which defined future global greenhouse gases emissions, were extensively used in the Intergovernmental Panel on Climate Change (IPCC) Third, and Fourth Assessment Reports. The main storyline behind the A1B scenario is rapid economic growth, followed by a significant increase in greenhouse gases concentrations in the future. In the Fifth Assessment Report (AR5), the Representative Concentration Pathway (RCP) is introduced, which are possible future concentration pathways without any storyline behind them. Comparing SRES-A1B and RCPs in terms of the greenhouse gases concentrations, at the end of this century SRES-A1B is the closest to RCP6.0, but for the time horizon used in this study, up to 2030, the difference between any SRES or RCPs are relatively small.
New references included:
Summary for Policy makers. In: Nakićenović N, Swart R, editors. Special Report on Emissions Scenarios. Cambridge: Cambridge University Press - Published for the Intergovernmental Panel on Climate Change. 2000. pp. 1-21

L115: Authors used only one formula, the subtitle should be in singular
L141: Corrected according to the suggestion.

Results
- Authors should specify the exact p-values instead of writing $p<0.05$ or $p>0.05$
L260-281: Corrected according to the suggestion. The section was updated to include the exact p-values.

L207: "The Poisson regression model for the dependence of a number of detections per site (frequency-λ)…………………………is highly significant". Authors stated it was highly significant, but from my perspective, $p<0.05$ is not a specific indication of high significance. Could the give the exact value of p?
L282-283: Corrected according to the suggestion. Exact p value is not below 0,01 (it is 0.01393) which is considered as high significance by many authors, so we erased the word "highly".

- Fig2b and 2c are fuzzy
Thank you for your comment. The figures were reformatted to higher resolution according to PACE.

- Fig4. Colors of fig4c are confusing
Figures 4c has been adapted to have more contrasting colors.

It will be more interesting if the authors used only vector-borne diseases data in this paper.
N.B: Other comments are incorporated in the manuscript
Authors appreciate very much the effort invested in the improvement of the manuscript quality. All suggestions are incorporated into the revised version except one concerning the spelling of NUTS. Nomenclature of territorial units for statistics is originally abbreviated NUTS from the French version (Nomenclature des Unités territoriales statistiques).

Reviewer #2:
Authors are presenting an interesting paper regarding the effects of climate change in Northern Serbia considering three independent measures: The spread of Anopheles hyrcanus, the presence of West Nile Virus in Culex pipiens, and the incidence of melanoma cases. The paper is interesting, however, discussion should be improved specially on the uncertainty of future predictions since they are using just one climatic model. Further, their results should be stated more carefully since their model rely on

Powered by Editorial Manager® and ProduXion Manager® from Aries Systems Corporation

assumptions (e.g., manually selected variables) which are also not clearly stated.
Discussion is corrected according to the reviewer's suggestion:
L345-352: New text added:
The temperature trend over the period of observations used in this study and for the future time horizon following A1B scenario obtained with the EBU-POM regional climate model is within the multi-model ensemble (MME) spread of regional climate models with similar configuration used in the ENSEMBLES project (van der Linden and Mitchell, 2009). For the period 2001-2030 the temperature change for the region of interest in the EBU-POM integration is 0.75 C concerning the period 1961-1990 and for the same period ENSEMBLES MME spread range is 0.5-1.5 C (MEP, 2017). Following this finding, other results presented in this paper that relay on temperature change, can be seen as an estimate that will be within uncertainty related to the future temperature projection.

New references included:
van der Linden P and Mitchell JFB, editors. ENSEMBLES Climate Change and its Impacts. Summary of research and results from the ENSEMBLES project. Exeter: Met Office Hadley Centre;. 2009.:
Ministry of Environmental Protection of the Republic of Serbia. Second National Communication of the Republic of Serbia under the United Nations Framework Convention on Climate Change [Internet]. Belgrade: The Ministry; 2017 Aug. 162p [cited 2019 Sep 10]. Available from: http://www.klimatskepromene.rs/wp-content/uploads/2017/09/SNC_eng.pdf

Major comments:
The paper is showing results in the order: malaria vector, WNV, and melanoma. I suggest following the same order in the abstract.
Changed as suggested.

Authors are using one of the SRES future climatic scenarios; currently the standard for future climate studies are the RCP scenarios. Authors should describe the nature of the SRES-A1B scenario, which is not mentioned in any part of the study. Further, authors should explicitly discuss uncertainty on their predictions since they are not using other scenarios or other climatic models.
Authors addressed this comment in the text corrected. Please check response to the L113-122 and L 345-352 above.

Lines 176-180. There is no discussion or results regarding these sentences. Was the comparison between EBU-POM model and the Republic Hydrometeorological Service of Serbia perfect? What is the implication of this approach on the overall paper?
This is a valuable comment since the information measure(s) is(are) a good indicator of the reliability of model outputs and thus on the overall paper. The increasing complexity of climate models is a growing concern in the modelling community. However, because we invested a serious effort to make our models more "realistic", we included more parameters and processes. With increasing model complexity, we are less able to manage and understand the model behaviour. Thus, from a user's perspective, the following question is entirely natural: "How complex model (EBU-POM model in our case) do I need to use to study this problem (assessment of climate change impact on malaria vectors, West Nile disease, and incidence of melanoma in the VPS) with this data set (temperature and/or precipitation)?". In the revised version, we inserted the additional text.
L229-249: New text added:
We considered the papers by Mihailović et al. [2,24] in which Kolmogorov complexity measures (Kolmogorov complexity (KC), Kolmogorov complexity spectrum KC spectrum) and the highest value of the KC spectrum (KCM)) and sample entropy (SE) [25] were used to quantify the regularity and complexity of air temperature and precipitation time series, obtained by the EBU-POM model, representing both deterministic chaos and stochastic processes. We considered the complexity of the EBU-POM model using the observed and modelled time series of temperature and precipitation. We computed the KC spectrum, KC, KCM and SE values for temperature and precipitation. The calculations were performed for the entire time interval 1961–1990: (1) on a daily basis with a size of N =10958 samples for temperature and (2) on a monthly basis with a size N =360 for the precipitation. The simulated time series of temperature and precipitation were obtained by the EBU-POM model for the

Powered by Editorial Manager® and ProduXion Manager® from Aries Systems Corporation

given period. The observed time series of temperature and precipitations for two stations: Sombor (SO) (88 m a.s.l.) and Novi Sad (NS) (84 m a.s.l.) in the considered area, were taken from daily meteorological reports of the Republic Hydrometeorological Service of Serbia. For both sites, the modelled complexity is lower than the observed one, but with the reliability which is in the interval values allowed by the information measures (KC, KCM, and SE) (Krzic et al. 2011, Dell' Aquila et al. 2016, Cavicchia et al. 2016). These findings mean that the models with a KC (and KCM) complexity lower than the measured time series complexity cannot always reconstruct some of the structures contained in the observed data. However, it does not mean that outputs from EBU-POM model do not correctly simulate climate elements since both sites values indicate the absence of stochastic influences, providing reliable projections of the climate elements.

New references included:

Krzic A, Tosic I, Djurdjevic V, Veljovic K, Rajkovic B. Changes in some indices over Serbia according to the SRES A1B and A2 scenarios. Climate Research. 2011;49: 73-86.

Cavicchia L, Scoccimarro E, Gualdi S, Marson P, Ahrens B, Berthou S, et al. Mediterranean extreme precipitation: a multi-model assessment. Clim. Dyn. 2016. doi: 10.1007/s00382-016-3245-x

Dell' Aquila A, Mariotti A, Bastin S, Calmanti S, Cavicchia L, Deque M, et al. Evaluation of simulated decadal variations over the Euro-Mediterranean region from ENSEMBLES to Med-CORDEX. Clim. Dyn. 2016. doi:10.1007/s00382-016-3143-2

Line 277-280: There is no evidence in this paper supporting this affirmation since the variables analyzed corresponded to three temperature related variables and just one considering humidity. Moreover, results were never compared statistically; modify accordingly.

Corrections made as suggested. The sentence "It seems that temperature in semi-urban areas dominates the other environmental factors influencing WNV circulation in nature (e.g. landscape suitability for reservoir host and mosquito vector, host availability, precipitation), as it is the primary factor affecting both mosquito vector abundance and virus replication." now reads as:

L400-404: Corrected to: It seems that temperature in semi-urban areas is an essential environmental factor influencing WNV circulation (landscape suitability for reservoir host and mosquito vector, host availability, and precipitation/water availability are somewhat similar in investigated semi-urban areas of VPS), as it affects both mosquito vector abundance and virus replication.

Figure 2: Expand the acronym CRCM. Also, double check the legend, which is describing red and green colors but the figure is only showing different shades of blue.

Corrections made as suggested.

L173-179: Fig 2. (a) The regional climate model EBU-POM projection of the mean annual air temperature (Ta) for the period 1985 - 2030 and: i) number of specimens sampled in one trap during single sampling period (blue columns); ii) the number of sites invaded by An. hyrcanus (light blue columns); and iii) relative number of positive samplings per year (dark blue columns), (b) projected increase in the number of sites invaded by An. hyrcanus (the period 2001-2030 ±S.E.), and (c) projected increase in the number of the specimens sampled in one trap during single sampling period (2001 - 2030 ±S.E.).

Figure 4: Add WD and HD to the corresponding legend of the graphic. Is there a Croatian sentence in the legend? Please describe how the melanoma incidence was calculated, is the y axis showing incidence or number of cases? Cumulative incidence is known to over-represent trends (see reference: Vandenbroucke & Pearce, 2012, doi: 10.1093/ije/dys142), try to use incidence rate instead.

This is a keystone issue in this field of epidemiology. However, it is still under a broad umbrella of discussion. In particularly mentioned reference (Vandenbroucke & Pearce, 2012) the Authors comprehensively considered the place of incidence rates in dynamic populations as well as the cumulative incidence (risk or portion) from an epistemological point of view and also giving very illustrative (educational) examples. Many authors were arguing with some ideas explicated in this paper, also considering some examples. We agree with V&P ideas, but we did not find the place where they explicitly say that it would always be using the incidence rate instead of cumulative (the

question of overestimation). To our understanding, they left the space for a situation when the use of cumulative incidence gives acceptable results. For example, it is partly seen in the paper by Wu et al. (2014). There is another moment why we used cumulative incidence. It is well-known that there is a high correlation between sun exposure (and received cumulated doses of the UV radiation) and melanoma. If that doses (or any climate element) on a daily basis are used from regional climate models, they cannot be directly correlated with daily or monthly measured or calculated biological quantities. The reason for that is the fact that from regional climate models, we can estimate just the trend of the considered physical quantity (in our case -UV doses through their cumulative values). Correspondingly it is correlated with cumulative incidence. Having said that, after the end of the statement in Line 336, we inserted the following text.
The legend in Fig. 4(c) and y-axis in Fig 4 (d) are changed as suggested.
The M&M - Melanoma incidence and UVR was amended by the following text:
L216-221: New text added:
In the analysis we have used two indicators: (i) melanoma incidence rate that is a measure of the number of new cases ("incidence") per unit of time ("rate") and (ii) cumulative incidence (“incidence proportion” that measures the number of new cases per person in the population over a defined period of time – often called risk or proportion). Melanoma incidence rate (per 100,000 people) for ten years 1995 - 2004 was based on the data obtained from the paper by Jovanović et al. [7]. From these data, we calculated the cumulative incidence.
The discussion was also amended by the following text:
L437-441: New text added:

In a cohort study, Wu et al. (2014) considered the impact of long-term UV radiation flux on skin cancer risk. Comparing with participants in the lowest quintile of cumulative UV radiation flux in adulthood, they found that participants in the highest quintile had multivariable-adjusted risks (cumulative incidence). According to Vandenbroucke and Pearce (2012), some studies where cumulative incidence is used can over-represent the trends.
New references included:
Wu S, Han J, Laden F, Qureshi AA. Long-term ultraviolet flux, other potential risk factors, and skin cancer risk: a cohort study. Cancer epidemiology, biomarkers and prevention: a publication of the American Association for Cancer Research, cosponsored by the American Society of Preventive Oncology. 2014;23(6):1080–1089.
Vandenbroucke JP, Pearce N. Incidence rates in dynamic populations. Int J Epidemiol. 2012;41(5):1472–1479.

In table S3 consider adding the number of mosquito samples per site.
Number of samples added in the table.
Figure 6 can be replaced with the statistics of such graphic for readers’ interpretation.
L442-446: Corrections made as suggested. Figure 6 deleted, the paragraph now reads as:
From a statistical point of view, the linear regression model for modelling the cumulative incidence of melanoma versus the difference of the cumulative UVR doses for hot and warm days (Fig 4d) is acceptable. Parameters are statistically highly significant ($r = 0.9712$ and $p = 0.000003$) while analysis of residual distribution shows a good agreement with the normal distribution (Shapiro-Wilk test, $W = 0.9608$, $p = 0.7952$).

Authors are justifying the paper under the ‘One Health’ concept, however they are not discussing the idea further. I would like discussing explicitly the benefits of putting together a set of multidisciplinary specialists to the development of the manuscript and how this contribution is part of the one health concept.
The discussion was amended by the following text:
L322-344: New text added:
Despite globalisation trends, researchers have become "closed" in their ever-smaller communication circles which are not limited by state or national borders but by the professional language and way of thinking. Thus, by the end of the 20th century, the scientific community has been faced with problems in communication within its confines. One of the principal reasons why vector-borne diseases (VBD) are so difficult to predict, is the complex interaction of multiple factors (vector, host, pathogen, environment including short-term weather patterns and long-term climate change) in

space and time (Moore 2008, Zimmerman 2014). Only groups from multiple sectors that communicate and work together on specific aspects of VBD systems will be able to answer the most exciting and pressing problems in the field (Moore 2008). Authors of this paper started collaboration in 2003 comparing the climates of the foci of WNV circulation in USA (California Central Valley) and Europe (Bucharest area) with VPS. As compared climates showed quite similar patterns, colleagues from public health and veterinary joined the initial group of meteorologists and medical entomologists. With the idea to better draw upon the resources and insights of the various sectors we designed and implemented research and programmes to achieve better outcomes in the control of zoonoses (diseases that can spread between animals and humans, e.g., WNV disease). This led us to the following achievements: (i) the first detection of WNV in horses in Serbia in 2009 (Lupulović 2011); (ii) the first detection of WNV in mosquitoes in Serbia in 2010 (26); (iii) the first detection of WNV in wild birds in Serbia in 2012 (Petrović 2013); (iv) development and implementation of the national programme of WNV surveillance in mosquito, bird and horse population [8], combined with human surveillance in VPS from 2014; (v) increased visibility to ECDC, EFSA and WHO; (vi) the first detection of imported dengue human case in Serbia in 2016 (Petrović 2016); and (vii) development and implementation of "One Health" programme in VPS from 2018. We are quite sure that much less would have been achieved without multidisciplinary communication and collaboration initiated in 2003, and this paper would not have been compiled.

New references included:

Moore CG. Interdisciplinary research in the ecology of vector-borne diseases: Opportunities and needs. Journal of Vector Ecology 2008;33(2):218–224.

Zimmerman B. Engaging with Complexity: Thrive! A Plan for a Healthier Nova Scotia. 2014; [e-print] Available from: https://thrive.novascotia.ca/sites/default/files/Thrive-Summit-2014-Brenda-Zimmerman-En.pdf.

Petrić D, Hrnjaković-Cvjetković I, Radovanov J, Cvjetković D, Jerant-Patić V, Milošević V, et al. West Nile virus surveillance in humans and mosquitoes and detection of cell fusing agent virus in Vojvodina Province (Serbia). HealthMED 2012;6(2):462–68.

Lupulović D, Martin-Acebes MA, Lazić S, Alonso-Padilla J, Blazquez AB, Escribano-Romero E, et al. First serological evidence of West Nile virus activity in horses in Serbia. Vector Borne Zoonotic Dis. 2011;11(9):1303–5.

Petrović T, Blazquez AB, Lupulović D, Lazić G, Escribano-Romero E, Fabijan D, et al. Monitoring West Nile virus (WNV) infection in wild birds in Serbia during 2012: First isolation and characterisation of WNV strains from Serbia. Eurosurveillance. 2013;18(44):1–8.

Petrović V, Turkulov V, Ilić S, Milošević V, Petrović M, Petrić D, et al. First report of imported case of dengue fever in Republic of Serbia. Vol. 14, Travel Medicine and Infectious Disease. 2016. p. 60–1

Minor comments:

Please use Oxford comma across the manuscript: e.g., Line 30: 'the malaria vector, and the incidence of melanoma'.

Corrections made as suggested.

Line 28: Authors never discuss problems related with animal health, thus, I suggest avoiding this kind of affirmations (e.g., line 81).

The reviewer is right, we did not, but we think it is vital to mention animals because WNV is the important zoonotic diseases. Therefore, we would like to include new paragraphs in Introduction and Discussion.

The introduction was amended by the following text:

L76-80: New text added:

In Europe, the total number of reported autochthonous WNV infections in 2018 (n=2,083) exceeded, by far, the total number from the previous seven years (n=1,832). During the same transmission season, outbreaks of West Nile fever among equids increased by 30% compared to the number of outbreaks in 2017. In total, 285 outbreaks among equids were reported by the EU Member States in 2018.

New references included:

Epidemiological update: West Nile virus transmission season in Europe, 2018 [Internet]. [cited 2019 Sep 09]. Available from: https://ecdc.europa.eu/en/news-events/epidemiological-update-west-nile-virus-transmission-season-europe-2018

Also, the discussion was amended by following text:

L377-390: New text added:

The WNV transmission cycle involves mosquito vectors and birds, but equines and

humans are also susceptible to infection (Kramer et al. 2007, Blitvich 2008). Although WNV infections have been described in a wide variety of vertebrates, birds are the main natural reservoir. Hundreds of wild and domestic avian species have been described as susceptible to WNV infection, but many of these showed only subclinical infection (Komar, 2003). In horses, WNV infection is also frequently clinically unapparent, but around 10% of cases develop neurological disorders with up to 50% mortality rates (Blitvich 2008, Calistri et al. 2010). An increasing number of severe outbreaks in horses have been reported in Europe in the past decade, including a large one that took place in northeast Italy in 2008 involving 251 stables with 794 cases and five deaths (Calistri et al. 2010). From the first detection of WNV in 8 out of 81 found dead wild birds in Serbia (Petrovic et al. 2013), each year WNV nucleic acid was detected in found dead or captured wild birds during summertime (Petrovic et al. 2018). Serological testing of horses sampled during 2009-2010 showed that 12% of 349 horses from the northern part of the Serbia had neutralizing WNV antibodies (Lupulovic et al. 2012). After that, each year horse WNV cases were detected by the positive serological response (IgG and IgM antibody seroconverted horses) (Petrovic et al., 2018) or as a clinical manifestation of West Nile neuroinvasive disease (Medić et al., 2019).

New references included:

Kramer L, Li J, Shi PY. West Nile virus. Lancet Neurol. 2007;6:171–181.
Blitvich BJ. Transmission dynamics and changing epidemiology of West Nile virus. Anim Health Res Rev. 2008;9:71–86.
Komar N. West Nile virus: epidemiology and ecology in North America. Adv Vir Res. 2003;6:185–234.
Calistri P, Giovannini A, Hubalek Z, Ionescu A, Monaco F, Savini G et al. Epidemiology of West Nile in Europe and in the Mediterranean basin. Open Virol J. 2010;4(1):29–37.
Medić S, Lazić S, Petrović T, Petrić D, Samojlović M, Lazić G et al. Evidence of the first clinical case of equine neuroinvasive West Nile Disease in Serbia, 2018. Acta veterinaria 2019;69(1):123–130.

Line 28: Methods on the paper should be written in past tense: e.g., COMPARED. Review this in the rest of the manuscript, e.g., line 82.
Corrections made as suggested.

Line 30: ‘the spreading of ONE malaria vector’
L33-34: Corrections made as suggested.

Line 37: ‘Culex.’ should be corrected, only Cx.?
L44: Corrections made as suggested.

Line 40: This is the first time you are mentioning HD, please expand the acronym, review this in the rest of the manuscript, for example EU in line 50, or ENCR in line 78.
Changed as suggested.
L40: of days with Tmax ≥ 30 C (HD)
L47: Changed to: of days with Tmax ≥ 30 C (Hot Days - HD)
L50: … endorsed by the EU
L58-59: Changed to: … endorsed by the European Union (EU)
L79: … using ENCR data
L93-94: Changed to: … using European Network of Cancer Registries (ENCR) data

Line 44: Specify the risk that you are addressing with this research.
Changed as suggested.
L51: New text added: … of vector-borne diseases and melanoma.

Line 54: extra ‘Collected’ after ‘observed data’, please erase.
Changed as suggested, the beginning of the sentence now reads as:
L61: In this paper, the authors collected and analysed observed data over a period

Line 55: Add ‘are’ after the word ‘melanoma’ at the end of the sentence.
L63: Changed as suggested.

Line 58: Here you need a reference for the environmental threat represented for the animal and humans at the Pannonian plane.
L65: References included as suggested.

Powered by Editorial Manager® and ProduXion Manager® from Aries Systems Corporation

Line 63: You need a reference for the affirmation of malaria as worldwide detrimental vector-borne disease.
L72: New reference added as suggested.
World Health Organization, World Malaria Report 2018. Geneva: The Organization; 2018.

Line 70: Consider adding a reference of how temperature and relative humidity are principal abiotic factors for WNV and An. hyrcanus.
Not sure how to respond to this comment. However, references concerning the vector-borne disease and mosquito vector mentioned in the sentence are already given in the text - [10,11].

Line 76: Be consistent across the whole manuscript, use either - or – without spaces to separate year timeframes, 1976–2004 is preferred.
Changed as suggested.

Line 102: Add corresponding reference for the Köpen classification.
L128: New reference added as suggested.
Kottek M, Grieser J, Beck C, Rudolf B, Rubel F. World Map of the Köppen-Geiger climate classification updated. Meteorologische Zeitschrift. 2006;15(3):259–263.

Line 122: “Data were...”
L148: Changed as suggested.

Line 126: 1985–1986.
L152-153: Changed as suggested.

Line 158: Briefly describe the method of WNV detection, i.e., RT-PCR or the corresponding one before referencing Petrovic et al 2018.
L193-199: New text added:
Mosquito pools were tested for WNV RNA presence by TaqMan-based one-step reverse transcription real-time PCR (RT-qPCR) that amplified both lineage 1 and 2 strains. Viral RNA was extracted using the commercial ISOLATE II RNA Mini Kit (Bioline, The Netherlands) according to the manufacturer's instruction. One-step RT-qPCR was conducted using the commercial kit RNA UltraSense™ One-Step qRT-PCR System (Life Technologies Corporation) with the primers and probe that targeted the nucleocapsid protein C gene regions of WNV, as described by Petrović et al. (2018).

Line 172: Describe the indicators briefly before referencing Jovanovic et al 2009.
According to the suggestion, the text placed between 172-174 lines is replaced by the following one.
L216-221: New text added:
In the analysis we have used two indicators: (i) melanoma incidence rate that is a measure of the number of new cases ("incidence") per unit of time ("rate") and (ii) cumulative incidence (“incidence proportion” that measures the number of new cases per person in the population over a defined period of time – often called risk or proportion). Melanoma incidence rate (per 100,000 people) for ten years 1995 - 2004 was based on the data obtained from the paper by Jovanović et al. [14]. From these data, we calculated the cumulative incidence.

Line 227: Is the formula correct: warm days - WD?
L301-302: Changed to:
air temperature Tmax ≥ 25 C (Warm Days - WD)

Line 263: Consider changing ‘indicate that the findings supporting’ by ‘support’
L370-374: The sentence was quietly confusing; we rewrote it to read like this:
Positive trends which are present in our observations might indicate that the findings on the negative influence of UVR and blue-light radiation (this radiation has a wavelength between approximately 380 nm and 500 nm; it has a very short wavelength, and so produces a higher amount of energy) on adult mosquitoes under laboratory conditions [38,39] could not be simply translated to the field.

Line 273: Authors are not showing incidence rates, just presence of WNV in

Powered by Editorial Manager® and ProduXion Manager® from Aries Systems Corporation

	mosquitoes.<br>L396: Changed as suggested. End of sentence now reads as:<br>… with a higher frequency of WNV presence in mosquitoes.
Additional Information:	
Question	Response
**Financial Disclosure**<br><br>Enter a financial disclosure statement that describes the sources of funding for the work included in this submission. Review the submission guidelines for detailed requirements. View published research articles from *PLOS ONE* for specific examples.<br><br>This statement is required for submission and **will appear in the published article** if the submission is accepted. Please make sure it is accurate.<br><br>**Unfunded studies**<br>Enter: *The author(s) received no specific funding for this work.*<br><br>**Funded studies**<br>Enter a statement with the following details:<br>• Initials of the authors who received each award<br>• Grant numbers awarded to each author<br>• The full name of each funder<br>• URL of each funder website<br>• Did the sponsors or funders play any role in the study design, data collection and analysis, decision to publish, or preparation of the manuscript?<br>• **NO** - Include this sentence at the end of your statement: *The funders had no role in study design, data collection and analysis, decision to publish, or preparation of the manuscript.*<br>• **YES** - Specify the role(s) played.<br><br>* typeset	This paper was realised as a part of the projects "Studying climate change and its influence on<br>the environment: impacts, adaptation and mitigation" (III43007 - DTM, DP, IHC, VDj, ENĐ, IA,<br>GM, AIĆ) and TR31084 (DP, TP, AIĆ) financed by the Ministry of Education and Science,<br>Republic of Serbia (http://www.mpn.gov.rs/). Historical data for mosquito vectors are the<br>outputs of projects supported by the Veterinary Directorate, Ministry of Agriculture and Environment Protection, Republic of Serbia (http://www.minpolj.gov.rs/), and Provincial Secretariat for Science and Technological Development, the Autonomous Province of Vojvodina, project no. 114-451-2142/2011 (DP, TP, IHC, AIĆ - http://apv-visokoobrazovanje.vojvodina.gov.rs/). The funders had no role in study design, data collection<br>and analysis, decision to publish, or preparation of the manuscript. MP is affiliated to Avia-GIS<br>NV, which did not play a role in the study design, data collection and analysis, decision to<br>publish, or preparation of the manuscript.<br><br>Role of Funders<br><br>Avia-GIS NV provided support in the form of salaries for author (MP) but did not have any<br>additional role in the study design, data collection and analysis, decision to publish, or preparation of the manuscript. The specific roles of the author (MP) are articulated in the ‘author<br>contributions’ section.
**Competing Interests**<br><br>Use the instructions below to enter a competing interest statement for this submission. On behalf of all authors, disclose any competing interests that could be perceived to bias this work—acknowledging all financial support	The authors have declared that no competing interests exist.

and any other relevant financial or non-financial competing interests.<br><br>This statement **will appear in the published article** if the submission is accepted. Please make sure it is accurate. View published research articles from *PLOS ONE* for specific examples.<br><br>**NO authors have competing interests**<br><br>Enter: *The authors have declared that no competing interests exist.*<br><br>**Authors with competing interests**<br><br>Enter competing interest details beginning with this statement:<br><br>*I have read the journal's policy and the authors of this manuscript have the following competing interests: [insert competing interests here]*<br><br>* typeset	
**Ethics Statement**<br><br>Enter an ethics statement for this submission. This statement is required if the study involved:<br><br>• Human participants<br>• Human specimens or tissue<br>• Vertebrate animals or cephalopods<br>• Vertebrate embryos or tissues<br>• Field research<br><br>Write "N/A" if the submission does not require an ethics statement.<br><br>General guidance is provided below. Consult the submission guidelines for detailed instructions. **Make sure that all information entered here is included in the Methods section of the manuscript.**	N/A

**Format for specific study types**<br><br>**Human Subject Research (involving human participants and/or tissue)**<br>• Give the name of the institutional review board or ethics committee that approved the study<br>• Include the approval number and/or a statement indicating approval of this research<br>• Indicate the form of consent obtained (written/oral) or the reason that consent was not obtained (e.g. the data were analyzed anonymously)<br><br>**Animal Research (involving vertebrate animals, embryos or tissues)**<br>• Provide the name of the Institutional Animal Care and Use Committee (IACUC) or other relevant ethics board that reviewed the study protocol, and indicate whether they approved this research or granted a formal waiver of ethical approval<br>• Include an approval number if one was obtained<br>• If the study involved *non-human primates*, add *additional details* about animal welfare and steps taken to ameliorate suffering<br>• If anesthesia, euthanasia, or any kind of animal sacrifice is part of the study, include briefly which substances and/or methods were applied<br><br>**Field Research**<br><br>Include the following details if this study involves the collection of plant, animal, or other materials from a natural setting:<br>• Field permit number<br>• Name of the institution or relevant body that granted permission	
**Data Availability**<br><br>Authors are required to make all data underlying the findings described fully available, without restriction, and from the time of publication. PLOS allows rare exceptions to address legal and ethical concerns. See the PLOS Data Policy and FAQ for detailed information.	Yes - all data are fully available without restriction

A Data Availability Statement describing where the data can be found is required at submission. Your answers to this question constitute the Data Availability Statement and **will be published in the article**, if accepted.<br><br>**Important:** Stating 'data available on request from the author' is not sufficient. If your data are only available upon request, select 'No' for the first question and explain your exceptional situation in the text box.<br><br>Do the authors confirm that all data underlying the findings described in their manuscript are fully available without restriction?	
**Describe where the data may be found in full sentences. If you are copying our sample text, replace any instances of XXX with the appropriate details.**<br><br>• If the data are **held or will be held in a public repository**, include URLs, accession numbers or DOIs. If this information will only be available after acceptance, indicate this by ticking the box below. For example: *All XXX files are available from the XXX database (accession number(s) XXX, XXX.*).<br>• If the data are all contained **within the manuscript and/or Supporting Information files**, enter the following: *All relevant data are within the manuscript and its Supporting Information files.*<br>• If neither of these applies but you are able to provide **details of access elsewhere**, with or without limitations, please do so. For example:<br><br>*Data cannot be shared publicly because of [XXX]. Data are available from the XXX Institutional Data Access / Ethics Committee (contact via XXX) for researchers who meet the criteria for access to confidential data.*<br><br>*The data underlying the results presented in the study are available from (include the name of the third party*	All relevant data are within the manuscript and its Supporting Information files.

and contact information or URL).<br>• This text is appropriate if the data are owned by a third party and authors do not have permission to share the data.<br><br>* typeset	
Additional data availability information:	

Manuscript

Click here to access/download;Manuscript;Mihailovic2019_Manuscript.docx

Assessment of climate change impact on the malaria vector *Anopheles hyrcanus*, West Nile disease, and incidence of melanoma in the Vojvodina Province (Serbia) using data from a regional climate model

Dragutin T. Mihailović[1], Dušan Petrić[2*], Tamaš Petrović[3], Ivana Hrnjaković-Cvjetković[4,5], Vladimir Djurdjevic[6], Emilija Nikolić-Đorić[7], Ilija Arsenić[1], Mina Petrić[8,9,10*], Gordan Mimić[11], Aleksandra Ignjatović-Ćupina[2]

[1] Department of Field and Vegetable Crops, Faculty of Agriculture, University of Novi Sad, Novi Sad, Serbia

[2] Department of Plant and Environment Protection, Faculty of Agriculture, University of Novi Sad, Novi Sad, Serbia

[3] Department for virology, Scientific Veterinary Institute "Novi Sad", Novi Sad, Serbia

[4] Institute of Public Health of Vojvodina, Novi Sad, Serbia

[5] Faculty of Medicine, University of Novi Sad, Novi Sad, Serbia

[6] Institute of Meteorology, Faculty of Physics, University of Belgrade, Belgrade, Serbia

[7] Department of Agricultural Economics, Faculty of Agriculture, University of Novi Sad, Novi Sad, Serbia

[8] Avia-GIS NV, Zoersel, Belgium

[9] Department of Physics, Faculty of Sciences, University of Novi Sad, Novi Sad, Serbia

[10] Department of Physics and Astronomy, Faculty of Sciences, University of Gent, Gent, Belgium

[11] BioSense Institute, University of Novi Sad, Novi Sad, Serbia

* Corresponding authors E-mail:

mpetric@avia-gis.com (MP)

dusanp@polj.uns.ac.rs (DP)

Abstract

Motivated by the One Health paradigm, we found the expected changes in temperature and UV radiation (UVR) to be a common trigger for enhancing the risk that viruses, vectors, and diseases pose to human and animal health. We compared data from the mosquito field collections and medical studies with regional climate model projections to examine the impact of climate change on the spreading of one malaria vector, the circulation of West Nile virus (WNV), and the incidence of melanoma. We analysed data obtained from ten selected years of standardised mosquito vector sampling with 219 unique location-year combinations, and 10 years of melanoma incidence. Trends in the observed data were compared to the climatic variables obtained by the coupled regional Eta Belgrade University and Princeton Ocean Model for the period 1961–2015 using the A1B scenario, and the expected changes up to 2030 were presented. Spreading and relative abundance of *Anopheles hyrcanus* was positively correlated with the trend of the mean annual temperature. We anticipated a nearly twofold increase in the number of invaded sites up to 2030. The frequency of WNV detections in *Culex pipiens* was significantly correlated to overwintering temperature averages and seasonal relative humidity at the sampling sites. Regression model projects a twofold increase in the incidence of WNV positive *Cx. pipiens* for a rise of 0.5 °C in overwintering $T_{October–April}$ temperatures. The projected increase of 56% in the number of days with $T_{max} \geq$ 30 ° C (Hot Days - HD) and UVR doses (up to 1.2%) corresponds to an increasing trend in melanoma incidence. Simulations of the Pannonian countries climate anticipate warmer and drier conditions with possible dominance of temperature and number of HD over other ecological factors. These signal the importance of monitoring the changes to the preparedness of mitigating the risk of vector-borne diseases and melanoma.

Introduction

Climate change is referred to as “the biggest global health threat of the 21st century” [1]. The analysis of outputs from all general circulation models (GCM) suggests that the countries of the Pannonian Plain,

including Serbia, are facing significant impacts of climate change, affecting all aspects of human life [2]. The authors of the manuscript (meteorology, entomology, veterinary medicine, and public health experts), have been working together since 2003, promoting the idea of multisectoral collaboration before the One Health Concept was officially inaugurated in the USA in 2007 [3], and endorsed by the European Union (EU) [4] as well as prominent organizations such as the World Health Organization (WHO), Food and Agriculture Organization (FAO), and the World Organization for Animal Health (OIE) in 2018 [5].

In this paper, the authors collected and analysed observed data over 31 years and related a subset to outputs from a Regional Climate Model (RCM). Vector-borne diseases and melanoma are significant climate-driven threats for which risk sources can be clearly defined [6]. Moreover, both present progressively growing environmental threats to the animal as well as human health in the countries of the Pannonian Plane [7,8,9].

The biology and distribution of mosquito vectors and their capacity to transmit mosquito-borne diseases are dependent on many factors such as global trade and travel, urbanisation, habitat destruction, pesticide application, host density, and climate. *Anopheles hyrcanus* and *Culex pipiens* are mosquito species that are vectors of malaria and West Nile virus (WNV) disease, respectively, the two most detrimental vector-borne diseases worldwide [10,11]. Malaria was eradicated from Serbia and other Balkan states during the last century. However, the spreading of its vectors (*Anopheles* mosquitoes) and the re-emergence of the disease in Greece [12] pose a threat to the South East and Central Europe once again. In 2018, Serbia was the second European country (after Italy) most affected by WNV disease (415 reported cases with 35 fatal outcomes). In Europe, the total number of reported human autochthonous WNV infections in 2018 (n=2,083) exceeded, by far, the total number from the previous seven years (n=1,832). During the same transmission season, outbreaks of West Nile fever among equids increased by 30% compared to the number of outbreaks in 2017. In total, 285 outbreaks among equids were reported by the EU Member States in 2018 [13]. Current evidence suggests that inter-annual and inter-decadal climate variability have a direct influence on the epidemiology of vector-borne diseases, with temperature and relative humidity as the principal abiotic factors influencing the life-cycles of the mosquito vector, the pathogen, the host

and the interactions between them [14,15].

Melanoma is a malignant disease that has experienced a significant increase in incidence during the last few decades all over the world [16]. Climate change impact on melanoma should be considered as a synergy of changes in UV radiation (UVR) due to stratospheric ozone depletion and the long-term increase of air temperature leading to more prolonged exposure of individuals to UVR doses and consequently to a higher risk of melanoma [17]. Melanoma mortality in the Vojvodina Province (northern Serbia) (VPS) within the period 1985–2004 shows an evident increase, placing it amongst the most vulnerable regions in the world. Thus, Jovanović et al. [7] estimated and made the list of mortality rates from malignant melanoma for males (age-standardised rate/100,000) in Europe (39 countries) for the year 2000, using European Network of Cancer Registries (ENCR) data. This list shows that the VPS is among the top eleven states (six of them have parts in the Pannonian Plane) listed as the most endangered.

In this study, devoted to revealing the potential impact of climate change on animal and human health, we compared a considerable amount of previously unpublished ecological data obtained from the field and clinical surveys with climate change projections for the VPS, which is representative of the Central European low-altitude areas with a human-dominated landscape (Fig 1). We examined the effects of temperature on the spread and relative abundance of the malaria vector *An. hyrcanus* and the "microclimate" differentiation between sites with a specific frequency of WNV occurrence in *Cx. pipiens* . We also evaluated the impact of climate change on melanoma incidence as a synergy of changes in UVR doses and the long-term increase in the number of hot days (HD), with daily maximum temperature ≥ 30ºC using the Eta Belgrade University and Princeton Ocean Model (EBU-POM) regional model data.

Fig 1. (a) Location of the Vojvodina Province (Serbia) in Europe and (b) altitude map. (Made with Natural Earth - naturalearthdata.com)

Materials and Methods

For the assessment of the climate change and the impact of UVR doses, we used the climatic variables obtained by the coupled regional EBU-POM model for the historical period 1961–2000 and the period 2001-2030 according to the A1B scenario defined in Special Report on Emissions Scenarios (SRES) [18], from now on SRES-A1B. SRES scenarios, which defined future global greenhouse gases emissions, were extensively used in the Intergovernmental Panel on Climate Change (IPCC) Third, and Fourth Assessment Reports. The main storyline behind the A1B scenario is rapid economic growth, followed by a significant increase in greenhouse gases concentrations in the future. In the Fifth Assessment Report (AR5), the Representative Concentration Pathway (RCP) is introduced, which are possible future concentration pathways without any storyline behind them. Comparing SRES-A1B and RCPs in terms of the greenhouse gases concentrations, at the end of this century SRES-A1B is the closest to RCP6.0, but for the time horizon used in this study, up to 2030, the difference between any SRES or RCPs are relatively small.

Study area and climate

The VPS is situated in the northern part of Serbia and the southern part of the Pannonian lowland (18°51′–21°33′E, 44°37′–46°11′N and 75–641 m.a.s.l. (with the Fruška Gora Mountain in the south) as it is seen in Figs 1a and 1b). This region is the essential food production area in Serbia with a total surface area of 21,500 km^2 and a population of about 2 million. This region has a continental climate, with elements of a sub-humid and warm climate (Cfwbx" according to Köppen classification [19]).

Models and formula used

The global and regional climate model

For climate simulations in this study, we used results of the EBU-POM model runs for the SRES-A1B scenario integrated over the period 2001–2030 [20]. The EBU-POM is a two-way, coupled RCM. The atmospheric part is the Eta/National Centres for Environmental Prediction (NCEP) limited area model

(resolution 0.25° × 0.25° on 32 vertical levels; centred at 41.5° N, 15° E, with boundaries at ±19.9° W–E and ±13.0° S–N), while the oceanic part is the POM (resolution 0.20° × 0.20° on 21 vertical levels). The driving global circulation model (GCM) was the ECHAM5 model [21] coupled with the Max Planck Institute Ocean Model (MPI-OM) [22]. More details about model integrations and performed bias correction for VPS can be found in the paper by Mihailović et al. [2]. The POM model was set over the Mediterranean Sea without the Black Sea; for other open seas, the sea surface temperature from the GCM was used as a bottom boundary condition.

Empirical formula

For calculating the daily doses of UVR, i.e. UVRD in the study area sites we have used the following empirical formula $UVRD = 0.002507 \times G_d - 5.985$ (kJ/m2) derived by Malinović-Milićević et al. [23], where G_d is the daily sum of global solar radiation.

Environmental sampling

Mosquito vectors

We used standardised protocols to measure mosquito presence/absence, density, and infestation by WNV. Data were extracted from dry ice-baited trap samples, collected over 31 years at 166 different sites (745 sampled locations, S1 Table) in the VPS, to infer on the trends of local vector status and virus circulation in mosquitoes. In all years mosquitoes were sampled from May to September, with different spatial intensity and time-frequency governed by the scale and scope of different research projects. For comparison with climate variables, we extracted data obtained in 10 years (1985–6, 2004–2005 and 2010–2015) for which a standardised surveillance protocol was in place. These periods have the highest number of particular location-year combinations (S1-S3 Tables).

Samples were collected by two different types of dry-ice baited suction traps. During 1985 and 1986 [24,25] by the miniature CDC light trap (CDC) and for 2004 and 2015 by the NS2 trap (our design of dry ice-baited suction trap without light). Both traps were operating without a light source (incandescent light

proved not to be attractive/repellent for most mosquito species inhabiting the VPS [25]). The CDC trap has 3–5 times stronger suction power (operated by a 9 V battery) than NS2 (operated by 3 x 1.2 V batteries), meaning that the increase in density of species observed after 1986 could not be attributed to the change of the type of trap. Traps were operated from the afternoon until the morning of the next day (one trap night), with different periodicity. The specific location of the trap at each site was chosen by experienced entomologists to stabilise variation of the collected data.

We used three parameters to indicate *An. hyrcanus* spread and population growth in the period 1985-2015: i) the ratio of positive to total mosquito samplings per year; ii) the number of sites invaded (positive places where it was looked for, but was not found in the preceding sampling period, and the number of sites where was observed in both periods, i.e. established); and iii) the average number of specimens sampled in one trap during single sampling period from the afternoon of the starting day to the morning of the next day (Figs 2a and 2c). Here, we used data from 1,073 mosquito samples (1985–6, 2004–5 and 2014–5), obtained at 54 location over six years (142 unique location-year combinations) (S2 Table).

Fig 2. (a) The regional climate model EBU-POM projection of the mean annual air temperature (T_a) for the period 1985–2030 and: i) number of specimens sampled in one trap during single sampling period (blue columns); ii) the number of sites invaded by *An. hyrcanus* (light blue columns); and iii) relative number of positive samplings per year (dark blue columns), (b) projected increase in the number of sites invaded by *An. hyrcanus* (the period 2001–2030 ±S.E.), and (c) projected increase in the number of the specimens sampled in one trap during single sampling period (2001–2030 ±S.E.).

For *Cx. pipiens*, the period starting with the first detection of WNV in mosquitoes in Serbia, in 2010 [26], to 2015 was considered. To investigate the impact of microclimate on the complex interaction between *Cx. pipiens* and WNV, we used the following climatic parameters from the EBU-POM model outputs (covering the period 2006–2015) for 11 sites (GPS coordinates – S3 Table) in the VPS with different

histories of WNV circulation: (i) mean annual temperature (T_a); (ii) overwintering temperature (T_{oa}) for the period October – April; and (iii) seasonal temperature (T_{ms}) and relative humidity (R_{ms}) for the period May – September. For these sites, we examined the correlation between the frequency of WNV detections in *Cx. pipiens* at each site (from 2010to 2015) and the corresponding period averages of climate time series for the same site. For detection of WNV, specimens were sampled, anaesthetised by dry ice, identified to species level [27] on dry ice-cooled paper, pooled according to date, location, sex, and species, transported on dry ice to the laboratory, and stored at -70 °C before virus detection. Pool size did not exceed 50 mosquito specimens per pool. Mosquito pools were tested for WNV RNA presence by TaqMan-based one-step reverse transcription real-time PCR (RT-qPCR) that amplified both lineage 1 and 2 strains. Viral RNA was extracted using the commercial *ISOLATE II RNA Mini Kit* (Bioline, The Netherlands) according to the manufacturer's instruction. One-step RT-qPCR was conducted using the commercial kit *RNA UltraSense™ One-Step qRT-PCR System* (Life Technologies Corporation) with the primers and probe that targeted the nucleocapsid protein C gene regions of WNV, as described by Petrović et al. [8]. We analysed the yearly occurrence of the WNV positive *Cx. pipiens* mosquitoes sampled by dry ice-baited traps in the years 2010–2015 across 66 unique location-year combinations (S3 Table). Only traps positioned precisely at the same spot over the entire six-year period are considered for analysis. Numbers allocated to different places (Fig 3) indicate the number of years in the period 2010–2015 in which WNV was detected in sampled *Cx. pipiens* mosquitoes; e.g. 5 indicates that WNV positive *Cx. pipiens* were detected in five out of the six years in the samples collected from the same spot.

Fig 3. (a) Dependence of frequencies (λ) of WNV positive *Culex pipiens* detections at the same site on overwintering temperatures (Toa); (b) Frequency of sampling of WNV infected mosquitoes (1 – 5 times) during six years (bars and numbers) in NUTS3 (Nomenclature of Territorial Units for Statistics) units of the Vojvodina Province, Serbia.

Melanoma incidence and UVR

In the analysis we have used two indicators: (i) melanoma incidence rate that is a measure of the number of new cases ("incidence") per unit of time ("rate") and (ii) cumulative incidence (“incidence proportion” that measures the number of new cases per person in the population over a defined period of time – often called risk or proportion). Melanoma incidence rate (per 100,000 people) for ten years 1995 - 2004 was based on the data obtained from the paper by Jovanović et al. [7]. From these data, we calculated the cumulative incidence. We have used the model simulation to study the expected impact of climate change on UVR exposure of human skin for nine sites in VPS [PA (Palić), SO (Sombor), KI (Kikinda), NS (Novi Sad), BC (Bečej, ZR (Zrenjanin), SM (Sremska Mitrovica), BK (Bantaski Karlovac), and BG (Beograd)]. Firstly, we calculated daily UVR doses (UVRD) from global radiation model outputs using the empirical formula for the nine sites for the period April-September, and then we found the relative change R(UVRD) of those doses as $R(UVRD) = (UVRD-UVRD_k) / UVRD_k$ where $UVRD_k$ is the dose for 1961–1990 reference period, while the UVRD is calculated for the period 2001–2030.

Statistics

We considered the papers by Mihailović et al. [2,28] in which Kolmogorov complexity measures (Kolmogorov complexity (KC), Kolmogorov complexity spectrum KC spectrum) and the highest value of the KC spectrum (KCM)) and sample entropy (SE) [29] were used to quantify the regularity and complexity of air temperature and precipitation time series, obtained by the EBU-POM model, representing both deterministic chaos and stochastic processes. We considered the complexity of the EBU-POM model using the observed and modelled time series of temperature and precipitation. We computed the KC spectrum, KC, KCM and SE values for temperature and precipitation. The calculations were performed for the entire time interval 1961–1990: (i) on a daily basis with a size of N =10958 samples for temperature and (ii) on a monthly basis with a size N =360 for the precipitation. The simulated time series of temperature and precipitation were obtained by the EBU-POM model for the given period. The observed time series of temperature and precipitations for two stations: Sombor (SO)

(88 m.a.s.l.) and Novi Sad (NS) (84 m.a.s.l.) in the considered area, were taken from daily meteorological reports of the Republic Hydrometeorological Service of Serbia. For both sites, the modelled complexity is lower than the observed one, but with the reliability which is in the interval values allowed by the information measures (KC, KCM, and SE) [30,31,32]. These findings mean that the models with a KC (and KCM) complexity lower than the measured time series complexity cannot always reconstruct some of the structures contained in the observed data. However, it does not mean that outputs from EBU-POM model do not correctly simulate climate elements since both sites values indicate the absence of stochastic influences, providing reliable projections of the climate elements.

For *An. hyrcanus*, the temperature trend was evaluated by the Mann-Kendall test using the R statistical package [33]. Field observed values on species distribution and density for the period 1985–2015 and forecasts of the numbers of sites invaded and specimens sampled for the period 2016-2030 based on linear trend were obtained by the Eviews 9.5 software [34]. For *Cx. pipiens*, the relationship between yearly frequency of WNV detection in mosquitoes, air temperature, and relative humidity (derived from the climate model) was estimated using Spearman's Rank-Order Correlation and a Poisson regression model (Statistica 13 [35]). For melanoma, the linear regression model was used for modelling the cumulative incidence of melanoma versus the difference of the cumulative UVR doses for hot and warm days (Fig 4d). Analysis of residual distribution was done by Shapiro-Wilk test (Statistica 13 [35])

Results

Mosquito vectors. Fig 2a shows an evident linear trend of the mean annual temperature T_a for the period 1985–2030 (r = 0.467; p = 0.001; τ = 0.328) calculated from the EBU-POM regional model outputs for 29 representative sites in the VPS. All parameters that were chosen for the evaluation of the spread and population increase of *An. hyrcanus* were positively, but to a different extent, correlated to the time argument (periods in which sampling was performed since the beginning of monitoring in 1985) indicating a monotonic trend. The increase of parameters follows the trend of T_a (Fig 2a). The strongest

correlation was found for the increase in the ratio of positive samplings (r = 0.986; p = 0.000307; τ = 0.828), followed by the number of mosquitoes per trap night (r = 0.919; p = 0.009639; τ = 0.733), and the number of sites invaded (r = 0.889; p = 0.01766; τ = 0.6). By 2030 we anticipate a further increase in numbers of invaded sites and adult females sampled, by 1.71 and 1.27 fold, respectively (Figs 2b and 2c).

Spearman rank-order correlation between the frequency of WNV detections in *Cx. pipiens* at 11 sites and the corresponding mean values of climate time series -was the highest for T_{oa} (r = 0.755; p = 0.00008), then for T_a (r = 0.616; p = 0.00294), R_{ms} (r = 0.499; p = 0.02119), and T_{ms} (r = 0.477; p = 0.02856). Fig 3a depicts the Poisson regression model for the dependence of a number of detections per site (frequency - λ) on T_{oa}, which is significant (p = 0.01393). The output of the model ($ln\lambda = -7.923 + 1.533 \times T_{OA}$) indicates that for an increase of 0.5 °C in T_{oa} (presuming that all other factors needed for the circulation of WNV are kept constant), a twofold increase in the incidence of WNV positive *Cx. pipiens* could be projected. Fig 3b depicts that most of the sites with the high frequency of WNV occurrence (≥ 2) were distributed along the northwest-southeast axis of the VPS.

Melanoma incidence and UVR doses. Fig 4b shows the positive relative change of UVRD, remarkably covering an eastern, southern, western, and partly central area of VPS. Specifically, the projected increase is twofold going from the west and northwest (0.60%) towards the east and southeast where it reaches values of about 1.20%. The EBU-POM model (for nine sites) shows a significant expected increase of 56% in the number of HD days in the VPS (Fig 4a), compared to the period 1961–1990. Additionally, we observed a decrease of 1.1% in the number of days with maximum air temperature Tmax ≥ 25 °C (Warm Days - WD). This prolongs the exposure of outdoor working adults to UVR and thus leads to an increase in melanoma risk. This risk becomes even more significant because of the increase in cumulative values of UVR doses (Fig 4c). Fig 4d depicts the cumulative incidence of melanoma for the period 1985–2004 with an increasing monotonic trend (r = 0.9712 and p = 0.000003).

Fig 4. (a) Relative change of hot days (HD) and (b) UVR radiation doses [R (UVRD)] for the period

2001–2030 compared to the period 1961–1990; (c) cumulative values of mean UVR doses for the period 1985–2030 (averaged for seven sites: PA, SO, BC, KI, NS, ZR, SM, and BK) under the SRES-A1B scenario (for WD and HD days); and (d) cumulative incidence of melanoma for the period 1995–2004 in the Vojvodina Province, Serbia.

Discussion

Here we presented an intriguing comparison of the impact of climate change on complex systems including mosquito vectors, pathogens, and melanoma, which are all indicators of the risk imposed on human health. Our objectives were to use historical, previously unpublished sets of entomological and published clinical data and examine the importance of temperature in contributing to the spreading of the malaria vector *An. hyrcanus*; to differentiate between sites with a specific frequency of WNV occurrence in *Cx. pipiens*, and to assess the impact of increasing UVR and HD on melanoma incidence using the EBU-POM regional model data. A similar approach was recently used in observing the dramatic decline in total flying insect biomass in protected areas in Germany [36].

Despite globalisation trends, researchers have become "closed" in their ever-smaller communication circles which are not limited by state or national borders but by the professional language and way of thinking. Thus, by the end of the 20th century, the scientific community has been faced with problems in communication within its confines. One of the principal reasons why vector-borne diseases (VBD) are so difficult to predict is the complex interaction of multiple factors (vector, host, pathogen, environment including short-term weather patterns and long-term climate change) in space and time [37,38]. Only groups from multiple sectors that communicate and work together on specific aspects of VBD systems will be able to answer the most exciting and pressing problems in the field [37]. Authors of this paper started collaboration in 2003 comparing the climates of the foci of WNV circulation in USA (California Central Valley) and Europe (Bucharest area) with VPS. As compared climates showed quite similar patterns, colleagues from public health and veterinary joined the initial group of meteorologists and medical entomologists. With the idea to better draw upon the resources and insights of the various sectors

we designed and implemented research and programmes to achieve better outcomes in the control of zoonoses (diseases that can spread between animals and humans, e.g., WNV disease). This led us to the following achievements: (i) the first detection of WNV in horses in Serbia in 2009 [39]; (ii) the first detection of WNV in mosquitoes in Serbia in 2010 [26]; (iii) the first detection of WNV in wild birds in Serbia in 2012 [40]; (iv) development and implementation of the national programme of WNV surveillance in mosquito, bird and horse population [8], combined with human surveillance in VPS from 2014; (v) increased visibility to ECDC, EFSA and WHO; (vi) the first detection of imported dengue human case in Serbia in 2016 [41]; and (vii) development and implementation of "One Health" programme in VPS from 2018. We are quite sure that much less would have been achieved without multidisciplinary communication and collaboration initiated in 2003, and this paper would not have been compiled.

The temperature trend over the period of observations used in this study and for the future time horizon following A1B scenario obtained with the EBU-POM regional climate model is within the multi-model ensemble (MME) spread of regional climate models with the similar configuration used in the ENSEMBLES project [42]. For the period 2001-2030 the temperature change for the region of interest in the EBU-POM integration is 0.75 °C concerning the period 1961-1990 and for the same period ENSEMBLES MME spread range is 0.5-1.5 °C [43]. Following this finding, other results presented in this paper that relay on temperature change, can be seen as an estimate that will be within uncertainty related to the future temperature projection.

Mosquito vectors. Until the end of the 20th century, northern Serbia was considered the northern limit for the distribution of *An. hyrcanus* in Europe. The first detection in Serbia dates from 1979 [44] from the north part of VPS. We found it in the central part of the Province in 1985 [25] and since then have been noticing its continued spread. Several records north from Vojvodina, in Slovakia in 2004 [45], the Czech Republic in 2005 [46], and Austria in 2012 [47] confirm our observation. Due to its exophilic and exophagic behaviour, *An. hyrcanus* has never been considered as the primary vector of malaria in Europe.

Its spread to higher latitudes, combined with the changes in human behaviour (increased outdoor leisure activities, the mobility of humans, number of seasonal workers in the field, number of migrants in Europe), might increase its vector capacity. The similar northern spread of population distribution range that was registered for *Anopheles maculipennis s.s.* in Russia [48], and *Culiseta longiareolata* in southern (in 2012 [49]) and northern (in 2013 [50]) Austria might well represent the tendency described with our model.

The latest illustration of similar changes is the finding of *Uranotaenia unguiculata*, a thermophilic mosquito species frequently occurring in the Mediterranean basin, in northern Germany, some 300km north of the previous northern limit [51].

During the period 2001–2030 in which the spread and population growth of *An. hyrcanus* is expectedthe intensity of UVR is likely to increase in the VPS (Fig 4a). Positive trends which are present in our observations might indicate that the findings on the negative influence of UVR and blue-light radiation (this radiation has a wavelength between approximately 380 nm and 500 nm; it has a very short wavelength, and so produces a higher amount of energy) on adult mosquitoes under laboratory conditions [52,53] could not be simply translated to the field. This experimental evidence does not mean unavoidably that the blue light radiation has a significant influence on adult mosquitoes in field conditions since they can actively escape over-exposure to radiation.

The WNV transmission cycle involves mosquito vectors and birds, but equines and humans are also susceptible to infection [54,55]. Although WNV infections have been described in a wide variety of vertebrates, birds are the main natural reservoir. Hundreds of wild and domestic avian species have been described as susceptible to WNV infection, but many of these showed only subclinical infection [56]. In horses, WNV infection is also frequently clinically unapparent, but around 10% of cases develop neurological disorders with up to 50% mortality rates [55,57]. An increasing number of severe outbreaks in horses have been reported in Europe in the past decade, including a large one that took place in northeast Italy in 2008 involving 251 stables with 794 cases and five deaths [57].

From the first detection of WNV in 8 out of 81 found dead wild birds in Serbia [40], each year WNV

nucleic acid was detected in found dead or captured wild birds during summertime [8]. Serological testing of horses sampled during 2009-2010 showed that 12% of 349 horses from the northern part of Serbia had neutralising WNV antibodies [39]. After that, each year horse WNV cases were detected by the positive serological response (IgG and IgM antibody seroconverted horses) [8] or as a clinical manifestation of West Nile neuroinvasive disease [58].

A positive association between WNV disease and temperature was already reported in Europe [15,59] where climate and landscape were critical predictors of WNV disease outbreaks [60]. Our focus was not on the number of human WNV cases, but the suitability of sites/microhabitats with different air temperatures for WNV circulation in mosquitoes, which may well correspond to a higher risk of transmission. We found that sites with higher T_{oa} and T_a were characterized with the higher frequency of WNV presence in mosquitoes. Clustering of cases with an incidence higher than one in six years coincided with an area of a significant grouping of mosquito, bird, horse, and human cases in 2014 and 2015 [9] (Fig 5). This is in concurrence with Tran et al. [61] and Marcantonio et al. [60], who found that average summer temperatures are positively correlated with WNV human incidence. It seems that temperature in semi-urban areas is an essential environmental factor influencing WNV circulation (landscape suitability for reservoir host and mosquito vector, host availability, and precipitation/water availability are somewhat similar in investigated semi-urban areas of VPS), as it affects both mosquito vector abundance [9] and virus replication. Prediction of a two-fold increase in virus incidence for each 0.5 °C increase in T_{oa} indicates but does not necessarily mean, that the number of human cases could increase too. Therefore, our findings support the statement that climate change is likely to intensify the re-emergence of WNV in Europe [62].

Fig 5. Frequency of sampling of WNV infected mosquitoes (1–5 times, coloured numbers) during the period 2010–2016, superimposed over a cluster of mosquito, bird, horse, and human WNV cases in (a) 2014 and (b) 2015 (modified after Petrić et al. [9]).

Melanoma incidence and UVR. According to World Health Organization (WHO) (1992) and many other authors [63,64], exposure to UVR radiation is considered to be a major etiological factor for all three forms of melanoma (i) basal cell carcinoma (BCC), (ii) squamous cell carcinoma (SCC), and (iii) malignant melanoma (MM). We found the correlation between MM and climate changes impact on UVR and also the number of HD. We see the impact as a modification of ambient UVR through influences on other variables such as clouds and aerosols. However, that impact might be more pronounced through the impact of changes in outdoor ambient temperature which will influence people's behaviour and increase the time they spend outdoors, i.e. exposure to both higher UVR and higher temperatures [17]. Experiments with animals clearly show that increased temperatures enhance UVR-induced melanoma compared to the room temperature. In an intriguing study, van der Leun [65] postulated that long-term elevation of temperature by 2 °C, as a consequence of climate change, would increase the carcinogenic effects of UVR by 10%. Our results for the UVR in the VPS are generally similar to the ones obtained by Malinović-Milićević et al. [66] and Malinović-Milićević and Radovanović [67], who reported the following changes: (1) the reduction of yearly averages for the total ozone of 3.44% and 3.21%, and (2) increase in erythemal UVR dose of 6.9% and 9.7% for the periods 1990–1999 and 2000–2009, respectively.

According to Jovanović et al. [7], the incidence rate of MM cancer in VPS for the period 1985–2004 is higher than in central Serbia and is comparable with the majority of the central European countries as the highest melanoma incidence rate in the world [68]. However, most studies do not deal more quantitatively with the relationship between UVR doses and exposure during HD days, and as it has been stated above, the cumulative exposure to sunlight is probably the most critical risk factor for MM and SCC cancers, while BCC is more associated with intensive short-term exposure [69]. Thus, the increasing trend in the number of melanoma incidence in the VPS for the period 1985–2004 (Fig 4d) can be ascribed to (i) the increase in the number of HD days for about 55% and (ii) the increase in cumulative values of UVR doses for the period 1985–2030. In a cohort study, Wu et al. [70] considered the impact of long-term UV radiation flux on skin cancer risk. Comparing with participants in the lowest quintile of cumulative UV

radiation flux in adulthood, they found that participants in the highest quintile had multivariable-adjusted risks (cumulative incidence). According to Vandenbroucke and Pearce [71], some studies where cumulative incidence is used can over-represent the trends.

From a statistical point of view, the linear regression model for modelling the cumulative incidence of melanoma versus the difference of the cumulative UVR doses for hot and warm days (Fig 4d) is acceptable. Parameters are statistically highly significant (r = 0.9712 and p = 0.000003) while analysis of residual distribution shows a good agreement with the normal distribution (Shapiro-Wilk test, W = 0.9608, p = 0.7952).

We hope that our results will indicate the importance of long-term monitoring/surveillance programs for providing crucial data to evidence the ongoing biological alteration triggered by climate change. Nonetheless, it is difficult to say how broadly our data represent the trends elsewhere. We believe that the specificity of the observations offers a unique window into the state of some of the planet's pressing threats to human health. Also, in the case where humans are exposed to UVR, due to the nature of their work (the VPS is an exclusively agricultural area), it is necessary to (i) establish a broader network for UVR measurements and warning centres and (ii) increase the awareness of the melanoma as a result of increased amount of UVR.

Acknowledgements

For the collection of samples over the period 1985-2005, we warmly thank Petar Igrić, Dragan Aradski, Dragan Dondur, Vid Srdić, Mihaela Kavran, Slavica Vaselek, and Dubravka Pudar. The works of Dušan Petrić and Mina Petrić were done under the frame of EurNegVec COST Action TD1303.

References

1. Costello A, Abbas M, Allen A, Ball S, Bell S, Bellamy R, et al. Managing the health effects of climate change: lancet and University College London Institute for Global Health Commission. The Lancet. 2009;373: 1693–1733.

2. Mihailović DT, Lalić B, Drešković N, Mimić G, Djurdjević V, Jančić M. Climate change effects on crop yields in Serbia and related shifts of Köppen climate zones under the SRES-A1B and SRES-A2. Int J Climatol. 2015;35: 3320–3334. doi:10.1002/joc.4209

3. King LJ, Anderson LR, Blackmore CG, Blackwell MJ, Lautner EA, Marcus LC, et al. Executive summary of the AVMA One Health Initiative Task Force report. J Am Vet Med Assoc. 2008;233: 259–261. doi:10.2460/javma.233.2.259

4. Health - European External Action Service [Internet]. [cited 29 Mar 2019]. Available: https://eeas.europa.eu/headquarters/headquarters-homepage/2348/health_ku

5. One World, One Health: OIE - World Organisation for Animal Health [Internet]. [cited 27 Mar 2019]. Available: http://www.oie.int/en/for-the-media/editorials/detail/article/one-world-one-health/

6. Kjellstrom T, McMichael AJ. Climate change threats to population health and well-being: the imperative of protective solutions that will last. Glob Health Action. 2013;6. doi:10.3402/gha.v6i0.20816

7. Jovanović M, M Bogdanović G, Mijatović Jovanović V, Jeremić P, Ac Nikolić E. Analysis of cutaneous melanoma in the province of Vojvodina. J BUON Off J Balk Union Oncol. 2009;14: 441–6.

8. Petrović T, Šekler M, Petrić D, Lazić S, Debeljak Z, Vidanović D, et al. Methodology and results of integrated WNV surveillance programmes in Serbia. PLOS ONE. 2018;13: e0195439. doi:10.1371/journal.pone.0195439

9. Petrić D, Petrović T, Hrnjaković Cvjetković I, Zgomba M, Milošević V, Lazić G, et al. West Nile virus “circulation” in Vojvodina, Serbia: Mosquito, bird, horse and human surveillance. Mol Cell Probes. 2017;31: 28–36. doi:10.1016/j.mcp.2016.10.011

10. Zeller H, Lenglet A, Bortel WV. West Nile virus: the need to strengthen preparedness in Europe. Eurosurveillance. 2010;15: 19647. doi:10.2807/ese.15.34.19647-en

11. World Health Organization, World Malaria Report 2018. Geneva: The Organization; 2018.

12. Vakali A, Patsoula E, Spanakos G, Danis K, Vassalou E, Tegos N, et al. Malaria in Greece, 1975 to 2010. Euro Surveill Bull Eur Sur Mal Transm Eur Commun Dis Bull. 2012;17.

13. Epidemiological update: West Nile virus transmission season in Europe, 2018 [Internet]. [cited 09 Sep 2019]. Available from: https://ecdc.europa.eu/en/news-events/epidemiological-update-west-nile-virus-transmission-season-europe-2018

14. Wilson N, Lush D, Baker MG. Meteorological and climate change themes at the 2010 International Conference on Emerging Infectious Diseases. Euro Surveill Bull Eur Sur Mal Transm Eur Commun Dis Bull. 2010;15.

15. Calzolari M, Pautasso A, Montarsi F, Albieri A, Bellini R, Bonilauri P, et al. West Nile virus surveillance in 2013 via mosquito screening in northern Italy and the influence of weather on virus circulation. PLoS One. 2015;10: e0140915.

16. Apalla Z, Lallas A, Sotiriou E, Lazaridou E, Ioannides D. Epidemiological trends in skin cancer.

Dermatol Pract Concept. 2017;7: 1.

17. Kerr JB, Seckmeyer G. Surface ultraviolet radiation: past and future Scientific Assessment of Ozone Depletion 2002. Geneva; 2003. Report No.: 47.

18. Summary for Policy makers. In: Nakićenović N, Swart R, editors. Special Report on Emissions Scenarios. Cambridge: Cambridge University Press - Published for the Intergovernmental Panel on Climate Change. 2000. pp. 1-21.

19. Kottek M, Grieser J, Beck C, Rudolf B, Rubel F. World Map of the Köppen-Geiger climate classification updated. Meteorologische Zeitschrift. 2006;15(3):259–263.

20. Đurđević V, Rajković B. Development of the EBU-POM coupled regional climate model and results from climate change experiments. In: Mihailovic DT, Lalic B, editors. Advances in environmental modeling and measurements. New York, USA: Nova Science Publishers Inc; 2012. pp. 23–32.

21. Roeckner E, Bäuml G, Bonaventura L. The atmospheric general circulation model ECHAM5. Part I: Model description Report. Max Planck Institute for Meteorology; 2003 p. 127. Report No.: 349.

22. Jungclaus JH, Keenlyside N, Botzet M, Haak H, Luo J-J, Latif M, et al. Ocean Circulation and Tropical Variability in the Coupled Model ECHAM5/MPI-OM. J Clim. 2006;19: 3952–3972. doi:10.1175/JCLI3827.1

23. Malinovic-Milicevic S, Mihailovic DT, Lalic B, Dreskovic N. Thermal environment and UV-B radiation indices in the Vojvodina region, Serbia. Clim Res. 2013;57: 111–121. doi:10.3354/cr01163

24. Srdić Ž, Zgomba M, Petrić D. Les moustiques (Dip.Culicidae) et la demoustication en Vojvodina, Yugoslavia. IV Congrès sur la protection de la santé humaine et des cultures en milieu tropical. Marseille, France; 1986. pp. 489–495.

25. Petrić D. Seasonal and daily activity of adult mosquitoes (Diptera, Culicidae) in Vojvodina Province. Ph.D. Thesis, University of Novi Sad. 1989.

26. Petrić D, Hrnjaković-Cvjetković I, Radovanov J, Cvjetković D, Jerant-Patić V, Milošević V, et al. West Nile virus surveillance in humans and mosquitoes and detection of cell fusing agent virus in Vojvodina Province (Serbia). HealthMED 2012;6(2):462–68.

27. Becker N, Petrić D, Zgomba M, Boase C, Madon M, Dahl C, et al. Mosquitoes and their control. 2nd ed. Berlin Heidelberg: Springer-Verlag; 2010.

28. Mihailović DT, Mimić G, Arsenić I. Climate Predictions: The Chaos and Complexity in Climate Models. In: Advances in Meteorology [Internet]. 2014 [cited 27 Mar 2019]. doi:10.1155/2014/878249

29. Richman JS, Moorman JR. Physiological time-series analysis using approximate entropy and sample entropy. Am J Physiol Heart Circ Physiol. 2000;278: H2039-2049. doi:10.1152/ajpheart.2000.278.6.H2039

30. Krzic A, Tosic I, Djurdjevic V, Veljovic K, Rajkovic B. Changes in some indices over Serbia

according to the SRES A1B and A2 scenarios. Climate Research. 2011;49: 73-86.

31. Cavicchia L, Scoccimarro E, Gualdi S, Marson P, Ahrens B, Berthou S, et al. Mediterranean extreme precipitation: a multi-model assessment. Clim. Dyn. 2016. doi: 10.1007/s00382-016-3245-x

32. Dell' Aquila A, Mariotti A, Bastin S, Calmanti S, Cavicchia L, Deque M, et al. Evaluation of simulated decadal variations over the Euro-Mediterranean region from ENSEMBLES to Med-CORDEX. Clim. Dyn. 2016. doi:10.1007/s00382-016-3143-2

33. R Core Team. R: A Language and Environment for Statistical Computing [Internet]. Vienna: R Foundation for Statistical Computing; 2016. Available: http://www.R-project.org/

34. HS Global Inc. Eviews [Internet]. CA, USA: HS Global Inc.; 2016. Available: http://www.eviews.com

35. TIBCO Software Inc. Statistica [Internet]. CA, USA: TIBCO Software Inc.; Available: https://docs.tibco.com/products/tibco-statistica-13-3-0

36. Hallmann CA, Sorg M, Jongejans E, Siepel H, Hofland N, Schwan H, et al. More than 75 percent decline over 27 years in total flying insect biomass in protected areas. PloS One. 2017;12: e0185809.

37. Moore CG. Interdisciplinary research in the ecology of vector-borne diseases: Opportunities and needs. Journal of Vector Ecology 2008;33(2):218–224.

38. Zimmerman B. Engaging with Complexity: Thrive! A Plan for a Healthier Nova Scotia. 2014; [e-print] Available from: https://thrive.novascotia.ca/sites/default/files/Thrive-Summit-2014-Brenda-Zimmerman-En.pdf.

39. Lupulović D, Martin-Acebes MA, Lazić S, Alonso-Padilla J, Blazquez AB, Escribano-Romero E, et al. First serological evidence of West Nile virus activity in horses in Serbia. Vector Borne Zoonotic Dis. 2011;11(9):1303–5.40.

40. Petrović T, Blazquez AB, Lupulović D, Lazić G, Escribano-Romero E, Fabijan D, et al. Monitoring West Nile virus (WNV) infection in wild birds in Serbia during 2012: First isolation and characterisation of WNV strains from Serbia. Eurosurveillance. 2013;18(44):1–8.

41. Petrović V, Turkulov V, Ilić S, Milošević V, Petrović M, Petrić D, et al. First report of imported case of dengue fever in Republic of Serbia. Vol. 14, Travel Medicine and Infectious Disease. 2016. p. 60–1.

42. van der Linden P and Mitchell JFB, editors. ENSEMBLES Climate Change and its Impacts. Summary of research and results from the ENSEMBLES project. Exeter: Met Office Hadley Centre;. 2009.:

43. Ministry of Environmental Protection of the Republic of Serbia. Second National Communication of the Republic of Serbia under the United Nations Framework Convention on Climate Change [Internet]. Belgrade: The Ministry; 2017 Aug. 162p [cited 2019 Sep 10]. Available from: http://www.klimatskepromene.rs/wp-content/uploads/2017/09/SNC_eng.pdf

44. Adamović Ž. Distribution and abundance of anopheline mosquitoes (Diptera, Culicidae) in Vojvodina, Serbia. Acta Entomol Jugosl. 1979;15: 82.

45. Halgoš J, Benková I. First record of *Anopheles hyrcanus* (Diptera: Culicidae) from Slovakia. Biológia. 2004;59: 68.

46. Votýpka J, Šeblová V, Rádrová J. Spread of the West Nile virus vector *Culex modestus* and the potential malaria vector *Anopheles hyrcanus* in central Europe. J Vector Ecol. 2008;33: 269–277. doi:10.3376/1081-1710-33.2.269

47. Lebl K, Brugger K, Rubel F. Predicting *Culex pipiens*/*restuans* population dynamics by interval lagged weather data. Parasit Vectors. 2013;6: 129. doi:10.1186/1756-3305-6-129

48. Novikov YM, Vaulin OV. Expansion of *Anopheles maculipennis* s.s. (Diptera: Culicidae) to northeastern Europe and northwestern Asia: Causes and Consequences. Parasit Vectors. 2014;7: 389. doi:10.1186/1756-3305-7-389

49. Seidel B, Nowotny N, Duh D, Indra A, Hufnagl P, Allerberger F. First records of the thermophilic mosquito *Culiseta longiareolata* (Macquart, 1838) in Austria, 2012, and in Slovenia, 2013. J Eur Mosq Control Assoc. 2013;31: 17–20.

50. Zittra C, Waringer J, Werblow A, Melaun C, Fuehrer H-P. Reconfirmation of *Culiseta* (*Allotheobaldia*) *longiareolata* (Macquart 1838) (Diptera: Culicidae) in Austria. The first sequence-confirmed findings in northeastern Austria. Acta ZooBot Austria. 2014;150: 17–24.

51. Tippelt L, Walther D, Kampen H. The thermophilic mosquito species *Uranotaenia unguiculata* Edwards, 1913 (Diptera: Culicidae) moves north in Germany. Parasitol Res. 2017;116: 3437–3440. doi:10.1007/s00436-017-5652-2

52. Riordan DF. Effects of ultraviolet radiation on adults of *Aedes aegypti* (L.) (Diptera : Culicidae). Mosq News. 1969;29. Available: https://www.cabdirect.org/cabdirect/abstract/19711000053

53. Hori M, Shibuya K, Sato M, Saito Y. Lethal effects of short-wavelength visible light on insects. Sci Rep. 2014;4: 7383. doi:10.1038/srep07383

54. Kramer L, Li J, Shi PY. West Nile virus. Lancet Neurol. 2007;6:171–181.

55. Blitvich BJ. Transmission dynamics and changing epidemiology of West Nile virus. Anim Health Res Rev. 2008;9:71–86.

56. Komar N. West Nile virus: epidemiology and ecology in North America. Adv Vir Res. 2003;6:185–234.

57. Calistri P, Giovannini A, Hubalek Z, Ionescu A, Monaco F, Savini G et al. Epidemiology of West Nile in Europe and in the Mediterranean basin. Open Virol J. 2010;4(1):29–37.

58. Medić S, Lazić S, Petrović T, Petrić D, Samojlović M, Lazić G et al. Evidence of the first clinical case of equine neuroinvasive West Nile Disease in Serbia, 2018. Acta veterinaria 2019;69(1):123–130.

59. Paz S, Malkinson D, Green MS, Tsioni G, Papa A, Danis K, et al. Permissive Summer

Temperatures of the 2010 European West Nile Fever Upsurge. PLOS ONE. 2013;8: e56398. doi:10.1371/journal.pone.0056398

60. Marcantonio M, Rizzoli A, Metz M, Rosà R, Marini G, Chadwick E, et al. Identifying the Environmental Conditions Favouring West Nile Virus Outbreaks in Europe. Gourbiere S, editor. PLOS ONE. 2015;10: e0121158. doi:10.1371/journal.pone.0121158

61. Tran A, Sudre B, Paz S, Rossi M, Desbrosse A, Chevalier V, et al. Environmental predictors of West Nile fever risk in Europe. Int J Health Geogr. 2014;13: 26. doi:10.1186/1476-072X-13-26

62. Paz S. Climate change impacts on West Nile virus transmission in a global context. Philos Trans R Soc B Biol Sci. 2015;370: 20130561–20130561. doi:10.1098/rstb.2013.0561

63. Narayanan DL, Saladi RN, Fox JL. Ultraviolet radiation and skin cancer. Int J Dermatol. 2010;49: 978–986. doi:10.1111/j.1365-4632.2010.04474.x

64. Lacy K, Alwan W. Skin cancer. Medicine (Baltimore). 2013;41: 402–405. doi:10.1016/j.mpmed.2013.04.008

65. Leun JC van der. Uv-Carcinogenesis. Photochem Photobiol. 1984;39: 861–868. doi:10.1111/j.1751-1097.1984.tb08872.x

66. Malinovic-Milicevic S, Mihailovic DT, Radovanovic MM. Reconstruction of the erythemal UV radiation data in Novi Sad (Serbia) using the NEOPLANTA parametric model. Theor Appl Climatol. 2015;121: 131–138. doi:10.1007/s00704-014-1223-y

67. Malinović-Milićević S and Radovanović M 2016 UV radiation and heat wives in Vojvodina (Book 87) (Belgrade: Serbian Academy of Sciences and Arts, Institute of Geography „Jovan Cvijić") p ISBN.

68. de Vries E, Tyczynski J, Parkin MD. Cutaneous malignant melanoma in Europe. Eur Netw Cancer Regist Int Agency Res Cancer. 2003;ENCR Cancer Fact Sheets no.4 1-4.

69. Demers AA, Nugent Z, Mihalcioiu C, Wiseman MC, Kliewer EV. Trends of nonmelanoma skin cancer from 1960 through 2000 in a Canadian population. J Am Acad Dermatol. 2005;53: 320–328.

70. Wu S, Han J, Laden F, Qureshi AA. Long-term ultraviolet flux, other potential risk factors, and skin cancer risk: a cohort study. Cancer epidemiology, biomarkers and prevention: a publication of the American Association for Cancer Research, cosponsored by the American Society of Preventive Oncology. 2014;*23*(6):1080–1089.

71. Vandenbroucke JP, Pearce N. Incidence rates in dynamic populations. Int J Epidemiol. 2012;41(5):1472–1479.

Supporting information

S1 Table. Overview of dry-ice trap samples sizes. For each year, the number of locations sampled,

the number of location re-sampled, and total number of samples are presented. Exposure time at the trap locations was similar (14 ± 2h).

S2 Table. Number of the total trap nights, positive trap nights and *Anopheles hyrcanus* specimens sampled at 54 selected sites in the Vojvodina Province, Serbia during the years 1985-86, 2004-5 and 2014-15.

S3 Table. Frequency of sampling of WNV infected mosquitoes (1 – 5 times) in the Vojvodina Province, Serbia, during the period 2010-2016.

Figure

Click here to access/download;Figure;Fig1.tif

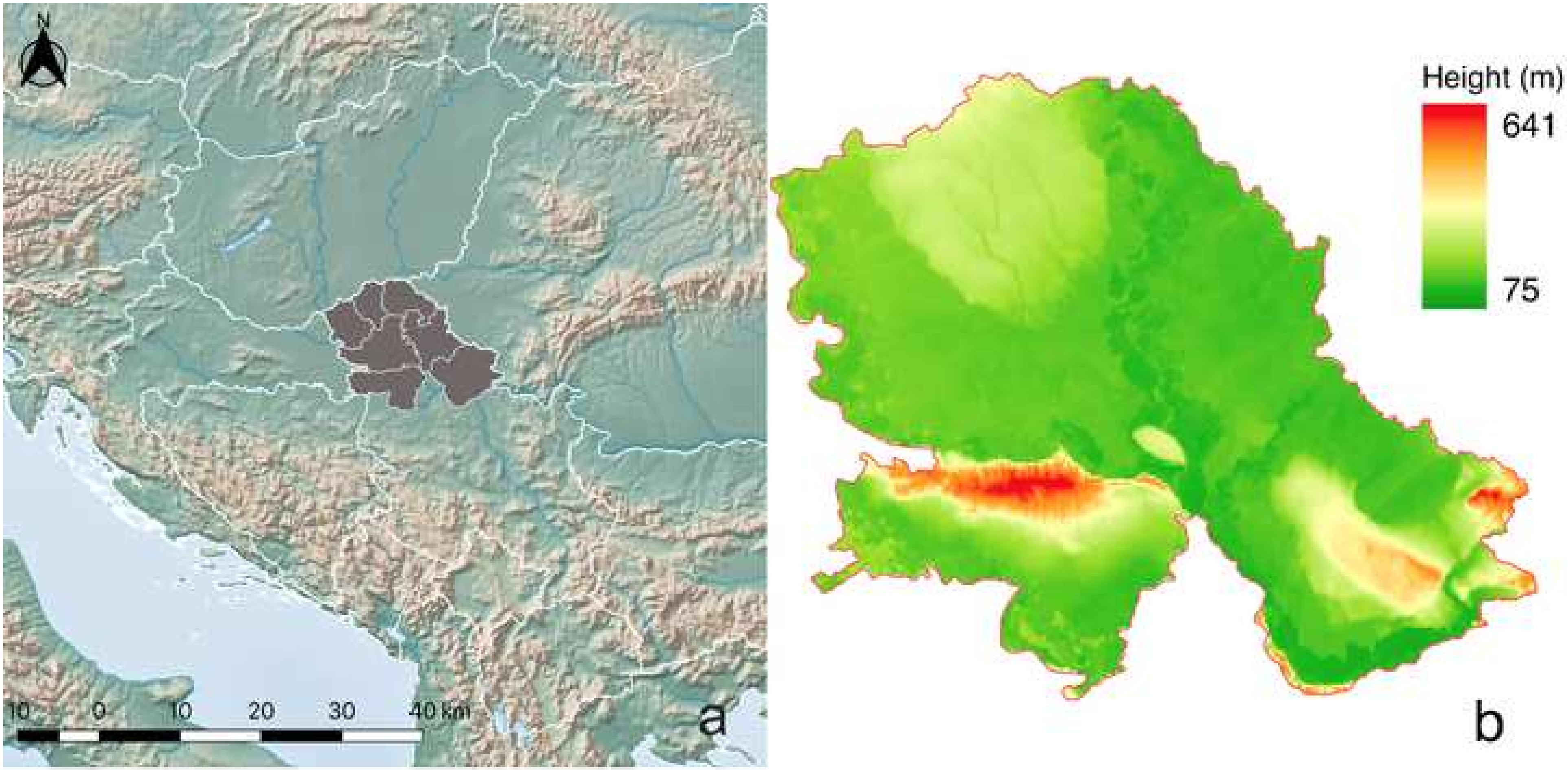

Click here to access/download;Figure;Fig2.tif

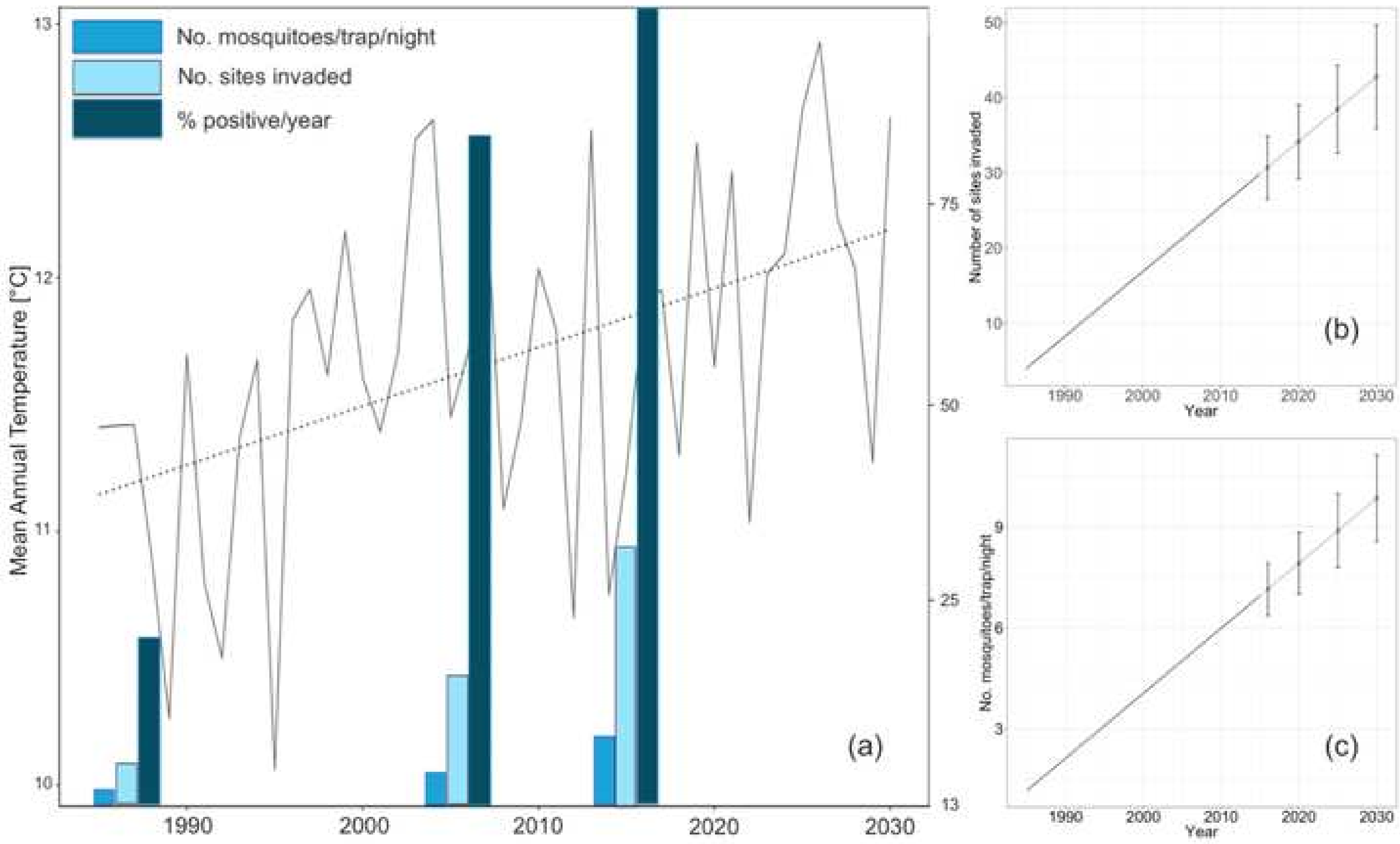

Click here to access/download;Figure;Fig3.tif

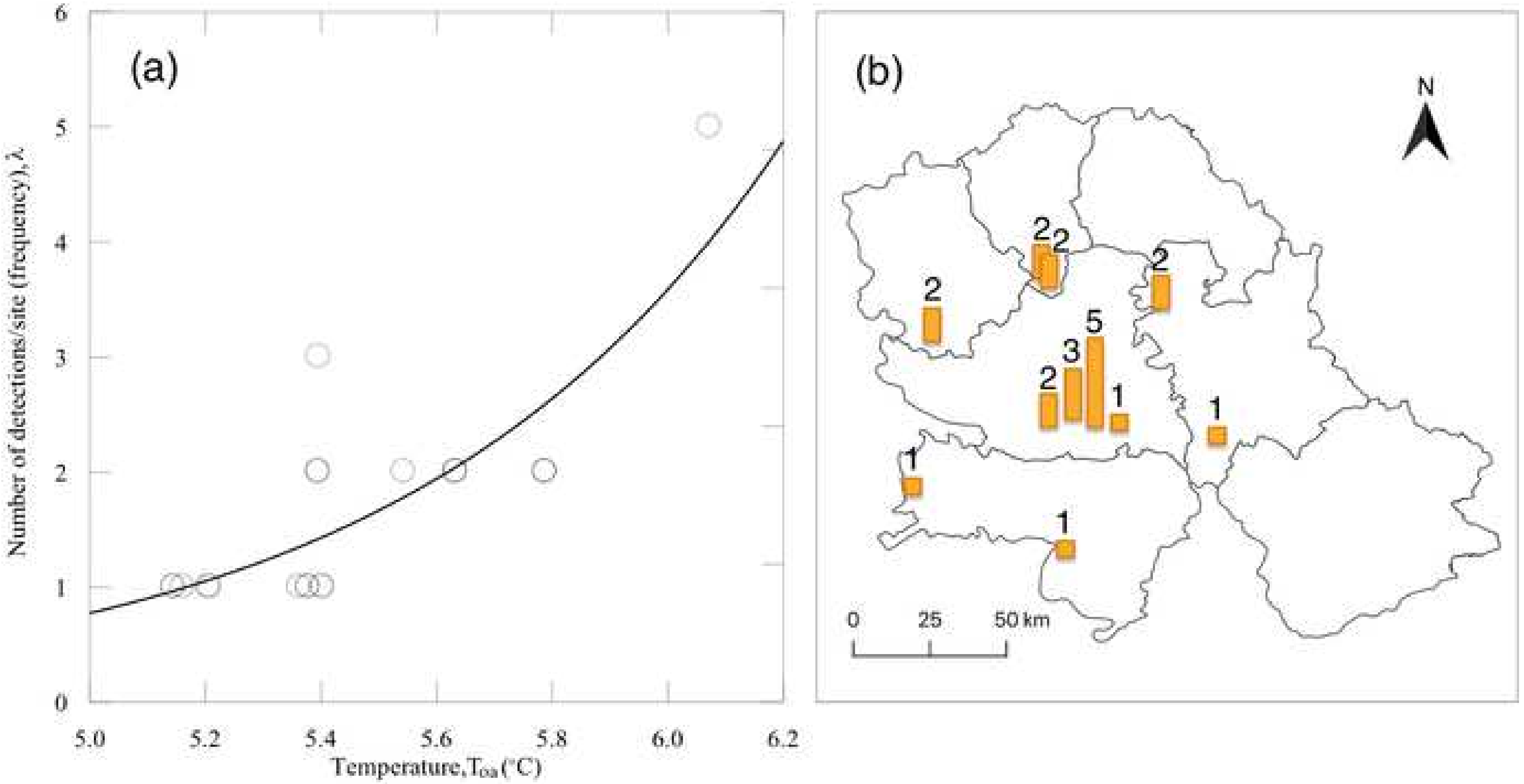

Figure

Click here to access/download;Figure;Fig4.tif

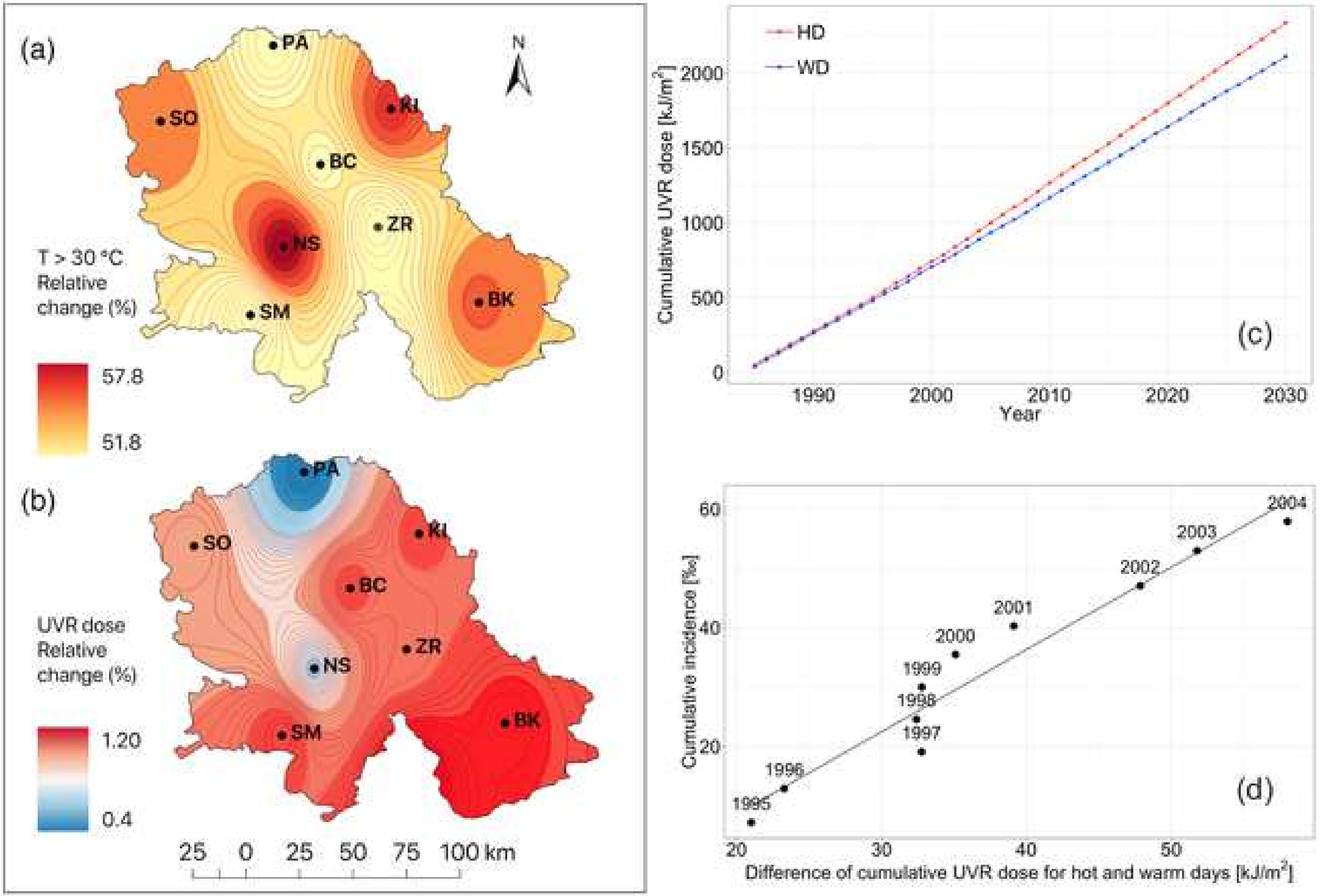

Figure

Click here to access/download;Figure;Fig5.tif

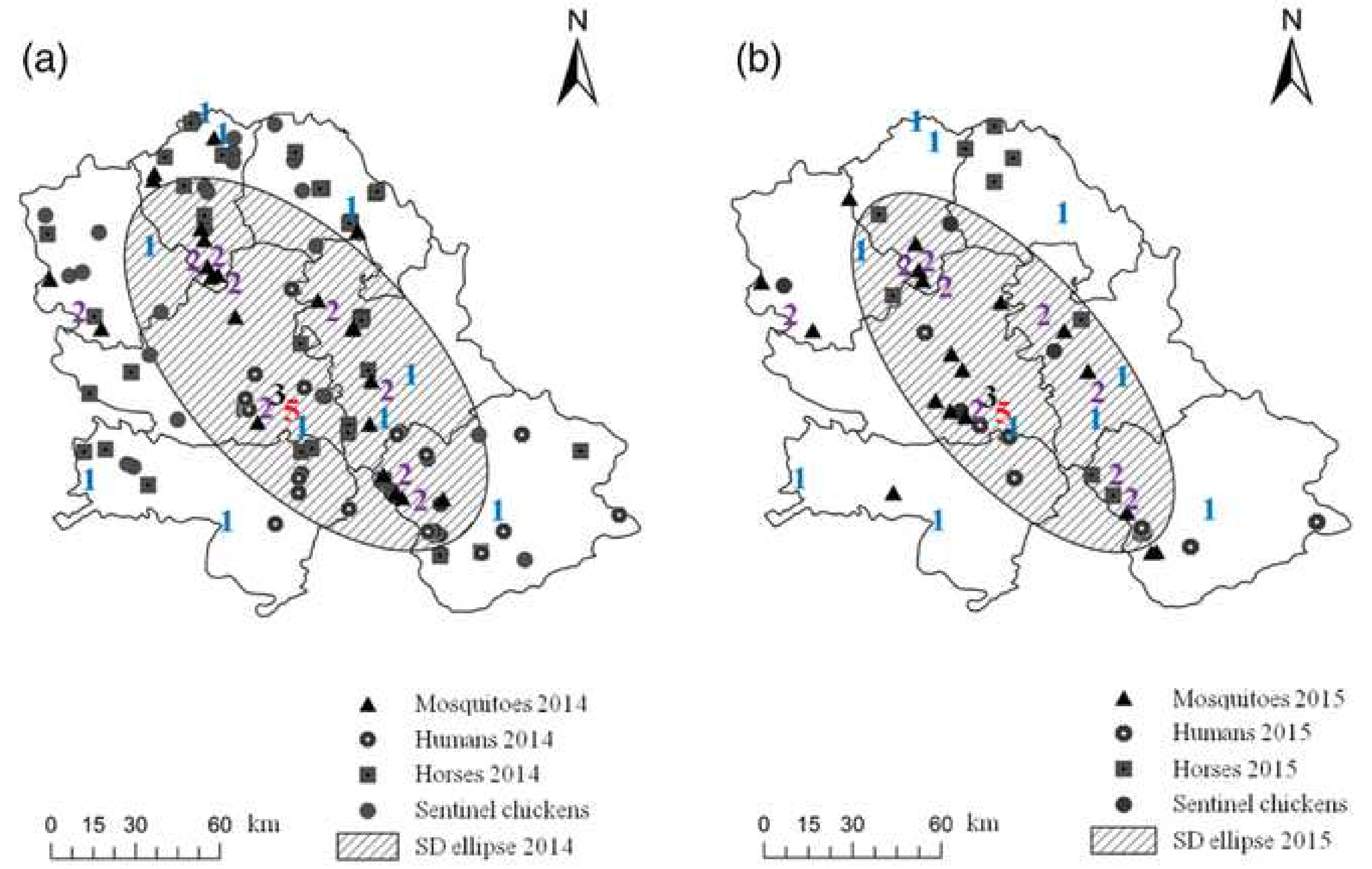

Supporting Information 1

Click here to access/download
Supporting Information
S1_Table.xlsx

Supporting Information 2

Click here to access/download
Supporting Information
S2_Table.xlsx

Click here to access/download
Supporting Information
S3_Table.docx

Assessment of climate change impact on the malaria vectorsvector *Anopheles hyrcanus*, West Nile disease, and incidence of melanoma in the Vojvodina Province (Serbia) using data from a regional climate model

Dragutin T. Mihailović[1], Dušan Petrić[2,*], Tamaš Petrović[3], Ivana Hrnjaković-Cvjetković[4,5], Vladimir Đurđević[6]Djurdjevic[6], Emilija Nikolić-Đorić[7], Ilija Arsenić[1], Mina Petrić[8,9,10,*], Gordan Mimić[11], Aleksandra Ignjatović-Ćupina[2]

[1] Department of Field and Vegetable Crops, Faculty of Agriculture, University of Novi Sad, Novi Sad, Serbia

[2] Department of Plant and Environment Protection, Faculty of Agriculture, University of Novi Sad, Novi Sad, Serbia

[3] Department for virology, Scientific Veterinary Institute “Novi Sad”, Novi Sad, Serbia

[4] Institute of Public Health of Vojvodina, Novi Sad, Serbia

[5] Faculty of Medicine, University of Novi Sad, Novi Sad, Serbia

[6] Institute of Meteorology, Faculty of Physics, University of Belgrade, Belgrade, Serbia

[7] Department of Agricultural Economics, Faculty of Agriculture, University of Novi Sad, Novi Sad, Serbia

[8] Avia-GIS NV, Zoersel, Belgium

[9] Department of Physics, Faculty of Sciences, University of Novi Sad, Novi Sad, Serbia

[10] Department of Physics and Astronomy, Faculty of Sciences, University of Gent, Gent, Belgium

[11] BioSense Institute, University of Novi Sad, Novi Sad, Serbia

* Corresponding authors E-mail: ~~guto@polj.uns.ac.rs~~

mpetric@avia-gis.com (MP)

dusanp@polj.uns.ac.rs~~Received xxxxxx~~

~~Accepted for publication xxxxxx~~

~~Published xxxxxx~~

(DP)

Abstract

Motivated by the One Health paradigm, we found the expected changes in temperature and UV radiation (UVR) to be a common trigger for enhancing the risk that viruses, vectors<u>,</u> and diseases pose to human and animal health. We ~~compare~~<u>compared</u> data from the mosquito field collections and medical studies with regional climate model projections to examine the impact of climate change on the <u>spreading of one malaria vector, the</u> circulation of West Nile virus (WNV), ~~the spreading of the malaria vector~~ and the incidence of melanoma. We analysed data obtained from ten selected years of ~~standardized~~<u>standardised</u> mosquito vector sampling with 219 unique location-year combinations, and 10~~-~~ years of melanoma incidence. Trends in the observed data were compared to the climatic variables obtained by the coupled regional Eta Belgrade University and Princeton Ocean Model for the period 1961~~–~~–2015 using the A1B scenario, and the expected changes up to 2030 were presented.<u> Spreading and relative abundance of *Anopheles hyrcanus* was positively correlated with the trend of the mean annual temperature. We anticipated a nearly twofold increase in the number of invaded sites up to 2030.</u>~~-~~ The frequency of WNV detections in *Culex pipiens* was significantly correlated to overwintering temperature averages and seasonal relative humidity at the sampling sites. Regression model projects a twofold increase in the incidence of WNV positive ~~*Culex*~~<u>*Cx*</u>. *pipiens* for a rise of 0.5 °C in overwintering $T_{October–April}$ temperatures.~~ Spreading and relative abundance of *Anopheles hyrcanus* was positively correlated with the trend of the mean annual temperature. We anticipated a nearly twofold increase in the number of invaded sites up to 2030.~~ The projected increase of 56% in the number of days with $T_{max} \geq 30$ ºC (<u>Hot Days - </u>HD) and UVR doses (up to 1.2%) corresponds to an increasing trend in melanoma incidence. Simulations of the Pannonian countries climate anticipate warmer and drier conditions with possible dominance of temperature and number of HD over other ecological factors. These signal the importance of monitoring the changes to the preparedness of mitigating the risk<u> of vector-borne diseases and melanoma</u>.

Introduction

Climate change is referred to as "the biggest global health threat of the 21st century" [1]. The analysis of outputs from all general circulation models (GCM) ~~suggest~~suggests that the countries of the Pannonian Plain, including Serbia, are facing significant impacts of climate change, affecting all aspects of human life [2]. The authors of the manuscript (meteorology, entomology, veterinary medicine, and public health experts), have been working together since 2003, promoting the idea of multisectoral collaboration before the One Health Concept was officially inaugurated in the USA in 2007 [3], and endorsed by the European Union (EU) [4] as well as prominent organizations such as the World Health Organization~~,~~ (WHO), Food and Agriculture Organization (FAO), and the World Organization for Animal Health (OIE) in 2018 [5].

In this paper, the authors collected and analysed observed data ~~collected~~ over~~ a period of~~ 31 years and related a subset to outputs from a Regional Climate Model (RCM). Vector-borne diseases and melanoma are significant climate-driven threats for which risk sources can be clearly defined [6]. Moreover, both present progressively growing environmental threats to the animal as well as human health in the countries of the Pannonian Plane~~.~~ [7,8,9].

The biology and distribution of mosquito vectors and their capacity to transmit mosquito-borne diseases are dependent on many factors such as global trade and travel, ~~urbanization~~urbanisation, habitat destruction, pesticide application, host density, and climate.~~ *Culex pipiens* and~~ *Anopheles hyrcanus* and *Culex pipiens* are mosquito species that are vectors of malaria and West Nile virus (WNV) disease~~ and malaria~~, respectively, the two most detrimental vector-borne diseases worldwide [~~7]. In 2018, Serbia was the second European country (after Italy) most affected by WNV disease (415 reported cases with 35 fatal outcomes [8]).~~10,11]. Malaria was eradicated from Serbia and other Balkan states during the last century. However, the spreading of its vectors (*Anopheles* mosquitoes) and the re-emergence of the disease in Greece [~~9] pose a threat to South East and Central Europe once again.~~12] pose a threat to the South East and Central Europe once again. In 2018, Serbia was the second European country (after Italy) most affected by WNV disease (415 reported cases with 35 fatal outcomes). In Europe, the total number

of reported human autochthonous WNV infections in 2018 (n=2,083) exceeded, by far, the total number from the previous seven years (n=1,832). During the same transmission season, outbreaks of West Nile fever among equids increased by 30% compared to the number of outbreaks in 2017. In total, 285 outbreaks among equids were reported by the EU Member States in 2018 [13]. Current evidence suggests that inter-annual and inter-decadal climate variability have a direct influence on the epidemiology of vector-borne diseases, with temperature and relative humidity as the principal abiotic factors influencing the life-cycles of the mosquito vector, the pathogen, the host and the interactions between them [~~10,11~~14,15].

Melanoma is a malignant disease that has experienced a significant increase in incidence during the last few decades all over the world [~~12]. The climate~~16]. Climate change impact on melanoma should be considered as a synergy of changes in UV radiation (UVR) due to stratospheric ozone depletion and the long-term increase of air temperature leading to more prolonged exposure of individuals to UVR doses and consequently to a higher risk of melanoma [~~13]. The melanoma~~17]. Melanoma mortality in the Vojvodina Province (northern Serbia) (VPS) ~~in~~within the period 1985~~-~~–2004 shows an evident increase, placing it amongst the most vulnerable regions in the world. Thus, Jovanović et al. [~~14~~7] estimated and made the list of mortality rates from malignant melanoma for males (age-~~standardized~~standardised rate/100,000) in Europe (39 countries) for the year 2000, using European Network of Cancer Registries (ENCR) data. This list shows that the VPS is among the top eleven states (six of them ~~having~~have parts in the Pannonian Plane) listed as the most endangered.

In this study, devoted to revealing the potential impact of climate change on animal and human health, we ~~compare~~compared a considerable amount of previously unpublished ecological data obtained from the field and clinical surveys with climate change projections for the VPS, which is representative of the Central European low-altitude areas with a human-dominated landscape (Fig 1). We examined the effects of temperature on the spread and relative abundance of the malaria vector *An. hyrcanus* and the "microclimate" differentiation between sites with a specific frequency of WNV occurrence in *Cx. pipiens* ~~and effects of temperature on the spread and relative abundance of the malaria vector *An. hyrcanus*.~~. We

also evaluated the impact of climate change on melanoma incidence as a synergy of changes in UVR doses and the long-term increase in the number of hot days (HD), with daily maximum temperature ~~>~~≥ 30°C using the Eta Belgrade University and Princeton Ocean Model (EBU-POM) regional model data.

Fig 1. (a) Location of the Vojvodina Province (Serbia) in Europe and (b) altitude map~~.~~ (Map data copyrighted OpenStreetMap contributors and available from https://www.openstreetmap.org)

Materials and Methods

For the assessment of the climate change and the impact of UVR doses, we used the climatic variables obtained by the coupled regional EBU-POM model for the historical period 1961~~-2000 and the period 2001-2030 using the SRES-A1B scenario.~~ -2000 and the period 2001-2030 according to the A1B scenario defined in Special Report on Emissions Scenarios (SRES) [18], from now on SRES-A1B. SRES scenarios, which defined future global greenhouse gases emissions, were extensively used in the Intergovernmental Panel on Climate Change (IPCC) Third, and Fourth Assessment Reports. The main storyline behind the A1B scenario is rapid economic growth, followed by a significant increase in greenhouse gases concentrations in the future. In the Fifth Assessment Report (AR5), the Representative Concentration Pathway (RCP) is introduced, which are possible future concentration pathways without any storyline behind them. Comparing SRES-A1B and RCPs in terms of the greenhouse gases concentrations, at the end of this century SRES-A1B is the closest to RCP6.0, but for the time horizon used in this study, up to 2030, the difference between any SRES or RCPs are relatively small.

Study area and climate

The VPS is situated in the northern part of Serbia and the southern part of the Pannonian lowland (18°51′–21°33′E, 44°37′–46°11′N and 75–641 m.a.s.l. (with the Fruška Gora Mountain in the south) as it is seen in ~~Fig~~Figs 1a and ~~Fig~~ 1b). This region is the essential food production area in Serbia with a total

surface area of 21,500 km^2 and a population of about 2 million. This region has a continental climate, with elements of a sub-humid and warm climate (Cfwbx" according to Köppen classification~~).~~ [19]).

Models and formula used

The global and regional climate model

For climate simulations in this study, we used results of the EBU-POM model runs for the SRES-A1B scenario integrated over the period 2001–2030 [~~15~~20]. The EBU-POM is a two-way, coupled RCM. The atmospheric part is the Eta/National Centres for Environmental Prediction (NCEP) limited area model (resolution 0.25° × 0.25° on 32 vertical levels; centred at 41.5° N, 15° E, with boundaries at ±19.9° W–E and ±13.0° S–N), while the oceanic part is the POM (resolution 0.20° × 0.20° on 21 vertical levels). The driving global circulation model (GCM) was the ECHAM5 model [~~16~~21] coupled with the Max Planck Institute Ocean Model (MPI-OM) [~~17~~22]. More details about model integrations and performed bias correction for VPS can be found in the paper by Mihailović et al. [2]. The POM model was set over the Mediterranean Sea without the Black Sea; for other open seas, the sea surface temperature from the GCM was used as a bottom boundary condition.

Empirical ~~formulae~~formula

For calculating the daily doses of UVR, i.e. UVRD in the study area sites we have used the following empirical formula $UVRD = 0.002507 \times G_d - 5.985$ (kJ/m2) derived by Malinović-Milićević et al. [~~18~~23], where G_d is the daily sum of ~~the~~ global solar radiation.

Environmental sampling

Mosquito vectors

We used ~~standardized~~standardised protocols to measure mosquito presence/absence, density, and infestation by WNV. Data ~~are~~were extracted from dry ice-baited trap samples, collected over 31 years at 166 different sites (745 sampled locations, S1 Table) in the VPS, to infer on the trends of local vector

status and virus circulation in mosquitoes. In all years mosquitoes were sampled from May to September, with different spatial intensity and time-frequency governed by the scale and scope of different research projects. For comparison with climate variables, we extracted data obtained in 10 years (1985––6, 2004––2005 and 2010––2015) for which a standardizedstandardised surveillance protocol was in place. These periods have the highest number of particular location-year combinations (S1-S3 Tables).

Samples were collected by two different types of dry-ice baited suction traps. During 1985 and 1986 [19,2024,25] by the miniature CDC light trap (CDC) and for 2004 and 2015 by the NS2 trap (our own design of dry ice-baited suction trap without light). Both traps were operating without a light source (incandescent light proved not to be attractive/repellent for most mosquito species inhabiting the VPS [20].25]). The CDC trap has a 3––5 times stronger suction power (operated by a 9 V battery) than NS2 (operated by 3 x 1.2 V batteries), meaning that the increase in density of species observed after 1986 could not be attributed to the change of the type of trap. Traps were operated from the afternoon until the morning of the next day (one trap night), with different periodicity. The specific location of the trap at each site was chosen by experienced entomologists to stabilizestabilise variation of the collected data.

We used three parameters to indicate *An. hyrcanus* spread and population growth in the period 1985-2015: i) the ratio of positive to total mosquito samplings per year; ii) the number of sites invaded (positive places where it was looked for, but was not found in the preceding sampling period, and the number of sites where was observed in both periods, i.e. established); and iii) the average number of specimens sampled in one trap during single sampling period from the afternoon of the starting day to the morning of the next day (FigFigs 2a and Fig 2c). Here, we used data from 1,073 mosquito samples (1985-–6, 2004-–5 and 2014-–5), obtained at 54 location over 6six years (142 unique location-year combinations) (S2 Table).

Fig 2. (a) The CRCMregional climate model EBU-POM projection of the mean annual air temperature (T_a) for the period 1985––2030 and: i) number of specimens sampled in one trap during single sampling period (light blue columns); ii) the number of sites invaded by *An. hyrcanus*

(redlight blue columns); and iii) relative number of positive samplings per year (greendark blue columns), (b) projected increase in the number of sites invaded by *An. hyrcanus* (the period 2001––2030 ±S.E.), and (c) projected increase in the number of the specimens sampled in one trap during single sampling period (2001––2030 ±S.E.).

For *Cx. pipiens*, the period starting with the first detection of WNV in mosquitoes in Serbia, in 2010, (Petrić et al. [21]) [26], to 2015 was considered. To investigate the impact of microclimate on the complex interaction between *Cx. pipiens* and WNV, we used the following climatic parameters from the EBU-POM model outputs (covering the period 2006–2015) for 11 sites (GPS coordinates – S3 Table) in the VPS with different histories of WNV circulation: (i) mean annual temperature (T_a); (ii) overwintering temperature (T_{oa}) for the period October – April; and (iii) seasonal temperature (T_{ms}) and relative humidity (R_{ms}) for the period May – September. For these sites, we examined the correlation between the frequency of WNV detections in *Cx. pipiens* at each site (from 2010to 2015) and the corresponding period averages of climate time series for the same site. For detection of WNV, specimens were sampled, anaesthetizedanaesthetised by dry ice, identified to species level [2227] on dry ice--cooled paper, pooled according to date, location, sex, and species, transported on dry ice to the laboratory, and stored at -70 °C before virus detection. Pool size did not exceed 50 mosquito specimens per pool. Virus detectionMosquito pools were tested for WNV RNA presence by TaqMan-based one-step reverse transcription real-time PCR (RT-qPCR) that amplified both lineage 1 and 2 strains. Viral RNA was performedextracted using the commercial *ISOLATE II RNA Mini Kit* (Bioline, The Netherlands) according to proceduresthe manufacturer's instruction. One-step RT-qPCR was conducted using the commercial kit *RNA UltraSense™ One-Step qRT-PCR System* (Life Technologies Corporation) with the primers and probe that targeted the nucleocapsid protein C gene regions of WNV, as described in Petrovéby Petrović et al. [238]. We analyzedanalysed the yearly occurrence of the WNV positive *Cx. pipiens* mosquitoes sampled by dry ice-baited traps in the years 2010––2015 across 7766 unique location-year combinations (S3 Table). Only traps positioned exactlyprecisely at the same spot over the entire six-

year period are considered for analysis. Numbers allocated to different places (Fig 3) indicate the number of years in the period 2010~~-~~–2015 in which WNV was detected in sampled *Cx. pipiens* mosquitoes; e.g. 5 indicates that WNV positive *Cx. pipiens* were detected in five out of the six years in the samples collected from the same spot.

Fig 3. (a) Dependence of frequencies (λ) of WNV positive *Culex pipiens* detections at the same site on overwintering temperatures (Toa); (b) Frequency of sampling of WNV infected mosquitoes (1 – 5 times) during six years (bars and numbers) in NUTS3 (Nomenclature of Territorial Units for Statistics) units of the Vojvodina Province, Serbia.

Melanoma incidence and UVR

~~Indicators for a ten-year period 1995 - 2004 of melanoma incidence in women and men based on the data obtained from the Cancer Registry of Vojvodina following the methodology of Jovanović et al. [14] were used for the analysis.~~

In the analysis we have used two indicators: (i) melanoma incidence rate that is a measure of the number of new cases ("incidence") per unit of time ("rate") and (ii) cumulative incidence ("incidence proportion" that measures the number of new cases per person in the population over a defined period of time – often called risk or proportion). Melanoma incidence rate (per 100,000 people) for ten years 1995 - 2004 was based on the data obtained from the paper by Jovanović et al. [7]. From these data, we calculated the cumulative incidence. We have used the model simulation to study the expected impact of climate change on UVR exposure of human skin for nine sites in VPS [PA (Palić), SO (Sombor), KI (Kikinda), NS (Novi Sad), BC (Bečej, ZR (Zrenjanin), SM (Sremska Mitrovica), BK (Bantaski Karlovac), and BG (Beograd)]. Firstly, we calculated daily UVR doses (UVRD) from global radiation model outputs using the empirical formula for the nine sites for the period April-September, and then we found the relative change R(UVRD) of those doses as R(UVRD) = (UVRD-UVRD$_k$) / UVRD$_k$ where UVRD$_k$ is the dose for

1961–1990 reference period, while the UVRD is calculated for the period 2001–2030.

Statistics

We considered the papers by Mihailović et al. [2,~~24~~28] in which Kolmogorov complexity measures (Kolmogorov complexity (KC), Kolmogorov complexity spectrum KC spectrum) and the highest value of the KC spectrum (KCM)) and sample entropy ~~[25~~(SE) [29] were used to quantify the regularity and complexity of air temperature and precipitation time series, obtained by the EBU-POM model, representing both deterministic chaos and stochastic processes.~~ Then, the obtained results were compared with the same information measures using data taken from daily meteorological reports of the Republic Hydrometeorological Service of Serbia.~~ We considered the complexity of the EBU-POM model using the observed and modelled time series of temperature and precipitation. We computed the KC spectrum, KC, KCM and SE values for temperature and precipitation. The calculations were performed for the entire time interval 1961–1990: (i) on a daily basis with a size of N =10958 samples for temperature and (ii) on a monthly basis with a size N =360 for the precipitation. The simulated time series of temperature and precipitation were obtained by the EBU-POM model for the given period. The observed time series of temperature and precipitations for two stations: Sombor (SO) (88 m.a.s.l.) and Novi Sad (NS) (84 m.a.s.l.) in the considered area, were taken from daily meteorological reports of the Republic Hydrometeorological Service of Serbia. For both sites, the modelled complexity is lower than the observed one, but with the reliability which is in the interval values allowed by the information measures (KC, KCM, and SE) [30,31,32]. These findings mean that the models with a KC (and KCM) complexity lower than the measured time series complexity cannot always reconstruct some of the structures contained in the observed data. However, it does not mean that outputs from EBU-POM model do not correctly simulate climate elements since both sites values indicate the absence of stochastic influences, providing reliable projections of the climate elements.

For *An. hyrcanus*, the temperature trend was evaluated by the Mann-Kendall test using the R statistical package [~~26~~33]. Field observed values on species distribution and density for the period 1985~~-~~–2015 and

forecasts of the numbers of sites invaded and specimens sampled for the period 2016—–2030 based on linear trend were obtained by the Eviews 9.5 software [2734]. For *Cx. pipiens*, the relationship between yearly frequency of WNV detection in mosquitoes, air temperature, and relative humidity (derived from the climate model) was estimated using Spearman's Rank-Order Correlation and a Poisson regression model (Statistica 13 [28]).35]). For melanoma, the linear regression model was used for modelling the cumulative incidence of melanoma versus the difference of the cumulative UVR doses for hot and warm days (Fig 4d). Analysis of residual distribution was done by Shapiro-Wilk test (Statistica 13 [35])

Results

Mosquito vectors. FigureFig 2a shows an evident linear trend of the mean annual temperature T_a for the period 1985—–2030 (r = 0.467; p = 0.001; τ = 0.328) calculated from the EBU-POM regional model outputs for 29 representative sites in the VPS. All parameters that were chosen for the evaluation of the spread and population increase of *An. hyrcanus* were positively, but to a different extent, correlated to the time argument (periods in which sampling was performed since the beginning of monitoring in 1985) indicating a monotonic trend. The increase of parameters follows the trend of T_a (Fig 2a). The strongest correlation was found for the increase in the ratio of positive samplings (r = 0.986; p <= 0.001000307; τ = 0.828), followed by the number of mosquitoes per trap night (r = 0.919; p <= 0.05009639; τ = 0.733),), and the number of sites invaded (r = 0.889; p <= 0.0501766; τ = 0.6). By 2030 we anticipate a further increase in numbers of invaded sites and adult females sampled, by 1.71 and 1.27 fold, respectively (FigFigs 2b and Fig 2c).

Spearman rank-order correlation To investigate the impact of microclimate on the complex interaction between *Cx. pipiens* and WNV, we used the following climatic parameters from the EBU-POM model outputs (covering the period 2006-2015) for 11 sites (GPS coordinates – S3 Table) in the VPS with different histories of WNV circulation: (i) mean annual temperature (T_a); (ii) overwintering temperature (T_{oa}) for the period October – April; and (iii) seasonal temperature (T_{ms}) and relative humidity (R_{ms}) for

~~the period May – September. For these sites, we examined the correlation~~ between the frequency of WNV detections in *Cx. pipiens* at ~~each site (from 2010, when WNV was detected for the first time in the mosquito vector *Cx. pipiens* in Serbia, to 2015)~~ 11 sites and the corresponding ~~period averages~~ mean values of climate time series ~~for the same site. Spearman rank order correlation of the mean values~~ -was the highest for T_{oa} (r = 0.755; p ~~<~~= 0.~~05~~00008), then for T_a (r = 0.616; p ~~<~~= 0.~~05~~00294), R_{ms} (r = 0.499; p ~~<~~= 0.~~05)~~02119), and T_{ms} (r = 0.477; p ~~<~~= 0.~~05). Figure~~02856). Fig 3a depicts the Poisson regression model for the dependence of a number of detections per site (frequency - λ) on T_{oa}, which is ~~highly~~ significant (p ~~<~~= 0.~~05~~01393). The output of the model ($ln\lambda = -7.923 + 1.533 \times T_{OA}$) indicates that for an increase of 0.5 °C in T_{oa} (presuming that all other factors needed for the circulation of WNV are kept constant), a twofold increase in the incidence of WNV positive *Cx. pipiens* could be projected. ~~Figure~~Fig 3b depicts that most of the sites with the high frequency of WNV occurrence (≥ 2) were distributed along the northwest-southeast axis of the VPS.

Melanoma incidence and UVR doses. ~~We have used the model simulation to study the expected impact of climate change on UVR exposure of human skin for nine sites in VPS [PA (Palić), SO (Sombor), KI (Kikinda), NS (Novi Sad), BC (Bečej, ZR (Zrenjanin), SM (Sremska Mitrovica), BK (Bantaski Karlovac) and BG (Beograd)]. Firstly, we calculated daily UVR doses (UVRD) from global radiation model outputs using the empirical formula for the seven aforementioned counties for the period April-September, and then we found the relative change R(UVRD) of those doses as R(UVRD) = (UVRD-UVRD~~$_k$~~)/ UVRD~~$_k$ ~~where UVRD~~$_k$ ~~is the dose for the 1961–1990 reference period, while the UVRD is calculated for the period 2001-2030. Figure~~ Fig 4b shows the positive relative change of UVRD, remarkably covering an eastern, southern, western, and partly central area of VPS. Specifically, the projected increase is twofold going from the west and northwest (0.60%) towards the east and southeast where it reaches values of about 1.20%. The EBU-POM model (for nine sites) shows a significant expected increase of 56% in the number of HD days in the VPS (Fig 4a), compared to the period 1961~~–~~–1990. Additionally, we observed

a decrease of 1.1% in the number of days with maximum air temperature ~~higher than~~Tmax ≥ 25~~°~~ °C (~~warm days~~Warm Days - WD~~-~~). This prolongs the exposure of outdoor working adults to UVR and thus leads to ~~the~~an increase in melanoma risk. This risk becomes even more significant because of the increase in cumulative values of UVR doses (Fig 4c). ~~Figure~~Fig 4d depicts the cumulative incidence of melanoma for the period 1985~~–~~–2004 with an increasing monotonic trend (r = 0.~~970,~~9712 and p ~~<~~≤ 0.~~001~~000003).

Fig 4. (a) Relative change of hot days (HD) ~~(a)~~ and (b) UVR radiation doses [R (UVRD)] ~~(b)~~ for the period 2001~~-~~–2030 compared to the period 1961~~-~~–1990~~,~~; (c) cumulative values of mean UVR doses for the period 1985~~-~~–2030 (averaged for seven sites: PA, SO, BC, KI, NS, ZR, SM, and BK) under the SRES-A1B scenario ~~[~~(for WD and HD days~~]~~); and (d) cumulative incidence of melanoma for the period 1995~~–~~–2004 ~~(ja bih izabrao ovakav zapis)~~ in the Vojvodina Province, Serbia.

Discussion

Here we presented an intriguing comparison of the impact of climate change on complex systems including mosquito vectors, pathogens, and ~~humans~~melanoma, which are all indicators of the risk imposed on human health. Our objectives were to use historical, previously unpublished sets of entomological and published clinical data and examine the importance of temperature in contributing to the spreading of the malaria vector *An. hyrcanus*; to differentiate between sites with a specific frequency of WNV occurrence in *Cx. pipiens*, and to assess the impact of increasing UVR and HD on melanoma incidence using the EBU-POM regional model data. A similar approach was recently used in observing the dramatic decline in total flying insect biomass in protected areas in Germany [~~29~~36].

Despite globalisation trends, researchers have become "closed" in their ever-smaller communication circles which are not limited by state or national borders but by the professional language and way of thinking. Thus, by the end of the 20th century, the scientific community has been faced with problems in

communication within its confines. One of the principal reasons why vector-borne diseases (VBD) are so difficult to predict is the complex interaction of multiple factors (vector, host, pathogen, environment including short-term weather patterns and long-term climate change) in space and time [37,38]. Only groups from multiple sectors that communicate and work together on specific aspects of VBD systems will be able to answer the most exciting and pressing problems in the field [37]. Authors of this paper started collaboration in 2003 comparing the climates of the foci of WNV circulation in USA (California Central Valley) and Europe (Bucharest area) with VPS. As compared climates showed quite similar patterns, colleagues from public health and veterinary joined the initial group of meteorologists and medical entomologists. With the idea to better draw upon the resources and insights of the various sectors we designed and implemented research and programmes to achieve better outcomes in the control of zoonoses (diseases that can spread between animals and humans, e.g., WNV disease). This led us to the following achievements: (i) the first detection of WNV in horses in Serbia in 2009 [39]; (ii) the first detection of WNV in mosquitoes in Serbia in 2010 [26]; (iii) the first detection of WNV in wild birds in Serbia in 2012 [40]; (iv) development and implementation of the national programme of WNV surveillance in mosquito, bird and horse population [8], combined with human surveillance in VPS from 2014; (v) increased visibility to ECDC, EFSA and WHO; (vi) the first detection of imported dengue human case in Serbia in 2016 [41]; and (vii) development and implementation of "One Health" programme in VPS from 2018. We are quite sure that much less would have been achieved without multidisciplinary communication and collaboration initiated in 2003, and this paper would not have been compiled.

The temperature trend over the period of observations used in this study and for the future time horizon following A1B scenario obtained with the EBU-POM regional climate model is within the multi-model ensemble (MME) spread of regional climate models with the similar configuration used in the ENSEMBLES project [42]. For the period 2001-2030 the temperature change for the region of interest in the EBU-POM integration is 0.75 °C concerning the period 1961-1990 and for the same period ENSEMBLES MME spread range is 0.5-1.5 °C [43]. Following this finding, other results presented in

this paper that relay on temperature change, can be seen as an estimate that will be within uncertainty related to the future temperature projection.

Mosquito vectors. Until the end of the 20th century, northern Serbia was considered the northern limit for the distribution of *An. hyrcanus* in Europe. The first detection in Serbia dates from 1979 [~~30~~44] from the north part of VPS. We found it in the central part of the Province in 1985 [25] and since then have been noticing its continued spread. ~~The several~~Several records north from Vojvodina, in Slovakia in 2004 [~~31~~45], the Czech Republic in 2005 [~~32~~46], and Austria in 2012 [~~33~~47] confirm our observation. Due to its exophilic and exophagic behaviour, *An. hyrcanus* has never been considered as the primary vector of malaria in Europe. Its spread to higher latitudes, combined with the changes in human behaviour (increased outdoor leisure activities, the mobility of humans, number of seasonal workers in the field, number of migrants in Europe), might ~~elevate~~increase its vector capacity. The similar northern spread of population distribution range that was registered for *Anopheles maculipennis s.s.* in Russia [~~34~~48], and *Culiseta longiareolata* in southern (in 2012~~; [35~~ [49]) and northern (in 2013 [~~36~~50]) Austria might well represent the tendency described with our model.

The latest illustration of similar changes is the finding of *Uranotaenia unguiculata*, a thermophilic mosquito species frequently occurring in the Mediterranean basin, in northern Germany, some ~~300 km~~300km north of the previous northern limit [~~37~~51].

During the period 2001~~-~~–2030 in which the spread and population growth of *An. hyrcanus* is ~~expected, the~~expectedthe intensity of UVR is likely to increase in the VPS (Fig 4a). ~~Let us note, that the positive~~ Positive trends which are ~~already~~ present in our observations might indicate that the findings ~~supporting~~on the negative influence of UVR and blue-light radiation (this radiation has a wavelength between approximately 380 nm and 500 nm; it has a very short wavelength, and so produces a higher amount of energy) on adult mosquitoes under laboratory conditions [~~38,39].~~52,53] could not be simply translated to the field. This experimental evidence does not mean unavoidably that the blue light radiation has a significant influence on adult mosquitoes in field conditions~~,~~ since they ~~are able to~~can actively escape over-exposure to radiation.

The WNV transmission cycle involves mosquito vectors and birds, but equines and humans are also susceptible to infection [54,55]. Although WNV infections have been described in a wide variety of vertebrates, birds are the main natural reservoir. Hundreds of wild and domestic avian species have been described as susceptible to WNV infection, but many of these showed only subclinical infection [56]. In horses, WNV infection is also frequently clinically unapparent, but around 10% of cases develop neurological disorders with up to 50% mortality rates [55,57]. An increasing number of severe outbreaks in horses have been reported in Europe in the past decade, including a large one that took place in northeast Italy in 2008 involving 251 stables with 794 cases and five deaths [57].

From the first detection of WNV in 8 out of 81 found dead wild birds in Serbia [40], each year WNV nucleic acid was detected in found dead or captured wild birds during summertime [8]. Serological testing of horses sampled during 2009-2010 showed that 12% of 349 horses from the northern part of Serbia had neutralising WNV antibodies [39]. After that, each year horse WNV cases were detected by the positive serological response (IgG and IgM antibody seroconverted horses) [8] or as a clinical manifestation of West Nile neuroinvasive disease [58].

A positive association between WNV disease and temperature was already reported in Europe [~~11,40~~15,59] where climate and landscape were critical predictors of WNV disease outbreaks [~~41~~60]. Our focus was not on the number of human WNV cases, but the suitability of sites/microhabitats with different air temperatures for WNV circulation in mosquitoes, which may well correspond to a higher risk of transmission. We found that sites with higher T_{oa} and T_a were characterized with the higher frequency of WNV ~~mosquito incidence rate~~presence in mosquitoes. Clustering of cases with an incidence higher than one in six years coincided with an area of a significant grouping of mosquito, bird, horse, and human cases in 2014 and 2015 ~~(Petrić et al. [42] –~~[9] (Fig 5). This is in concurrence with Tran et al. [~~43~~61] and Marcantonio et al. [~~41~~60], who found that average summer temperatures are positively correlated with WNV human incidence. It seems that temperature in semi-urban areas ~~dominates the other~~is an essential environmental ~~factors~~factor influencing WNV circulation ~~in nature (e.g. ~~(landscape suitability for reservoir host and mosquito vector, host availability, and precipitation/water availability are somewhat

similar in investigated semi-urban areas of VPS), as it ~~is the primary factor affecting~~affects both mosquito vector abundance [9] and virus replication. Prediction of a two-fold increase in virus incidence for each 0.5 °C increase in T_{oa} indicates but does not necessarily mean, that the number of human cases could increase too. Therefore, our findings support the statement that climate change is likely to intensify the re-emergence of WNV in Europe [~~44~~62].

Fig 5. Frequency of sampling of WNV infected mosquitoes (1~~–~~–5 times, coloured numbers) during the period 2010~~-~~–2016, superimposed over a cluster of mosquito, bird, horse, and human WNV cases in (a) 2014 and (b) 2015 (modified after Petrić et al. [~~40~~9]).

Melanoma incidence and UVR. According to World Health Organization (WHO) (1992) and many other authors [~~45,46~~63,64], exposure to UVR radiation is considered to be a major etiological factor for all three forms of melanoma (i) basal cell carcinoma (BCC), (ii) squamous cell carcinoma (SCC~~)~~), and (iii) malignant melanoma (MM). We found the correlation between MM and climate changes impact on UVR and also the number of HD. We see the impact as a modification of ambient UVR through influences on other variables such as clouds and aerosols. However, that impact might be more pronounced through the impact of changes in outdoor ambient temperature which will influence people's behaviour and increase the time they spend outdoors, i.e. exposure to both higher UVR and higher temperatures [~~13~~17]. Experiments with animals clearly show that increased temperatures enhance UVR-induced melanoma compared to the room temperature. In an intriguing study, van der Leun [~~47] speculated~~65] postulated that long-term elevation of temperature by 2 °C, as a consequence of climate change, would increase the carcinogenic effects of UVR by 10%. Our results for the UVR in the VPS are generally similar to the ones obtained by Malinović-Milićević et al. [~~48~~66] and Malinović-Milićević and Radovanović [~~49~~67], who reported the following changes: (1) the reduction of yearly averages for the total ozone of 3.44% and 3.21~~%~~%, and (2) increase in erythemal UVR dose of 6.9% and 9.7% for the periods 1990~~-~~–1999 and 2000~~-~~–2009, respectively.

According to Jovanović et al. [147], the incidence rate of MM cancer in VPS for the period 1985-–2004 is higher than in central Serbia and is comparable with the majority of the central European countries as the highest melanoma incidence rate in the world [5068]. However, most studies do not deal more quantitatively with the relationship between UVR doses and exposure during HD days, and as it has been stated above, the cumulative exposure to sunlight is probably the most critical risk factor for MM and SCC cancers, while BCC is more associated with intensive short-term exposure [5169]. Thus, the increasing trend in the number of melanoma incidence in the VPS for the period 1985-–2004 (Fig 4d) can be ascribed to (1i) the increase in the number of HD days for about 55% and (2ii) the increase in cumulative values of UVR doses for the period 1985-–2030. In a cohort study, Wu et al. [70] considered the impact of long-term UV radiation flux on skin cancer risk. Comparing with participants in the lowest quintile of cumulative UV radiation flux in adulthood, they found that participants in the highest quintile had multivariable-adjusted risks (cumulative incidence). According to Vandenbroucke and Pearce [71], some studies where cumulative incidence is used can over-represent the trends.

From a statistical point of view, the linear regression model for modelingmodelling the cumulative incidence of melanoma versus the difference of the cumulative UVR doses for hot and warm days (Fig 4d) is apparently acceptable. Parameters are statistically highly significant (r = 0.97199712 and p <= 0.001000003) while analysis of residual distribution shows a good agreement with the normal distribution (Shapiro-Wilk test, W = 0.9608, p – p plot) as it is seen in Figure 6.= 0.7952).

Fig 6. Residual distribution versus normal distribution (p-p plot) for regression in Fig 4d.

We hope that our results will indicate the importance of long-term monitoring/surveillance programs for providing crucial data to evidence the ongoing biological alteration triggered by climate change. Nonetheless, it is difficult to say how broadly our data represent the trends elsewhere. We believe that the specificity of the observations offers a unique window into the state of some of the planet's pressing threats to human health. Also, in the case where the humans are exposed to UVR, due to the nature of

their work (the VPS is an exclusively agricultural area), it is necessary to (i) establish a broader network for UVR measurements and warning centres and (ii) increase the awareness of the melanoma as a result of increased amount of UVR.

Acknowledgements

~~This paper was realized as a part of the project "Studying climate change and its influence on the environment: impacts, adaptation and mitigation" (43007) financed by the Ministry of Education and Science of the Republic of Serbia, within the framework of integrated and interdisciplinary research for the period 2011-2017. Historical data for mosquito vectors are the outputs of the projects supported by the Veterinary Directorate, Ministry of Agriculture and Environment Protection, Republic of Serbia, Provincial Secretariat for Science and Technological Development, AP Vojvodina; TR31084 MESRS.~~For the collection of samples over the period 1985-2005, we warmly thank Petar Igrić, Dragan Aradski, Dragan Dondur, Vid Srdić, Mihaela Kavran, Slavica Vaselek, and Dubravka Pudar. The works of Dušan Petrić and Mina Petrić were done under the frame of EurNegVec COST Action TD1303.

References

1. Costello A, Abbas M, Allen A, Ball S, Bell S, Bellamy R, et al. Managing the health effects of climate change: lancet and University College London Institute for Global Health Commission. The Lancet. 2009;373: 1693–1733.

2. Mihailović DT, Lalić B, Drešković N, Mimić G, Djurdjević V, Jančić M. Climate change effects on crop yields in Serbia and related shifts of Köppen climate zones under the SRES-A1B and SRES-A2. Int J Climatol. 2015;35: 3320–3334. doi:10.1002/joc.4209

3. King LJ, Anderson LR, Blackmore CG, Blackwell MJ, Lautner EA, Marcus LC, et al. Executive summary of the AVMA One Health Initiative Task Force report. J Am Vet Med Assoc. 2008;233: 259–261. doi:10.2460/javma.233.2.259

4. Health - European External Action Service [Internet]. [cited 29 Mar 2019]. Available: https://eeas.europa.eu/headquarters/headquarters-homepage/2348/health_ku

5. One World, One Health: OIE - World Organisation for Animal Health [Internet]. [cited 27 Mar 2019]. Available: http://www.oie.int/en/for-the-media/editorials/detail/article/one-world-one-health/

6. Kjellstrom T, McMichael AJ. Climate change threats to population health and well-being: the

imperative of protective solutions that will last. Glob Health Action. 2013;6. doi:10.3402/gha.v6i0.20816

~~7~~7. Jovanović M, M Bogdanović G, Mijatović Jovanović V, Jeremić P, Ac Nikolić E. Analysis of cutaneous melanoma in the province of Vojvodina. J BUON Off J Balk Union Oncol. 2009;14: 441–6.

8. Petrović T, Šekler M, Petrić D, Lazić S, Debeljak Z, Vidanović D, et al. Methodology and results of integrated WNV surveillance programmes in Serbia. PLOS ONE. 2018;13: e0195439. doi:10.1371/journal.pone.0195439

9. Petrić D, Petrović T, Hrnjaković Cvjetković I, Zgomba M, Milošević V, Lazić G, et al. West Nile virus “circulation” in Vojvodina, Serbia: Mosquito, bird, horse and human surveillance. Mol Cell Probes. 2017;31: 28–36. doi:10.1016/j.mcp.2016.10.011

10. Zeller H, Lenglet A, Bortel WV. West Nile virus: the need to strengthen preparedness in Europe. Eurosurveillance. 2010;15: 19647. doi:10.2807/ese.15.34.19647-en

~~8. Weekly updates: 2018 West Nile fever transmission season [Internet]. [cited 27 Mar 2019]. Available: https://ecdc.europa.eu/en/west-nile-fever/surveillance-and-disease-data/disease-data-ecdc~~

~~9~~11. World Health Organization, World Malaria Report 2018. Geneva: The Organization; 2018.

12. Vakali A, Patsoula E, Spanakos G, Danis K, Vassalou E, Tegos N, et al. Malaria in Greece, 1975 to 2010. Euro Surveill Bull Eur Sur Mal Transm Eur Commun Dis Bull. 2012;17.

~~10~~13. Epidemiological update: West Nile virus transmission season in Europe, 2018 [Internet]. [cited 09 Sep 2019]. Available from: https://ecdc.europa.eu/en/news-events/epidemiological-update-west-nile-virus-transmission-season-europe-2018

14. Wilson N, Lush D, Baker MG. Meteorological and climate change themes at the 2010 International Conference on Emerging Infectious Diseases. Euro Surveill Bull Eur Sur Mal Transm Eur Commun Dis Bull. 2010;15.

~~11~~15. Calzolari M, Pautasso A, Montarsi F, Albieri A, Bellini R, Bonilauri P, et al. West Nile virus surveillance in 2013 via mosquito screening in northern Italy and the influence of weather on virus circulation. PLoS One. 2015;10: e0140915.

~~12~~16. Apalla Z, Lallas A, Sotiriou E, Lazaridou E, Ioannides D. Epidemiological trends in skin cancer. Dermatol Pract Concept. 2017;7: 1.

~~13~~17. Kerr JB, Seckmeyer G. Surface ultraviolet radiation: past and future Scientific Assessment of Ozone Depletion 2002. Geneva; 2003. Report No.: 47.

~~14. Jovanović M, M Bogdanović G, Mijatović Jovanović V, Jeremić P, Ac Nikolić E.~~18. Summary for Policy makers. In: Nakićenović N, Swart R, editors. Special Report on Emissions Scenarios. Cambridge: Cambridge University Press - Published for the Intergovernmental Panel on Climate Change. 2000. pp. 1-21.

19. Kottek M, Grieser J, Beck C, Rudolf B, Rubel F. World Map of the Köppen-Geiger climate classification updated. Meteorologische Zeitschrift. 2006;15(3):259–263.

20 Analysis of cutaneous melanoma in the province of Vojvodina. J BUON Off J Balk Union Oncol. 2009;14: 441–6.

15. Đurđević V, Rajković B. Development of the EBU-POM coupled regional climate model and results from climate change experiments. In: Mihailovic DT, Lalic B, editors. Advances in environmental modeling and measurements. New York, USA: Nova Science Publishers Inc; 2012. pp. 23–32.

1621. Roeckner E, Bäuml G, Bonaventura L. The atmospheric general circulation model ECHAM5. Part I: Model description Report. Max Planck Institute for Meteorology; 2003 p. 127. Report No.: 349.

1722. Jungclaus JH, Keenlyside N, Botzet M, Haak H, Luo J-J, Latif M, et al. Ocean Circulation and Tropical Variability in the Coupled Model ECHAM5/MPI-OM. J Clim. 2006;19: 3952–3972. doi:10.1175/JCLI3827.1

1823. Malinovic-Milicevic S, Mihailovic DT, Lalic B, Dreskovic N. Thermal environment and UV-B radiation indices in the Vojvodina region, Serbia. Clim Res. 2013;57: 111–121. doi:10.3354/cr01163

1924. Srdić Ž, Zgomba M, Petrić D. Les moustiques (Dip.Culicidae) et la demoustication en Vojvodina, Yugoslavia. IV CongressCongrès sur la protection de la santehumaineetsanté humaine et des cultures on mileuen milieu tropical. Marseille, France; 1986. pp. 489–495.

2025. Petrić D. Seasonal and daily activity of adult mosquitoes (Diptera, Culicidae) in Vojvodina Province. Ph.D. Thesis, University of Novi Sad. 1989.

21. Petrić D, Zgomba M, Bellini R, Becker N. Surveillance of Mosquito Populations: A Key Element to Understanding the Spread of Invasive Vector Species and Vector-Borne Diseases in Europe. In: Mihailovic DT, editor. Essays of Fundamental and Applied Environmental Topics. New York, USA: Nova Science Publishers; 2012. pp. 193–224.

2226. Petrić D, Hrnjaković-Cvjetković I, Radovanov J, Cvjetković D, Jerant-Patić V, Milošević V, et al. West Nile virus surveillance in humans and mosquitoes and detection of cell fusing agent virus in Vojvodina Province (Serbia). HealthMED 2012;6(2):462–68.

27. Becker N, Petrić D, Zgomba M, Boase C, Lane J, ZgombaMadon M, Dahl C, et al. Mosquitoes and their control. 2nd ed. Berlin Heidelberg: Springer-Verlag; 2010.-

23. Petrović T, Šekler M, Petrić D, Lazić S, Debeljak Z, Vidanović D, et al. Methodology and results of integrated WNV surveillance programmes in Serbia. PLOS ONE. 2018;13: e0195439. doi:10.1371/journal.pone.0195439

2428. Mihailović DT, Mimić G, Arsenić I. Climate Predictions: The Chaos and Complexity in Climate Models. In: Advances in Meteorology [Internet]. 2014 [cited 27 Mar 2019]. doi:10.1155/2014/878249

2529. Richman JS, Moorman JR. Physiological time-series analysis using approximate entropy and sample entropy. Am J Physiol Heart Circ Physiol. 2000;278: H2039-2049. doi:10.1152/ajpheart.2000.278.6.H2039

2630. Krzic A, Tosic I, Djurdjevic V, Veljovic K, Rajkovic B. Changes in some indices over Serbia according to the SRES A1B and A2 scenarios. Climate Research. 2011;49: 73-86.

31. Cavicchia L, Scoccimarro E, Gualdi S, Marson P, Ahrens B, Berthou S, et al. Mediterranean extreme precipitation: a multi-model assessment. Clim. Dyn. 2016. doi: 10.1007/s00382-016-3245-x

32. Dell' Aquila A, Mariotti A, Bastin S, Calmanti S, Cavicchia L, Deque M, et al. Evaluation of simulated decadal variations over the Euro-Mediterranean region from ENSEMBLES to Med-CORDEX. Clim. Dyn. 2016. doi:10.1007/s00382-016-3143-2

33. R Core Team. R: A Language and Environment for Statistical Computing [Internet]. Vienna: R Foundation for Statistical Computing; 2016. Available: http://www.R-project.org/

2734. HS Global Inc. Eviews [Internet]. CA, USA: HS Global Inc.; 2016. Available: http://www.eviews.com

2835. TIBCO Software Inc. Statistica [Internet]. CA, USA: TIBCO Software Inc.; Available: https://docs.tibco.com/products/tibco-statistica-13-3-0

2936. Hallmann CA, Sorg M, Jongejans E, Siepel H, Hofland N, Schwan H, et al. More than 75 percent decline over 27 years in total flying insect biomass in protected areas. PloS One. 2017;12: e0185809.

37. Moore CG. Interdisciplinary research in the ecology of vector-borne diseases: Opportunities and needs. Journal of Vector Ecology 2008;33(2):218–224.

38. Zimmerman B. Engaging with Complexity: Thrive! A Plan for a Healthier Nova Scotia. 2014; [e-print] Available from: https://thrive.novascotia.ca/sites/default/files/Thrive-Summit-2014-Brenda-Zimmerman-En.pdf.

39. Lupulović D, Martin-Acebes MA, Lazić S, Alonso-Padilla J, Blazquez AB, Escribano-Romero E, et al. First serological evidence of West Nile virus activity in horses in Serbia. Vector Borne Zoonotic Dis. 2011;11(9):1303–5.40.

40. Petrović T, Blazquez AB, Lupulović D, Lazić G, Escribano-Romero E, Fabijan D, et al. Monitoring West Nile virus (WNV) infection in wild birds in Serbia during 2012: First isolation and characterisation of WNV strains from Serbia. Eurosurveillance. 2013;18(44):1–8.

41. Petrović V, Turkulov V, Ilić S, Milošević V, Petrović M, Petrić D, et al. First report of imported case of dengue fever in Republic of Serbia. Vol. 14, Travel Medicine and Infectious Disease. 2016. p. 60–1.

42. van der Linden P and Mitchell JFB, editors. ENSEMBLES Climate Change and its Impacts. Summary of research and results from the ENSEMBLES project. Exeter: Met Office Hadley Centre;. 2009.:

43. Ministry of Environmental Protection of the Republic of Serbia. Second National Communication of the Republic of Serbia under the United Nations Framework Convention on Climate Change [Internet]. Belgrade: The Ministry; 2017 Aug. 162p [cited 2019 Sep 10]. Available from: http://www.klimatskepromene.rs/wp-content/uploads/2017/09/SNC_eng.pdf30

44. Adamović Ž. Distribution and abundance of anopheline mosquitoes (Diptera, Culicidae) in Vojvodina, Serbia. Acta Entomol Jugosl. 1979;15: 82.

~~31~~45. Halgoš J, Benková I. First record of *Anopheles hyrcanus* (Diptera: Culicidae) from Slovakia. Biológia. 2004;59: 68.

~~32~~46. Votýpka J, Šeblová V, Rádrová J. Spread of the West Nile virus vector *Culex modestus* and the potential malaria vector *Anopheles hyrcanus* in central Europe. J Vector Ecol. 2008;33: 269–277. doi:10.3376/1081-1710-33.2.269

~~33~~47. Lebl K, Brugger K, Rubel F. Predicting *Culex pipiens*/*restuans* population dynamics by interval lagged weather data. Parasit Vectors. 2013;6: 129. doi:10.1186/1756-3305-6-129

~~34~~48. Novikov YM, Vaulin OV. Expansion of *Anopheles maculipennis* s.s. (Diptera: Culicidae) to northeastern Europe and northwestern Asia: Causes and Consequences. Parasit Vectors. 2014;7: 389. doi:10.1186/1756-3305-7-389

~~35~~49. Seidel B, Nowotny N, Duh D, Indra A, Hufnagl P, Allerberger F. First records of the thermophilic mosquito *Culiseta longiareolata* (Macquart, 1838) in Austria, 2012, and in Slovenia, 2013. J Eur Mosq Control Assoc. 2013;31: 17–20.

~~36~~50. Zittra C, Waringer J, Werblow A, Melaun C, Fuehrer H-P. Reconfirmation of *Culiseta* (*Allotheobaldia*) *longiareolata* (Macquart 1838) (Diptera: Culicidae) in Austria. The first sequence-confirmed findings in northeastern Austria. Acta ZooBot Austria. 2014;150: 17–24.

~~37~~51. Tippelt L, Walther D, Kampen H. The thermophilic mosquito species *Uranotaenia unguiculata* Edwards, 1913 (Diptera: Culicidae) moves north in Germany. Parasitol Res. 2017;116: 3437–3440. doi:10.1007/s00436-017-5652-2

~~38~~52. Riordan DF. Effects of ultraviolet radiation on adults of *Aedes aegypti* (L.) (Diptera : Culicidae). Mosq News. 1969;29. Available: https://www.cabdirect.org/cabdirect/abstract/19711000053

~~39~~53. Hori M, Shibuya K, Sato M, Saito Y. Lethal effects of short-wavelength visible light on insects. Sci Rep. 2014;4: 7383. doi:10.1038/srep07383

~~40~~54. Kramer L, Li J, Shi PY. West Nile virus. Lancet Neurol. 2007;6:171–181.

55. Blitvich BJ. Transmission dynamics and changing epidemiology of West Nile virus. Anim Health Res Rev. 2008;9:71–86.

56. Komar N. West Nile virus: epidemiology and ecology in North America. Adv Vir Res. 2003;6:185–234.

57. Calistri P, Giovannini A, Hubalek Z, Ionescu A, Monaco F, Savini G et al. Epidemiology of West Nile in Europe and in the Mediterranean basin. Open Virol J. 2010;4(1):29–37.

58. Medić S, Lazić S, Petrović T, Petrić D, Samojlović M, Lazić G et al. Evidence of the first clinical case of equine neuroinvasive West Nile Disease in Serbia, 2018. Acta veterinaria 2019;69(1):123–130.

59. Paz S, Malkinson D, Green MS, Tsioni G, Papa A, Danis K, et al. Permissive Summer

Temperatures of the 2010 European West Nile Fever Upsurge. PLOS ONE. 2013;8: e56398. doi:10.1371/journal.pone.0056398

~~41~~60. Marcantonio M, Rizzoli A, Metz M, Rosà R, Marini G, Chadwick E, et al. Identifying the Environmental Conditions Favouring West Nile Virus Outbreaks in Europe. Gourbiere S, editor. PLOS ONE. 2015;10: e0121158. doi:10.1371/journal.pone.0121158

~~42.~~ 61~~. Petrić D, Petrović T, Hrnjaković Cvjetković I, Zgomba M, Milošević V, Lazić G, et al. West Nile virus "circulation" in Vojvodina, Serbia: Mosquito, bird, horse and human surveillance. Mol Cell Probes. 2017;31: 28–36. doi:10.1016/j.mcp.2016.10.011~~

~~43~~. Tran A, Sudre B, Paz S, Rossi M, Desbrosse A, Chevalier V, et al. Environmental predictors of West Nile fever risk in Europe. Int J Health Geogr. 2014;13: 26. doi:10.1186/1476-072X-13-26

~~44~~62. Paz S. Climate change impacts on West Nile virus transmission in a global context. Philos Trans R Soc B Biol Sci. 2015;370: 20130561–20130561. doi:10.1098/rstb.2013.0561

~~45~~63. Narayanan DL, Saladi RN, Fox JL. Ultraviolet radiation and skin cancer. Int J Dermatol. 2010;49: 978–986. doi:10.1111/j.1365-4632.2010.04474.x

~~46~~64. Lacy K, Alwan W. Skin cancer. Medicine (Baltimore). 2013;41: 402–405. doi:10.1016/j.mpmed.2013.04.008

~~47~~65. Leun JC van der. Uv-Carcinogenesis. Photochem Photobiol. 1984;39: 861–868. doi:10.1111/j.1751-1097.1984.tb08872.x

~~48~~66. Malinovic-Milicevic S, Mihailovic DT, Radovanovic MM. Reconstruction of the erythemal UV radiation data in Novi Sad (Serbia) using the NEOPLANTA parametric model. Theor Appl Climatol. 2015;121: 131–138. doi:10.1007/s00704-014-1223-y

~~49~~67. Malinović-Milićević S and Radovanović M 2016 UV radiation and heat wives in Vojvodina (Book 87) (Belgrade: Serbian Academy of Sciences and Arts, Institute of Geography „Jovan Cvijić") p ISBN.

~~50~~68. de Vries E, Tyczynski J, Parkin MD. Cutaneous malignant melanoma in Europe. Eur Netw Cancer Regist Int Agency Res Cancer. 2003;ENCR Cancer Fact Sheets no.4 1-4.

~~51~~69. Demers AA, Nugent Z, Mihalcioiu C, Wiseman MC, Kliewer EV. Trends of nonmelanoma skin cancer from 1960 through 2000 in a Canadian population. J Am Acad Dermatol. 2005;53: 320–328.

70. Wu S, Han J, Laden F, Qureshi AA. Long-term ultraviolet flux, other potential risk factors, and skin cancer risk: a cohort study. Cancer epidemiology, biomarkers and prevention: a publication of the American Association for Cancer Research, cosponsored by the American Society of Preventive Oncology. 2014;*23*(6):1080–1089.

71. Vandenbroucke JP, Pearce N. Incidence rates in dynamic populations. Int J Epidemiol. 2012;41(5):1472–1479.

Supporting information

S1 Table. Overview of dry-ice trap samples sizes. For each year, the number of locations sampled, the number of location re-sampled, and total number of samples are presented. Exposure time at the trap locations was similar (14 ± 2h).

S2 Table. Number of the total trap nights, positive trap nights and *Anopheles hyrcanus* specimens sampled at 54 selected sites in the Vojvodina Province, Serbia during the years 1985-86, 2004-5 and 2014-15.

S3 Table. Frequency of sampling of WNV infected mosquitoes (1 – 5 times) in the Vojvodina Province, Serbia, during the period 2010-2016.

Journal: PLOS ONE

Collection: Urban Ecosystems

Manuscript#: PONE-D-19-16900

Title of paper: Assessment of climate change impact on the malaria vector *Anopheles hyrcanus*, West Nile disease, and incidence of melanoma in the Vojvodina Province (Serbia) using data from a regional climate model

Dragutin T. Mihailović[1], Dušan Petrić[2*], Tamaš Petrović[3], Ivana Hrnjaković-Cvjetković[4,5], Vladimir Djurdjevic[6], Emilija Nikolić-Đorić[7], Ilija Arsenić[1], Mina Petrić[8,9,10*], Gordan Mimić[11], Aleksandra Ignjatović-Ćupina[2]

Dear Dr Samy,

We are pleased to submit the revised version of "Assessment of climate change impact on the malaria vector *Anopheles hyrcanus*, West Nile disease, and incidence of melanoma in the Vojvodina Province (Serbia) using data from a regional climate model" (#PONE-D-19-16900). We appreciate the time and efforts by the editor and advisors in reviewing the manuscript. Please find below detailed responses to the reviewers, whom we thank for their careful consideration of the manuscript. We also reviewed the manuscript for any additional errors and made small changes that are tracked in the attached document ("Revised Manuscript with Track Changes").

Line numbers for the corrected text are given in red according to the enumeration in the file "Revised Manuscript with Track Changes".

Reviewer #1: Comments on the manuscript PONE-D-19-16900

Title: Assessment of climate change impact on malaria vectors, West Nile disease, and incidence of melanoma in the Vojvodina Province (Serbia) using data from a regional climate model. Authors: Dragutin T. Mihailović, Dušan Petrić, Tamaš Petrović, Ivana Hrnjaković Cvjetković, Vladimir Đurđević, Emilija Nikolić Đorić, Ilija Arsenić, Mina Petrić, Gordan Mimić

The work presented in paper Mihailović et al. is interesting. The objective of the authors was to compare data from the mosquito field collections and medical studies with regional 29 climate model projections to examine the impact of climate change on the circulation of West Nile virus (WNV), the spreading of the malaria vector Anopheles hyrcanus and the incidence of melanoma. The comparison was done with the coupled regional Eta Belgrade University and Princeton Ocean Model for the period 1961-2015 using the A1B scenario, and the expected changes up to 2030. Overall, significant correlation was found between the frequency of WNV in Culex pipiens and the overwintering temperature averages and seasonal relative humidity at the sampling sites. Correlation was also found between the spreading and relative abundance of Anopheles hyrcanus and the trend of the mean annual temperature. There was also an increase in melanoma incidence.

Minor comments to authors

Title

Authors wrote “malaria vectors” but the only presented data on only one vector Anopheles hyrcanus

Corrected according to the suggestion.

Abstract

L32: ……………and 10-years. Delete the hyphen

L36: *Corrected according to the suggestion.*

L37: ……………Culex. pipiens. Delete the dot

L44: *We abbreviated the genus name to Cx. because name was spelled in the previous sentence.*

Introduction

L49-53: Are the authors referring to themselves when they stated, “The authors (……….), have been working together…….”?

Yes, we tried to make it clear with correction below.

L56: **Corrected to:** “The authors of the manuscript (……….), have been working together…….”

L72: Authors should write, “Climate change……..” instead of “The climate change……..”

L86: *Corrected according to the suggestion.*

L75: Author should write, "Melanoma mortality…………..within the period 1985-2004

L89-91: *Corrected according to the suggestion.*

L78: Authors should define the acronym ENCR, as this is used here for the first time.

L83: … using ENCR data

L93-94: Changed to:

… using European Network of Cancer Registries (ENCR) data

L81-82: Authors should use past tense in the sentence "……we compared considerable of previously……"

L97: *Corrected according to the suggestion.*

L54-55: This sentence is not clear for me. I suggest this: "In this paper, we analysed observed data collected over a period of 31 years……"

L61: *Corrected according to the suggestion, "collected" erased because of tautology.*

Materials and Methods
L96: Authors should define SRES-A1B scenario for the first time.

The SRES-A1B scenario is defined in the text, and central differences to RCP explained. Due to this, authors think that selection of scenario, to some extent, is irrelevant for the presented results.

L96**:** … and the period 2001-2030 using the SRES-A1B scenario.

L113-122 Corrected to:

… and the period 2001-2030 according to the A1B scenario defined in Special Report on Emissions Scenarios (SRES) (Nakićenović and Swart, 2000), from now on SRES-A1B. SRES scenarios, which defined future global greenhouse gases emissions, were extensively used in the Intergovernmental Panel on Climate Change (IPCC) Third, and Fourth Assessment Reports. The main storyline behind the A1B scenario is rapid economic growth, followed by a significant increase in greenhouse gases concentrations in the future. In the Fifth Assessment Report (AR5), the Representative Concentration Pathway (RCP) is introduced, which are possible future

concentration pathways without any storyline behind them. Comparing SRES-A1B and RCPs in terms of the greenhouse gases concentrations, at the end of this century SRES-A1B is the closest to RCP6.0, but for the time horizon used in this study, up to 2030, the difference between any SRES or RCPs are relatively small.

New references included:

Summary for Policy makers. In: Nakićenović N, Swart R, editors. Special Report on Emissions Scenarios. Cambridge: Cambridge University Press - Published for the Intergovernmental Panel on Climate Change. 2000. pp. 1-21

L115: Authors used only one formula, the subtitle should be in singular

L141: *Corrected according to the suggestion.*

Results

- Authors should specify the exact p-values instead of writing $p<0.05$ or $p>0.05$

L260-281: *Corrected according to the suggestion. The section was updated to include the exact p-values.*

L207: "The Poisson regression model for the dependence of a number of detections per site (frequency-λ)…………………………is highly significant". Authors stated it was highly significant, but from my perspective, $p<0.05$ is not a specific indication of high significance. Could the give the exact value of p?

L282-283: *Corrected according to the suggestion. Exact p value is not below 0,01 (it is 0.01393) which is considered as high significance by many authors, so we erased the word "highly".*

- Fig2b and 2c are fuzzy

Thank you for your comment. The figures were reformatted to higher resolution according to PACE.

- Fig4. Colors of fig4c are confusing

Figures 4c has been adapted to have more contrasting colors.

It will be more interesting if the authors used only vector-borne diseases data in this paper.

N.B: Other comments are incorporated in the manuscript

Authors appreciate very much the effort invested in the improvement of the manuscript quality. All suggestions are incorporated into the revised version except one concerning the spelling of NUTS. Nomenclature of territorial units for statistics is originally abbreviated NUTS from the French version (Nomenclature des Unités territoriales statistiques).

Reviewer #2:
Authors are presenting an interesting paper regarding the effects of climate change in Northern Serbia considering three independent measures: The spread of Anopheles hyrcanus, the presence of West Nile Virus in Culex pipiens, and the incidence of melanoma cases. The paper is interesting, however, discussion should be improved specially on the uncertainty of future predictions since they are using just one climatic model. Further, their results should be stated more carefully since their model rely on assumptions (e.g., manually selected variables) which are also not clearly stated.

Discussion is corrected according to the reviewer's suggestion:

L345-352: New text added:

The temperature trend over the period of observations used in this study and for the future time horizon following A1B scenario obtained with the EBU-POM regional climate model is within the multi-model ensemble (MME) spread of regional climate models with similar configuration used in the ENSEMBLES project (van der Linden and Mitchell, 2009). For the period 2001-2030 the temperature change for the region of interest in the EBU-POM integration is 0.75 °C concerning the period 1961-1990 and for the same period ENSEMBLES MME spread range is 0.5-1.5 °C (MEP, 2017). Following this finding, other results presented in this paper that relay on temperature change, can be seen as an estimate that will be within uncertainty related to the future temperature projection.

New references included:

van der Linden P and Mitchell JFB, editors. ENSEMBLES Climate Change and its Impacts. Summary of research and results from the ENSEMBLES project. Exeter: Met Office Hadley Centre;. 2009.:

Ministry of Environmental Protection of the Republic of Serbia. Second National Communication of the Republic of Serbia under the United Nations Framework Convention on Climate Change [Internet]. Belgrade: The Ministry; 2017 Aug. 162p [cited 2019 Sep 10]. Available from: http://www.klimatskepromene.rs/wp-content/uploads/2017/09/SNC_eng.pdf

Major comments:

The paper is showing results in the order: malaria vector, WNV, and melanoma. I suggest following the same order in the abstract.

Changed as suggested.

Authors are using one of the SRES future climatic scenarios; currently the standard for future climate studies are the RCP scenarios. Authors should describe the nature of the SRES-A1B scenario, which is not mentioned in any part of the study. Further, authors should explicitly discuss uncertainty on their predictions since they are not using other scenarios or other climatic models.

Authors addressed this comment in the text corrected. Please check response to the L113-122 and L 345-352 above.

Lines 176-180. There is no discussion or results regarding these sentences. Was the comparison between EBU-POM model and the Republic Hydrometeorological Service of Serbia perfect? What is the implication of this approach on the overall paper?

This is a valuable comment since the information measure(s) is(are) a good indicator of the reliability of model outputs and thus on the overall paper. The increasing complexity of climate models is a growing concern in the modelling community. However, because we invested a serious effort to make our models more "realistic", we included more parameters and processes. With increasing model complexity, we are less able to manage and understand the model behaviour. Thus, from a user's perspective, the following question is entirely natural: "How complex model (EBU-POM model in our case) do I need to use to study this problem (assessment of climate change impact on malaria vectors, West Nile disease, and incidence of melanoma in the VPS) with this data set (temperature and/or precipitation)?". In the revised version, we inserted the additional text.

L229-249: New text added:

We considered the papers by Mihailović et al. [2,24] in which Kolmogorov complexity measures (Kolmogorov complexity (KC), Kolmogorov complexity spectrum KC spectrum) and the highest value of the KC spectrum (KCM)) and sample entropy (SE) [25] were used to quantify the regularity and complexity of air temperature and precipitation time series, obtained by the EBU-POM model, representing both deterministic chaos and stochastic processes. We considered the complexity of the EBU-POM model using the observed and modelled time series of temperature and precipitation. We computed the KC spectrum, KC, KCM and SE values for temperature and precipitation. The calculations were performed for the entire time interval

1961–1990: (1) on a daily basis with a size of N =10958 samples for temperature and (2) on a monthly basis with a size N =360 for the precipitation. The simulated time series of temperature and precipitation were obtained by the EBU-POM model for the given period. The observed time series of temperature and precipitations for two stations: Sombor (SO) (88 m a.s.l.) and Novi Sad (NS) (84 m a.s.l.) in the considered area, were taken from daily meteorological reports of the Republic Hydrometeorological Service of Serbia. For both sites, the modelled complexity is lower than the observed one, but with the reliability which is in the interval values allowed by the information measures (KC, KCM, and SE) (Krzic et al. 2011, Dell' Aquila et al. 2016, Cavicchia et al. 2016). These findings mean that the models with a KC (and KCM) complexity lower than the measured time series complexity cannot always reconstruct some of the structures contained in the observed data. However, it does not mean that outputs from EBU-POM model do not correctly simulate climate elements since both sites values indicate the absence of stochastic influences, providing reliable projections of the climate elements.

New references included:

Krzic A, Tosic I, Djurdjevic V, Veljovic K, Rajkovic B. Changes in some indices over Serbia according to the SRES A1B and A2 scenarios. Climate Research. 2011;49: 73-86.

Cavicchia L, Scoccimarro E, Gualdi S, Marson P, Ahrens B, Berthou S, et al. Mediterranean extreme precipitation: a multi-model assessment. Clim. Dyn. 2016. doi: 10.1007/s00382-016-3245-x

Dell' Aquila A, Mariotti A, Bastin S, Calmanti S, Cavicchia L, Deque M, et al. Evaluation of simulated decadal variations over the Euro-Mediterranean region from ENSEMBLES to Med-CORDEX. Clim. Dyn. 2016. doi:10.1007/s00382-016-3143-2

Line 277-280: There is no evidence in this paper supporting this affirmation since the variables analyzed corresponded to three temperature related variables and just one considering humidity. Moreover, results were never compared statistically; modify accordingly.

Corrections made as suggested. The sentence "It seems that temperature in semi-urban areas dominates the other environmental factors influencing WNV circulation in nature (e.g. landscape suitability for reservoir host and mosquito vector, host availability, precipitation), as it is the primary factor affecting both mosquito vector abundance and virus replication." *now reads as:*

L400-404: Corrected to: It seems that temperature in semi-urban areas is an essential environmental factor influencing WNV circulation (landscape suitability for reservoir host and mosquito vector, host availability, and precipitation/water availability are somewhat similar in

investigated semi-urban areas of VPS), as it affects both mosquito vector abundance and virus replication.

Figure 2: Expand the acronym CRCM. Also, double check the legend, which is describing red and green colors but the figure is only showing different shades of blue.

Corrections made as suggested.

L173-179: Fig 2. (a) The regional climate model EBU-POM projection of the mean annual air temperature (T_a) for the period 1985 - 2030 and: i) number of specimens sampled in one trap during single sampling period (blue columns); ii) the number of sites invaded by *An. hyrcanus* (light blue columns); and iii) relative number of positive samplings per year (dark blue columns), (b) projected increase in the number of sites invaded by *An. hyrcanus* (the period 2001-2030 ±S.E.), and (c) projected increase in the number of the specimens sampled in one trap during single sampling period (2001 - 2030 ±S.E.).

Figure 4: Add WD and HD to the corresponding legend of the graphic. Is there a Croatian sentence in the legend? Please describe how the melanoma incidence was calculated, is the y axis showing incidence or number of cases? Cumulative incidence is known to over-represent trends (see reference: Vandenbroucke & Pearce, 2012, doi: 10.1093/ije/dys142), try to use incidence rate instead.

This is a keystone issue in this field of epidemiology. However, it is still under a broad umbrella of discussion. In particularly mentioned reference (Vandenbroucke & Pearce, 2012) the Authors comprehensively considered the place of incidence rates in dynamic populations as well as the cumulative incidence (risk or portion) from an epistemological point of view and also giving very illustrative (educational) examples. Many authors were arguing with some ideas explicated in this paper, also considering some examples. We agree with V&P ideas, but we did not find the place where they explicitly say that it would always be using the incidence rate instead of cumulative (the question of overestimation). To our understanding, they left the space for a situation when the use of cumulative incidence gives acceptable results. For example, it is partly seen in the paper by Wu et al. (2014). There is another moment why we used cumulative incidence. It is well-known that there is a high correlation between sun exposure (and received cumulated doses of the UV radiation) and melanoma. If that doses (or any climate element) on a daily basis are used from regional climate models, they cannot be directly correlated with daily or monthly measured or calculated biological quantities. The reason for that is the fact that from regional climate models, we can estimate just the trend of the considered physical quantity

(in our case -UV doses through their cumulative values). Correspondingly it is correlated with cumulative incidence. Having said that, after the end of the statement in Line 336, we inserted the following text.

The legend in Fig. 4(c) and y-axis in Fig 4 (d) are changed as suggested.

The M&M - Melanoma incidence and UVR was amended by the following text:

L216-221: New text added:

In the analysis we have used two indicators: (i) melanoma incidence rate that is a measure of the number of new cases ("incidence") per unit of time ("rate") and (ii) cumulative incidence ("incidence proportion" that measures the number of new cases per person in the population over a defined period of time – often called risk or proportion). Melanoma incidence rate (per 100,000 people) for ten years 1995 - 2004 was based on the data obtained from the paper by Jovanović et al. [7]. From these data, we calculated the cumulative incidence.

The discussion was also amended by the following text:

L437-441: New text added:

In a cohort study, Wu et al. (2014) considered the impact of long-term UV radiation flux on skin cancer risk. Comparing with participants in the lowest quintile of cumulative UV radiation flux in adulthood, they found that participants in the highest quintile had multivariable-adjusted risks (cumulative incidence). According to Vandenbroucke and Pearce (2012), some studies where cumulative incidence is used can over-represent the trends.

New references included:

Wu S, Han J, Laden F, Qureshi AA. Long-term ultraviolet flux, other potential risk factors, and skin cancer risk: a cohort study. Cancer epidemiology, biomarkers and prevention: a publication of the American Association for Cancer Research, cosponsored by the American Society of Preventive Oncology. 2014;*23*(6):1080–1089.

Vandenbroucke JP, Pearce N. Incidence rates in dynamic populations. Int J Epidemiol. 2012;41(5):1472–1479.

In table S3 consider adding the number of mosquito samples per site.

Number of samples added in the table.

Figure 6 can be replaced with the statistics of such graphic for readers' interpretation.

L442-446: *Corrections made as suggested. Figure 6 deleted, the paragraph now reads as:*

From a statistical point of view, the linear regression model for modelling the cumulative incidence of melanoma versus the difference of the cumulative UVR doses for hot and warm days (Fig 4d) is acceptable. Parameters are statistically highly significant (r = 0.9712 and p = 0.000003) while analysis of residual distribution shows a good agreement with the normal distribution (Shapiro-Wilk test, W = 0.9608, p = 0.7952).

Authors are justifying the paper under the 'One Health' concept, however they are not discussing the idea further. I would like discussing explicitly the benefits of putting together a set of multidisciplinary specialists to the development of the manuscript and how this contribution is part of the one health concept.

The discussion was amended by the following text:

L322-344: New text added:

Despite globalisation trends, researchers have become "closed" in their ever-smaller communication circles which are not limited by state or national borders but by the professional language and way of thinking. Thus, by the end of the 20th century, the scientific community has been faced with problems in communication within its confines. One of the principal reasons why vector-borne diseases (VBD) are so difficult to predict, is the complex interaction of multiple factors (vector, host, pathogen, environment including short-term weather patterns and long-term climate change) in space and time (Moore 2008, Zimmerman 2014). Only groups from multiple sectors that communicate and work together on specific aspects of VBD systems will be able to answer the most exciting and pressing problems in the field (Moore 2008). Authors of this paper started collaboration in 2003 comparing the climates of the foci of WNV circulation in USA (California Central Valley) and Europe (Bucharest area) with VPS. As compared climates showed quite similar patterns, colleagues from public health and veterinary joined the initial group of meteorologists and medical entomologists. With the idea to better draw upon the resources and insights of the various sectors we designed and implemented research and programmes to achieve better outcomes in the control of zoonoses (diseases that can spread between animals and humans, e.g., WNV disease). This led us to the following achievements: (i) the first detection of WNV in horses in Serbia in 2009 (Lupulović 2011); (ii) the first detection of WNV in mosquitoes in Serbia in 2010 (26); (iii) the first detection of WNV in wild birds in Serbia in 2012 (Petrović 2013); (iv) development and

implementation of the national programme of WNV surveillance in mosquito, bird and horse population [8], combined with human surveillance in VPS from 2014; (v) increased visibility to ECDC, EFSA and WHO; (vi) the first detection of imported dengue human case in Serbia in 2016 (Petrović 2016); and (vii) development and implementation of "One Health" programme in VPS from 2018. We are quite sure that much less would have been achieved without multidisciplinary communication and collaboration initiated in 2003, and this paper would not have been compiled.

New references included:

Moore CG. Interdisciplinary research in the ecology of vector-borne diseases: Opportunities and needs. Journal of Vector Ecology 2008;33(2):218–224.

Zimmerman B. Engaging with Complexity: Thrive! A Plan for a Healthier Nova Scotia. 2014; [e-print] Available from: https://thrive.novascotia.ca/sites/default/files/Thrive-Summit-2014-Brenda-Zimmerman-En.pdf.

Petrić D, Hrnjaković-Cvjetković I, Radovanov J, Cvjetković D, Jerant-Patić V, Milošević V, et al. West Nile virus surveillance in humans and mosquitoes and detection of cell fusing agent virus in Vojvodina Province (Serbia). HealthMED 2012;6(2):462–68.

Lupulović D, Martin-Acebes MA, Lazić S, Alonso-Padilla J, Blazquez AB, Escribano-Romero E, et al. First serological evidence of West Nile virus activity in horses in Serbia. Vector Borne Zoonotic Dis. 2011;11(9):1303–5.

Petrović T, Blazquez AB, Lupulović D, Lazić G, Escribano-Romero E, Fabijan D, et al. Monitoring West Nile virus (WNV) infection in wild birds in Serbia during 2012: First isolation and characterisation of WNV strains from Serbia. Eurosurveillance. 2013;18(44):1–8.

Petrović V, Turkulov V, Ilić S, Milošević V, Petrović M, Petrić D, et al. First report of imported case of dengue fever in Republic of Serbia. Vol. 14, Travel Medicine and Infectious Disease. 2016. p. 60–1

Minor comments:

Please use Oxford comma across the manuscript: e.g., Line 30: 'the malaria vector, and the incidence of melanoma'.

Corrections made as suggested.

Line 28: Authors never discuss problems related with animal health, thus, I suggest avoiding this kind of affirmations (e.g., line 81).

The reviewer is right, we did not, but we think it is vital to mention animals because WNV is the important zoonotic diseases. Therefore, we would like to include new paragraphs in Introduction and Discussion.

The introduction was amended by the following text:

L76-80: New text added:

In Europe, the total number of reported autochthonous WNV infections in 2018 (n=2,083) exceeded, by far, the total number from the previous seven years (n=1,832). During the same transmission season, outbreaks of West Nile fever among equids increased by 30% compared to the number of outbreaks in 2017. In total, 285 outbreaks among equids were reported by the EU Member States in 2018.

New references included:

Epidemiological update: West Nile virus transmission season in Europe, 2018 [Internet]. [cited 2019 Sep 09]. Available from: https://ecdc.europa.eu/en/news-events/epidemiological-update-west-nile-virus-transmission-season-europe-2018

Also, the discussion was amended by following text:

L377-390: New text added:

The WNV transmission cycle involves mosquito vectors and birds, but equines and humans are also susceptible to infection (Kramer et al. 2007, Blitvich 2008). Although WNV infections have been described in a wide variety of vertebrates, birds are the main natural reservoir. Hundreds of wild and domestic avian species have been described as susceptible to WNV infection, but many of these showed only subclinical infection (Komar, 2003). In horses, WNV infection is also frequently clinically unapparent, but around 10% of cases develop neurological disorders with up to 50% mortality rates (Blitvich 2008, Calistri et al. 2010). An increasing number of severe outbreaks in horses have been reported in Europe in the past decade, including a large one that took place in northeast Italy in 2008 involving 251 stables with 794 cases and five deaths (Calistri et al. 2010). From the first detection of WNV in 8 out of 81 found dead wild birds in Serbia (Petrovic et al. 2013), each year WNV nucleic acid was detected in found dead or captured wild birds during summertime (Petrovic et al. 2018). Serological testing of horses sampled during 2009-2010 showed that 12% of 349 horses from the northern part of the Serbia had neutralizing WNV antibodies (Lupulovic et al. 2012). After that, each year horse WNV cases were detected by the positive serological response (IgG and IgM antibody seroconverted horses) (Petrovic et al., 2018) or as a clinical manifestation of

West Nile neuroinvasive disease (Medić et al., 2019).

New references included:

Kramer L, Li J, Shi PY. West Nile virus. Lancet Neurol. 2007;6:171–181.

Blitvich BJ. Transmission dynamics and changing epidemiology of West Nile virus. Anim Health Res Rev. 2008;9:71–86.

Komar N. West Nile virus: epidemiology and ecology in North America. Adv Vir Res. 2003;6:185–234.

Calistri P, Giovannini A, Hubalek Z, Ionescu A, Monaco F, Savini G et al. Epidemiology of West Nile in Europe and in the Mediterranean basin. Open Virol J. 2010;4(1):29–37.

Medić S, Lazić S, Petrović T, Petrić D, Samojlović M, Lazić G et al. Evidence of the first clinical case of equine neuroinvasive West Nile Disease in Serbia, 2018. Acta veterinaria 2019;69(1):123–130.

Line 28: Methods on the paper should be written in past tense: e.g., COMPARED. Review this in the rest of the manuscript, e.g., line 82.

Corrections made as suggested.

Line 30: 'the spreading of ONE malaria vector'

L33-34: *Corrections made as suggested.*

Line 37: 'Culex.' should be corrected, only Cx.?

L44: *Corrections made as suggested.*

Line 40: This is the first time you are mentioning HD, please expand the acronym, review this in the rest of the manuscript, for example EU in line 50, or ENCR in line 78.

Changed as suggested.

L40: of days with Tmax ≥ 30 °C (HD)

L47: Changed to: of days with Tmax ≥ 30 °C (Hot Days - HD)

L50: … endorsed by the EU

L58-59: Changed to: … endorsed by the European Union (EU)

L79: … using ENCR data

L93-94: Changed to: … using European Network of Cancer Registries (ENCR) data

Line 44: Specify the risk that you are addressing with this research.

Changed as suggested.

L51: New text added: … of vector-borne diseases and melanoma.

Line 54: extra 'Collected' after 'observed data', please erase.

Changed as suggested, the beginning of the sentence now reads as:

L61: In this paper, the authors collected and analysed observed data over a period

Line 55: Add 'are' after the word 'melanoma' at the end of the sentence.

L63: *Changed as suggested.*

Line 58: Here you need a reference for the environmental threat represented for the animal and humans at the Pannonian plane.

L65: *References included as suggested.*

Line 63: You need a reference for the affirmation of malaria as worldwide detrimental vector-borne disease.

L72: *New reference added as suggested.*

World Health Organization, World Malaria Report 2018. Geneva: The Organization; 2018.

Line 70: Consider adding a reference of how temperature and relative humidity are principal abiotic factors for WNV and An. hyrcanus.

Not sure how to respond to this comment. However, references concerning the vector-borne disease and mosquito vector mentioned in the sentence are already given in the text - [10,11].

Line 76: Be consistent across the whole manuscript, use either - or – without spaces to separate year timeframes, 1976–2004 is preferred.

Changed as suggested.

Line 102: Add corresponding reference for the Köpen classification.

L128: *New reference added as suggested.*

Kottek M, Grieser J, Beck C, Rudolf B, Rubel F. World Map of the Köppen-Geiger climate classification updated. Meteorologische Zeitschrift. 2006;15(3):259–263.

Line 122: "Data were..."

L148: *Changed as suggested.*

Line 126: 1985–1986.

L152-153: *Changed as suggested.*

Line 158: Briefly describe the method of WNV detection, i.e., RT-PCR or the corresponding one before referencing Petrovic et al 2018.

L193-199: New text added:

Mosquito pools were tested for WNV RNA presence by TaqMan-based one-step reverse transcription real-time PCR (RT-qPCR) that amplified both lineage 1 and 2 strains. Viral RNA was extracted using the commercial *ISOLATE II RNA Mini Kit* (Bioline, The Netherlands) according to the manufacturer's instruction. One-step RT-qPCR was conducted using the commercial kit *RNA UltraSense™ One-Step qRT-PCR System* (Life Technologies Corporation) with the primers and probe that targeted the nucleocapsid protein C gene regions of WNV, as described by Petrović et al. (2018).

Line 172: Describe the indicators briefly before referencing Jovanovic et al 2009.

According to the suggestion, the text placed between 172-174 lines is replaced by the following one.

L216-221: New text added:

In the analysis we have used two indicators: (i) melanoma incidence rate that is a measure of the number of new cases ("incidence") per unit of time ("rate") and (ii) cumulative incidence ("incidence proportion" that measures the number of new cases per person in the population over a defined period of time – often called risk or proportion). Melanoma incidence rate (per 100,000 people) for ten years 1995 - 2004 was based on the data obtained from the paper by Jovanović et al. [14]. From these data, we calculated the cumulative incidence.

Line 227: Is the formula correct: warm days - WD?

L301-302: Changed to:

air temperature $T_{max} \geq 25$ °C (Warm Days - WD)

Line 263: Consider changing 'indicate that the findings supporting' by 'support'

L370-374: *The sentence was quietly confusing; we rewrote it to read like this:*

Positive trends which are present in our observations might indicate that the findings on the negative influence of UVR and blue-light radiation (this radiation has a wavelength between approximately 380 nm and 500 nm; it has a very short wavelength, and so produces a higher amount of energy) on adult mosquitoes under laboratory conditions [38,39] could not be simply translated to the field.

Line 273: Authors are not showing incidence rates, just presence of WNV in mosquitoes.

L396: *Changed as suggested. End of sentence now reads as:*

… with a higher frequency of WNV presence in mosquitoes.
