# Supplementary material for: Assessment of climate change impact on the malaria vector Anopheles hyrcanus, West Nile disease, and incidence of melanoma in the Vojvodina Province (Serbia) using data from a regional climate model
Source: PLoS One. 2020 Jan 15;15(1):e0227679. doi: 10.1371/journal.pone.0227679 (PMC6961917; doi:10.1371/journal.pone.0227679)

Journal: PLOS ONE

Manuscript#:PONE-D-19-16900R2

Title of paper:Assessment of climate change impact on the malaria vector*Anopheles hyrcanus*, West Nile disease, andincidence of melanoma in the Vojvodina Province (Serbia) using data from a regional climate model

Dragutin T. Mihailović[1], Dušan Petrić[2*], Tamaš Petrović[3], Ivana Hrnjaković-Cvjetković[4,5], Vladimir Djurdjevic[6], Emilija Nikolić-Đorić[7], Ilija Arsenić[1], Mina Petrić[8,9,10*], Gordan Mimić[11], Aleksandra Ignjatović-Ćupina[2]

Dear Dr Samy,

We are pleased to submit the revised version of “Assessment of climate change impact on the malaria vector *Anopheles hyrcanus*, West Nile disease, and incidence of melanoma in the Vojvodina Province (Serbia) using data from a regional climate model” (#PONE-D-19-16900R2). We appreciate the time and efforts by the editor and advisors in reviewing the manuscript. Please find below detailed responses to the reviewers, whom we thank for their careful consideration of the manuscript. We also reviewed the manuscript for any additional errors and made small changes that are tracked in the attached document (“Revised Manuscript with Track Changes”).

Reviewers' comments:

Reviewer #1: Apart from the last single remark made, that the authors should consider, all my comments have been addressed. From my perspective, this paper is acceptable for publication.

Corrected, Line 361in the file “Manuscript_R2”: “… expectedthe intensity ...”replaced by “… expected, the intensity ...”

Reviewer #2: Authors present a corrected version of their manuscript. In reference to my suggestions, the majority of them have been addressed appropriately. Some comments among their current version:

The authors have included a measure of uncertainty based on the results of the ENSEMBLES project, that is, based on an extrinsic source of climatic model uncertainty. This is potentially valid, but please be explicit on your approach, that is, discuss the absence of experimentation

with other climatic scenarios (e.g., SRES A2, B1, etc) and the decision to not use other climatic models apart from EBU-POM.

In the amended paragraph from lines 345-352, it is not clear if the EBU-POM model integration to 0.75ºC refers to the temperatures for 2001-2030 or 1961-1990, please improve the wording and punctuation.

Response:

Current version: For the period 2001-2030 the temperature change for the region of interest in the EBU-POM integration is 0.75 °C concerning the period 1961-1990 and for the same period ENSEMBLES MME spread range is 0.5-1.5 °C [43]. Following this finding, other results presented in this paper that relay on temperature change, can be seen as an estimate that will be within uncertainty related to the future temperature projection.

New version: *For the region of interest, temperature change for the period 2001-2030 with respect to the period 1961-1990, in the EBU-POM integration is 0.75 ℃ and in the case of ENSEMBLES MME spread range is 0.5-1.5 ℃ [43]. Furthermore, over period 2001-2030, uncertainty in temperature projections, related to the scenarios is just small fraction of total uncertainty (Hawkins and Sutton, 2009), due to the fact that there is no significant difference in greenhouse gases concentrations for different SRES scenarios. Consequently, it can be considered that for the period 2001-2030, in case of other scenarios, the estimated range will be similar. Following this findings, even that selection of single model and single scenario does not allow estimate of the full uncertainty in the future, presented results that relay on temperature change, can be seen as plausible future realisation within total uncertainty range, and realistic response of the complex system for further increase in temperature as a forcing driver.*

Hawkins E, Sutton R. The Potential to Narrow Uncertainty in Regional Climate Predictions. Bull Amer Meteor. 2009; 90(8): 1095–1108. https://doi.org/10.1175/2009bams2607.1

Also, we included the following sentence at the end of the discussion:

In the future studies, it will be beneficial to introduce results of other regional climate models, such as multi-model ensemble from the CORDEX framework, as a need for better understanding of different uncertainties in the results.

In the amended paragraph from lines 229-249, please explain clearly the sentence 'but with the reliability which is in the interval values allowed by the information measures'. Consider either quantitatively adding the values you are referring to, or define reliability and how a simple simulated model is preferred over complex models in order to explain the observed phenomena.

Response:

Current version: We considered the papers by Mihailović et al. [2, 28] in which Kolmogorov complexity measures (Kolmogorov complexity (KC), Kolmogorov complexity spectrum KC spectrum) and the highest value of the KC spectrum (KCM)) and sample entropy (SE) [29] were used to quantify the regularity and complexity of air temperature and precipitation time series, obtained by the EBU-POM model, representing both deterministic chaos and stochastic processes. We considered the complexity of the EBU-POM model using the observed and modelled time series of temperature and precipitation. We computed the KC spectrum, KC, KCM and SE values for temperature and precipitation. The calculations were performed for the entire time interval 1961–1990: (i) on a daily basis with a size of N =10958 samples for temperature and (ii) on a monthly basis with a size N =360 for the precipitation. The simulated time series of temperature and precipitation were obtained by the EBU-POM model for the given period. The observed time series of temperature and precipitations for two stations: Sombor (SO) (88 m.a.s.l.) and Novi Sad (NS) (84 m.a.s.l.) in the considered area, were taken from daily meteorological reports of the Republic Hydrometeorological Service of Serbia. For both sites, the modelled complexity is lower than the observed one, but with the reliability which is in the interval values allowed by the information measures (KC, KCM, and SE) [30, 31,32]. These findings mean that the models with a KC (and KCM) complexity lower than the measured time series complexity cannot always reconstruct some of the structures contained in the observed data. However, it does not mean that outputs from EBU-POM model do not correctly simulate climate elements since both sites values indicate the absence of stochastic influences, providing reliable projections of the climate elements.

New version: We considered the papers by Mihailović et al. [2, 28] in which Kolmogorov complexity measures *[Kolmogorov complexity (KC), Kolmogorov complexity spectrum (KC spectrum), and the highest value of the KC spectrum (KCM)]*, and sample entropy (SE) [29] were used to quantify the regularity and complexity of air temperature and precipitation time series, obtained by the EBU-POM model, representing both deterministic chaos and stochastic processes. We considered the complexity of the EBU-POM model using the observed and modelled time series of temperature and precipitation. We computed the KC spectrum, KC, KCM and SE values for temperature and precipitation. The calculations were performed for the entire time interval 1961–1990: (i) on a daily basis with a size of N =10958 samples for temperature and (ii) on a monthly basis with a size N =360 for the precipitation. The simulated time series of temperature and precipitation were obtained by the EBU-POM model for the given period. The observed time series of temperature and precipitations for two stations: Sombor (SO) (88 m.a.s.l.) and Novi Sad (NS) (84 m.a.s.l.) in the considered area, were taken from daily meteorological reports of the Republic Hydrometeorological Service of Serbia. For both sites, the modelled complexity is lower than the observed one, but with the reliability which is in the interval values allowed by the information measures (KC, KCM, and SE) [30, 31, 32]. *The term model reliability we have used in the*

following context. The Lyapunov exponent (LLE) relates to the predictability of measured time series, which includes deterministic chaos as an inherent component. Model predictability is here understood as the degree to which a correct prediction of a system's state can be made either qualitatively or quantitatively. In a stochastic analysis, a random process is considered predictable if it is possible to infer the next state from previous observations. In many models, however, randomness is a phenomenon which "spoils" predictability (Mihailović, 2019). Deterministic chaos does not mechanically denote total predictability but means that at least it improves the prognostic power. In contrast, stochastic trajectories cannot be projected into the future. If $LLE > 1$ *then time series is not chaotic, but is rather stochastic, and predictions cannot be based on chaos theory. However, if* $0 < LLE < 1$ *it indicatesthe existence of chaos in time series. In that case, one can compute the approximate time (often called Lyapunov time (LT)) limit for which accurate prediction for a chaotic system is a function of LLE. It designates a period when a specific process (physical, mechanical, hydrological, quantum, or even biological) moves beyond the bounds of precise (or probabilistic) predictability and enters a chaotic mode. According to (Frison and Abarbanel (1997) that time can be calculated as LLE* $\Delta t_{lyap} = 1/LLE$. *If* $LLE \to 0$, *implying that* $\Delta t_{lyap} \to \infty$, *then long-term accurate predictions are possible. However, many climate time series are highly complex. Therefore,* Δt_{lyap}*can be corrected for randomness in the following way. Similar to* Δt_{lyap} *we can introduce a randomness time* $\Delta t_{rand} = 1/KC$*(in time units, second, hour or day). Henceforth, we shall denote this quantity Kolmogorov time (KT), as it quantifies the period beyond which randomness significantly influences predictability. Then, the Lyapunov time corrected for randomness is defined as*$\left[0, \Delta t_{lyap}\right] \cap \left[0, \Delta t_{rand}\right]$. *It can be stated that the KT designates the size of the time window within time series where complexity remains nearly unchanged.* These findings mean that the models with a KC (and KCM) complexity lower than the measured time series complexity cannot always reconstruct some of the structures contained in the observed data. However, it does not mean that outputs from EBU-POM model do not correctly simulate climate elements since both sites values indicate the absence of stochastic influences, providing reliable projections of the climate elements.

Mihailović DT, Nikolić-Đorić E, Malinović-Milićević S, Singh VP, Mihailović A, Stošić T et al. The Choice of an Appropriate Information Dissimilarity Measure for Hierarchical Clustering of River Streamflow Time Series, Based on Calculated Lyapunov Exponent and Kolmogorov Measures. Entropy. 2019; 21: 215.

Frison TW, Abarbanel HDI. Ocean gravity waves: A nonlinear analysis of observations. Geophys Res. 1997; 102(C1): 1051–1059. DOI:10.1029/96JC02993.

Other comments:
Line 120: Please add a reference to back up your 'affirmation' of RCP scenarios without any storyline behind them.

Response:
Current version: In the Fifth Assessment Report (AR5), the Representative Concentration Pathway (RCP) is introduced, which are possible future concentration pathways without any storyline behind them.

New version: *In the Fifth Assessment Report (AR5), the Representative Concentration Pathway (RCP) is introduced, which are possible future concentration pathways without any storyline behind them (Moss et al., 2008).*

Moss R, Babiker M, Brinkman S, Calvo E, Carter T, Edmonds J et al. Towards new scenarios for analysis of emissions, climate change, impacts, and response strategies. IPCC Expert Meeting Report on New Scenarios. Intergovernmental Panel on Climate Change, Geneva. 2008. pp. 1–132

Line 150: Add a comma after 'In all years,'
Changed as suggested.

Lines 186-187: 'relative humidity' should be another item, i.e. (iv), you are treating it like that in the results.
Changed as suggested.

Line 188: Separate '2010 to'
Changed as suggested.

Line 219: Please include here the reference of "Vandenbroucke JP, Pearce N. Incidence rates in dynamic populations. Int J Epidemiol. 2012;41(5):1472–1479. "
Changed as suggested.

Line 219: I would suggest also separating periods of time using the 'en dash' that is: 2004–2005 instead of 2004-2005. This should be reviewed in the overall paper for consistency, there are places where you indeed use this but then you use a different format (e.g., line 227).
Changed as suggested.

Lines 224-226: I will strongly recommend authors moving the 'Empirical Formula' section to this section of the manuscript. It feels natural to introduce both the terminology and the corresponding formulas in the section discussing melanoma; currently the formula is between the description of the regional model and the environmental sampling of mosquitos, which is off topic for that section. Also, here you are introducing another formula derived from the previous one, which will benefit being close together in your narrative.
The subtitle "Empiricakl Formula" is erased, the text removed and inserted after "… (Bantaski Karlovac), and BG (Beograd)]."within the section Melanoma Incidence.

Line 230: KC spectrum should be in square brackets, is an abbreviation-like expression that you are using inside a parenthesis.

Response:

Current version: ... in which Kolmogorov complexity measures (Kolmogorov complexity (KC), Kolmogorov complexity spectrum KC spectrum) and the highest value of the KC spectrum (KCM)) and sample ...

New version: *... in which Kolmogorov complexity measures [Kolmogorov complexity (KC), Kolmogorov complexity spectrum (KC spectrum), and the highest value of the KC spectrum (KCM)], and sample ...*

Line 282: Add the word statistically: 'Toa, which is statistically significant'

Changed as suggested.

Line 283: Please be consistent in the way you define your variables across the manuscript. Sometimes you call overwintering temperature as Toa, others ToA, others you use sub indices, others you use the full letters. Same for the thousands, sometimes you separate them with ',' others no comma is used (e.g., 10,000 vs 10000).

Changed as suggested.

Line 315: Change the wording, 'mosquito vectors' to An. hyrcanus, you are not referencing to other vectors in the paper.

Here we refer also to Culex pipiens. The sentence remained as it is.

Line 326: Add comma after the word 'predict'.

Changed as suggested.

Line 386-387: Improve the wording; the word 'found' should be after the noun. For example: '…in 8 out of 81 dead wild birds found in Serbia […]. Each year WNV nucleic acid was detected in dead or captured wild birds found during summer time'. This second 'found' could even be eliminated.

Response:

Current version: From the first detection of WNV in 8 out of 81 found dead wild birds in Serbia [40], each year WNV nucleic acid was detected in found dead or captured wild birds during summertime [8].

New version: *From the first detection of WNV in 8 out of 81 dead wild birds found in Serbia [40], each year WNV nucleic acid was detected in dead or captured wild birds during summertime [8].*

Line 398: How did you center the ellipsoids for Fig5? You should describe this either on the main text or on the legend of the figure. From the current version, apparently the ellipsoids were developed considering a cluster of human cases right? This is not clear.

The figure caption was adapted as suggested:

Current version: Fig 5. Frequency of sampling of WNV infected mosquitoes (1–5 times, coloured numbers) during the period 2010–2016, superimposed over a cluster of mosquito, bird, horse, and human WNV cases in (a) 2014 and (b) 2015 (modified after Petrić et al. [9]).

New version: Fig 5. Frequency of sampling of WNV infected mosquitoes (1–5 times, coloured numbers) during the period 2010–2016, superimposed over a cluster of mosquito, bird, horse, and human WNV cases in (a) 2014 and (b) 2015. *The standard deviation ellipse (1-σ) is centered around the mean coordinates of the total number of mosquito, bird, horse and human WNV cases (modified after Petrić et al. [9]).*

Line 405: Use the word 'means' instead of 'mean'.
Changed as suggested.

Fig 1B: Please consider changing the color ramp of the altitude of the study region, green-red is not color-blind friendly.
Adapted:

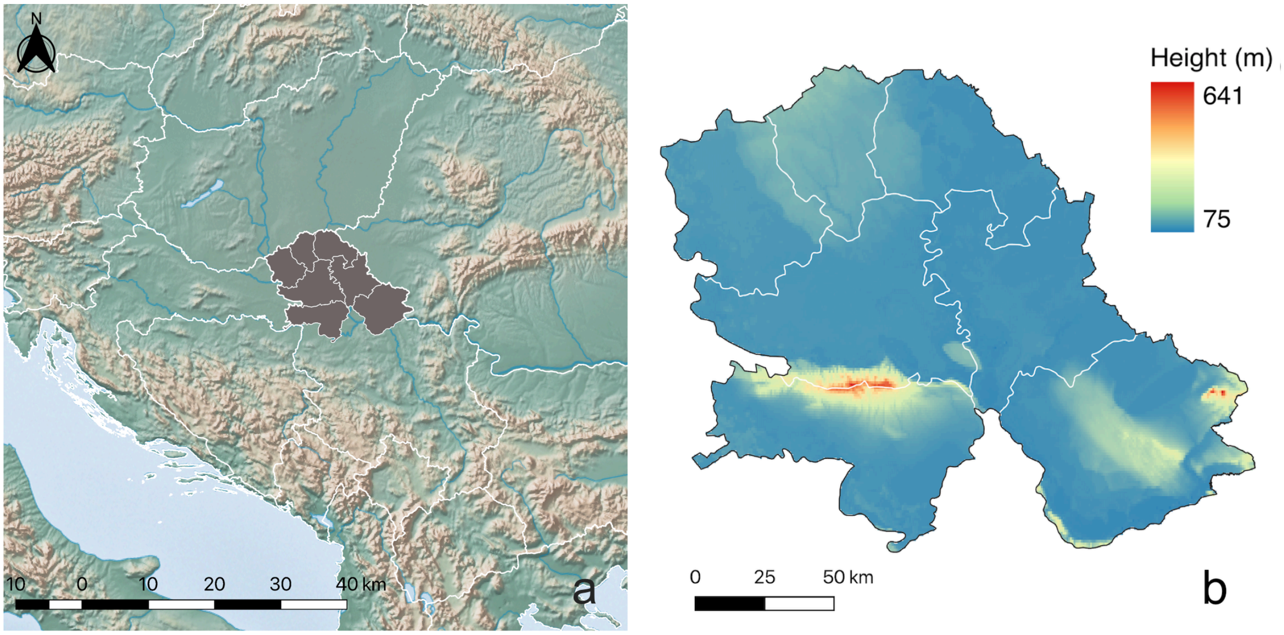

Supplement: Attachment — Submitted filename: Mihailovic2019_Response_to_Reviewers.pdf [file pone.0227679.s008.pdf]
