# Supplementary material for: Assessment of climate change impact on the malaria vector Anopheles hyrcanus, West Nile disease, and incidence of melanoma in the Vojvodina Province (Serbia) using data from a regional climate model
Source: PLoS One. 2020 Jan 15;15(1):e0227679. doi: 10.1371/journal.pone.0227679 (PMC6961917; doi:10.1371/journal.pone.0227679)
Supplement: Attachment — Submitted filename: Mihailovic2019_Response_to_Reviewers.pdf [file pone.0227679.s009.pdf]

Journal: PLOS ONE

Manuscript#: PONE-D-19-16900R3

Title of paper: Assessment of climate change impact on the malaria vector *Anopheles hyrcanus*, West Nile disease, and incidence of melanoma in the Vojvodina Province (Serbia) using data from a regional climate model

Dragutin T. Mihailović[1], Dušan Petrić[2*], Tamaš Petrović[3], Ivana Hrnjaković-Cvjetković[4,5], Vladimir Djurdjevic[6], Emilija Nikolić-Đorić[7], Ilija Arsenić[1], Mina Petrić[8,9,10*], Gordan Mimić[11], Aleksandra Ignjatović-Ćupina[2]

Dear Dr Samy,

We are pleased to submit the revised version of "Assessment of climate change impact on the malaria vector *Anopheles hyrcanus*, West Nile disease, and incidence of melanoma in the Vojvodina Province (Serbia) using data from a regional climate model" (#PONE-D-19-16900R3). We appreciate the time and efforts by the editor and advisors in reviewing the manuscript. Please find below detailed responses to the reviewers, whom we thank for their careful consideration of the manuscript. We also reviewed the manuscript for any additional errors and made small changes that are tracked in the attached document ("Revised Manuscript with Track Changes").

Reviewers' comments:

Reviewer #2:
Line 35: separate '10years' Corrected
Line 56: add a space after 'World Health Organization' Corrected
Line 66: to say that West Nile virus is one of the most detrimental vector borne diseases worldwide is debatable, potentially, dengue is far more detrimental worldwide. On the contrary, malaria is the most detrimental vector borne disease worldwide. Modify accordingly.

Response:
Our intention was to say that West Nile virus disease is one of the most detrimental vector-borne diseases worldwide, but construction of the sentence might indicate to the reader that malaria is the first and WNV disease the second most important.
The text was adapted accordingly. … are vectors of malaria and West Nile virus (WNV) disease, respectively, the two vector-borne diseases distributed worldwide [10,11].

Line 110: '…the Representative Concentration Pathways (RCPs) are introduced, which are…', use the plural form. Corrected
Line 161 (158): add a space in 'Figs2a' Corrected
Line 193 (189): space in 'Fig3' Corrected
Line 211 (208): add a comma after 'sites' Corrected
Line 213 (210): Change 'Firstly' for 'First' Corrected
Line 226-227 (223-224): Change 'N' for 'n' to describe sample size as done previously in the manuscript Corrected
Line 262 (261: use the plural form 'trends' Corrected

Reviewer #2: Lines 291-258: I appreciate the clear explanation of the authors for this part of the manuscript. However, I reviewed previous reviews and noticed that although now all the explanation is clear, no results are presented. Is clear that the authors are comparing EBU-POM model vs. the Republic Hydrometeorological Service of Serbia, but what is the conclusion? Is the model reliable? To use their own terminology: what are the results for all their comparisons: LLE Δtlyap = 1/LLE?, Δtrand = 1/KC?, what is the intersection between 0,Δtlyap and 0,Δtrand?. Another way to show this information would be: can the EBU-POM model be described by a deterministic chaotic equation? if so, is this demonstrated by this comparison? How all this is related to the overall manuscript. Does this means that that the regional model can be trusted? All these points are raised between these lines and never discussed again.

Response:
Thanks a lot for your remarks. They point out on your minutious and patient reading the manuscript. Today, it is rare to find such a collegial and professional trait. After careful reading your comments and also ones from the first round of the reviewing process, I have an impression that "we are (you as the reviewer and I as one of the authors) on the same line having a disconnection just in one point". It seems that our additional text inserted in the manuscript, can make an impression on the reader which is sublimated in the dictum: "she/he cannot see the forest for the trees". Maybe, partly you are right since your remark is quite "practical", while my elaboration is more "theoretical" without clearly emphasizing the touch down. I have tried to connect our half-lines in the aforementioned point.

Reviewer #2: However, I reviewed previous reviews and noticed that although now all the explanation is clear, no results are presented. Is clear that the authors are comparing EBU-POM model vs. the Republic Hydrometeorological Service of Serbia, but what is the conclusion?

Response:
You are right. After this text an additional conclusive statement is needed. In the revised version it is done in the following way: after the last statement in the text (line 258) we inserted the following text (Lines 255-257): *"Therefore, the EBU-POM model can be considered as a model having high performances and reliability in projection of temperature and participation, two of the most important elements used in different climate research."*

Reviewer #2: Is the model reliable? To use their own terminology: what are the results for all their comparisons: LLE Δtlyap = 1/LLE?, Δtrand = 1/KC?, what is the intersection between 0,Δtlyap and 0,Δtrand?. Another way to show this information would be: can the EBU-POM model be described by a deterministic chaotic equation? If so, is this demonstrated by this comparison? How all this is related to the overall manuscript.

Now we see where a potential source of misunderstanding, which is partly introduced by our omitting a conclusive text about EBU-POM model performances (that is done in the revised version). It was your question about meaning of the term - model reliability in the previous reviewing report. To explain that we inserted the text about Lyapunov and Kolmogorov times as the indicators of the model reliability. Certainly, that EBU-POM model equations satisfy conditions that come from *LT* and *KT*, i.e. cannot "take away" system into chaos. Therefore, no necessary tests in this paper are needed. More details about this issue can be found in Mihailović et al. (2014) which is included in the text (Line 255) and the reference list.

Mihailovic DT, Mimic G, Arsenic I. Climate predictions: the chaos and complexity in climate models, Advances in Meteorology. 2014; DOI: 10.1155/2014/878249.

Reviewer #2: Does this means that that the regional model can be trusted? All these points are raised between these lines and never discussed again.

Response:
Certainly, that the EBU-POM model is trustable. Moreover, it can be included in the group of frequently used models.

New version: We considered the papers by Mihailović et al. [2,30] in which Kolmogorov complexity measures [Kolmogorov complexity (KC), Kolmogorov complexity spectrum (KC spectrum), and the highest value of the KC spectrum (KCM)], and sample entropy (SE) [31] were used to quantify the regularity and complexity of air temperature and precipitation time series, obtained by the EBU-POM model, representing both deterministic chaos and stochastic processes. We considered the complexity of the EBU-POM model using the observed and modelled time series of temperature and precipitation. We computed the KC spectrum, KC, KCM and SE values for temperature and precipitation. The calculations were performed for the entire time interval 1961–1990: (i) on a daily basis with a size of n=10,958 samples for temperature and (ii) on a monthly basis with a size N=360 for the precipitation. The simulated time series of temperature and precipitation were obtained by the EBU-POM model for the given period. The observed time series of temperature and precipitations for two stations: Sombor (SO) (88 m.a.s.l.) and Novi Sad (NS) (84 m.a.s.l.) in the considered area, were taken from daily meteorological reports of the Republic Hydrometeorological Service of Serbia. For both sites, the modelled complexity is lower than the observed one, but with the reliability which is in the interval values allowed by the information measures (KC, KCM, and SE)

[32,33,34]. The term model reliability we have used in the following context. The Lyapunov exponent (LLE) relates to the predictability of measured time series, which includes deterministic chaos as an inherent component. Model predictability is here understood as the degree to which a correct prediction of a system's state can be made either qualitatively or quantitatively. In a stochastic analysis, a random process is considered predictable if it is possible to infer the next state from previous observations. In many models, however, randomness is a phenomenon which "spoils" predictability [35]. Deterministic chaos does not mechanically denote total predictability but means that at least it improves the prognostic power. In contrast, stochastic trajectories cannot be projected into the future. If $\mathrm{LLE} > 1$ then time series is not chaotic, but is rather stochastic, and predictions cannot be based on chaos theory. However, if $0 < LLE < 1$ it indicates the existence of chaos in time series. In that case, one can compute the approximate time [often called Lyapunov time (LT)] limit for which accurate prediction for a chaotic system is a function of LLE. It designates a period when a specific process (physical, mechanical, hydrological, quantum, or even biological) moves beyond the bounds of precise (or probabilistic) predictability and enters a chaotic mode. According to Frison and Abarbanel [36] that time can be calculated as LLE $\Delta t_{\mathrm{lyap}} = 1/\mathrm{LLE}$. If $\mathrm{LLE} \to 0$, implying that $\Delta t_{\mathrm{lyap}} \to \infty$, then long-term accurate predictions are possible. However, many climate time series are highly complex. Therefore, Δt_{lyap} can be corrected for randomness in the following way. Similar to Δt_{lyap} we can introduce a randomness time $\Delta t_{\mathrm{rand}} = 1/\mathrm{KC}$ (in time units, second, hour or day). Henceforth, we shall denote this quantity Kolmogorov time (KT), as it quantifies the period beyond which randomness significantly influences predictability. Then, the Lyapunov time corrected for randomness is defined as $[0, \Delta t_{\mathrm{lyap}}] \cap [0, \Delta t_{\mathrm{rand}}]$. It can be stated that the KT designates the size of the time window within time series where complexity remains nearly unchanged. These findings mean that the models with a KC (and KCM) complexity lower than the measured time series complexity cannot always reconstruct some of the structures contained in the observed data. However, it does not mean that outputs from EBU-POM model do not correctly simulate climate elements since both sites' values indicate the absence of stochastic influences, providing reliable projections of the climate elements *(37 -Mihailovic et al. 2014). Therefore, the EBU-POM model can be considered as a model having high performances and reliability in projection of temperature and precipitation, two of the most important elements used in different climate research.*

Line 362: Anopheles has already been written, change to "An. maculipennis" Corrected

Lines 457-459: Please add these lines after the corresponding discussion of environmental models in the discussion section (that is, after line 348). Corrected
