# Supplementary material for: Assessment of climate change impact on the malaria vector Anopheles hyrcanus, West Nile disease, and incidence of melanoma in the Vojvodina Province (Serbia) using data from a regional climate model
Source: PLoS One. 2020 Jan 15;15(1):e0227679. doi: 10.1371/journal.pone.0227679 (PMC6961917; doi:10.1371/journal.pone.0227679)
Supplement: Attachment — Submitted filename: Comments on PONE-D-19-16900.docx [file pone.0227679.s004.docx]

Comments on the manuscript PONE-D-19-16900

Title: Assessment of climate change impact on malaria vectors, West Nile disease, and incidence of melanoma in the Vojvodina Province (Serbia) using data from a regional climate model

Authors: Dragutin T. Mihailović, Dušan Petrić, Tamaš Petrović, Ivana Hrnjaković Cvjetković, Vladimir Đurđević, Emilija Nikolić Đorić, Ilija Arsenić, Mina Petrić, Gordan Mimić

The work presented in paper Mihailović et al. is interesting. The objective of the authors was to compare data from the mosquito field collections and medical studies with regional 29 climate model projections to examine the impact of climate change on the circulation of West Nile virus (WNV), the spreading of the malaria vector *Anopheles hyrcanus* and the incidence of melanoma. The comparison was done with the coupled regional Eta Belgrade University and Princeton Ocean Model for the period 1961-2015 using the A1B scenario, and the expected changes up to 2030. Overall, significant correlation was found between the frequency of WNV in *Culex pipiens* and the overwintering temperature averages and seasonal relative humidity at the sampling sites. Correlation was also found between the spreading and relative abundance of *Anopheles hyrcanus* and the trend of the mean annual temperature. There was also an increase in melanoma incidence.

Minor comments to authors

**Title**

Authors wrote “malaria vectors” but the only presented data on only one vector *Anopheles hyrcanus*

**Abstract**

L32: ……………and 10-years. Delete the hyphen

L37: ……………*Culex. pipiens*. Delete the dot

**Introduction**

L49-53: Are the authors referring to themselves when they stated, “The authors (……….), have been working together…….”?

L72: Authors should write, “Climate change……..” instead of “The climate change……..”

L75: Author should write, “Melanoma mortality…………..within the period 1985-2004

L78: Authors should define the acronym ENCR, as this is used here for the first time.

L81-82: Authors should use past tense in the sentence “……we compared considerable of previously……”

L54-55: This sentence is not clear for me. I suggest this: “In this paper, we analysed observed data collected over a period of 31 years……”

**Materials and Methods**

L96: Authors should define SRES-A1B scenario for the first time.

L115: Authors used only one formula, the subtitle should be in singular

**Results**

- Authors should specify the exact p-values instead of writing p<0.05 or p>0.05

L207: “The Poisson regression model for the dependence of a number of detections per site (frequency-λ)…………………………is highly significant”. Authors stated it was highly significant, but from my perspective, p<0.05 is not a specific indication of high significance. Could the give the exact value of p?

- Fig2b and 2c are fuzzy
- Fig4. Colors of fig4c are confusing

It will be more interesting if the authors used only vector-borne diseases data in this paper.

**N.B: Other comments are incorporated in the manuscript**
